# Global gene expression patterns of grass carp following compensatory growth

**DOI:** 10.1186/s12864-015-1427-2

**Published:** 2015-03-14

**Authors:** Libo He, Yongyan Pei, Yao Jiang, Yongming Li, Lanjie Liao, Zuoyan Zhu, Yaping Wang

**Affiliations:** State Key Laboratory of Freshwater Ecology and Biotechnology, Institute of Hydrobiology, Chinese Academy of Sciences, Wuhan, 430072 China; University of Chinese Academy of Sciences, Beijing, 100049 China

**Keywords:** Grass carp, Compensatory growth, Transcriptome analysis, RNA sequencing, Fasting, Re-feeding, Differentially expressed genes

## Abstract

**Background:**

Compensatory growth is accelerated compared with normal growth and occurs when growth-limiting conditions are overcome. Most animals, especially fish, are capable of compensatory growth, but the mechanisms remain unclear. Further investigation of the mechanism of compensatory growth in fish is needed to improve feeding efficiency, reduce cost, and explore growth-related genes.

**Results:**

In the study, grass carp, an important farmed fish in China, were subjected to a compensatory growth experiment followed by transcriptome analysis by RNA-sequencing. Samples of fish from starved and re-feeding conditions were compared with the control. Under starved conditions, 4061 and 1988 differentially expressed genes (DEGs) were detected in muscle and liver tissue when compared the experimental group with control group, respectively. After re-feeding, 349 and 247 DEGs were identified in muscle and liver when the two groups were compared. Moreover, when samples from experimental group in starved and re-feeding conditions were compared, 4903 and 2444 DEGs were found in muscle and liver. Most of these DEGs were involved in metabolic processes, or encoded enzymes or proteins with catalytic activity or binding functions, or involved in metabolic and biosynthetic pathways. A number of the more significant DEGs were subjected to further analysis. Under fasting conditions, many up-regulated genes were associated with protein ubiquitination or degradation, whereas many down-regulated genes were involved in the metabolism of glucose and fatty acids. Under re-feeding conditions, genes participating in muscle synthesis and fatty acid metabolism were up-regulated significantly, and genes related to protein ubiquitination or degradation were down-regulated. Moreover, Several DEGs were random selected for confirmation by real-time quantitative PCR.

**Conclusions:**

Global gene expression patterns of grass carp during compensatory growth were determined. To our knowledge, this is a first reported for a teleost fish. The results will enhance our understanding of the mechanism of compensatory growth in teleost fish.

**Electronic supplementary material:**

The online version of this article (doi:10.1186/s12864-015-1427-2) contains supplementary material, which is available to authorized users.

## Background

Growth is an important trait in fish farming and is one of the primary targets in breeding programs aimed at improving productivity [1]. Growth of fish is governed by multiple genes and is also influenced by various environmental factors [2]. Several important growth-related genes have been identified in various domesticated vertebrates, including growth hormone (*GH*), growth hormone receptor (*GHR*), insulin-like growth factor (*IGF*) I and II, growth hormone-releasing hormone (*GHRH*), *leptins*, growth hormone inhibiting hormone (*GHIH*), myostatin (*MSTN*), myogenic regulatory factors (*MRFs*), and many others [3,4]. However, reports on other growth-related genes in fish are relatively limited. Quantitative trait loci (QTLs) have been successfully used to locate growth-associated genes to particular regions of the fish genome, but identifying individual genes has proved difficult [5-8]. Other methods such as compensatory growth experiments proved fruitful for understanding regulatory mechanisms connected with growth in fish [9-12].

Compensatory growth is a period of accelerated growth that follows growth-limiting conditions once non-limiting conditions are renewed [13]. Characteristic features of compensatory growth include increased food-intake, accelerated mitosis and enhanced rate of food utilization. Compensatory growth was first reported a century ago and has been widely studied in vertebrates [14]. Most animals, especially fish, are capable of compensatory growth [15]. However, the regulatory mechanisms and global gene expression patterns of compensatory growth in fish remain poorly understood. Expanding knowledge in this area is important for identifying growth-associated genes, increasing the efficiency of feeding and reducing the cost of fish farming.

Transcriptome or RNA-sequencing (RNA-seq) is a technology based on next-generation sequencing that is being successfully applied to transcriptome analysis [16]. RNA-seq has proved advantageous for characterizing the gene expression profiles of both model and non-model species, despite only being available for a short time [17,18]. Moreover, RNA-seq has strengthened our understanding of the breadth and depth of eukaryotic transcriptomes. In fish, transcriptome profiles can be mapped and annotated by RNA-seq, and numerous biological processes such as development, host immune response, stress response, and adaptive evolution are now better understood due to this technique [19]. RNA-seq has been applied to zebrafish, channel catfish, European sea bass, rainbow trout, and grass carp [20-24].

Grass carp (*Ctenopharyngodon idellus*), an important aquaculture species in China, accounts for more than 18% of total freshwater aquaculture production in this country. Production of grass carp reached 478.2 million tons in 2012, which making it the most highly consumed freshwater fish worldwide [25]. However, much of our knowledge on grass carp genes is restricted to immunity-related or disease resistance-associated genes, and growth–related genes are not well understood [26-29]. Therefore, it is important to explore growth-related genes to inform grass carp breeding programs aimed at improving growth traits. To this end, grass carp were subjected to compensatory growth and transcriptome analysis by RNA-seq in this study. Global gene expression patterns during compensatory growth were investigated, and some significant differentially expressed genes (DEGs) were identified and annotated. These results enhance our understanding of the mechanism of compensatory growth in grass carp and will be of benefit to future grass carp breeding programs.

## Methods

### Ethical procedures

Animal welfare and experimental procedures were carried out in accordance with the Guide for the Care and Use of Laboratory Animals (Ministry of Science and Technology of China, 2006), and the protocol was approved by the committee of the Institute of Hydrobiology, Chinese Academy of Sciences (CAS). All surgery was performed under eugenol anesthesia, and all efforts were made to minimize suffering.

### Experimental fish

Healthy grass carp at three months old were used in the study. Grass carp weighing 2–3 g and an average length of 5 cm were obtained from the Guan Qiao Experimental Station, Institute of Hydrobiology, CAS, and acclimatized in aerated fresh water at 26-28°C for one week before processing. Fish were fed with commercial feed (The component of the feed that used in the study was provided in Additional file 1) twice a day and water was exchanged daily. If no abnormal symptoms were observed, grass carp were selected for further study. Fish were divided into control and experimental groups that each included three repeated subgroups. Each subgroup contained 65 grass carp in a separate tank. Five fish were sampled in each repeated subgroups at each time point.

### Compensatory growth experiment

The compensatory growth experiment was carried out after no abnormal symptom were observed, and lasted for five weeks. At the beginning of the experiment, fish in the two groups were weighed. During the first week, fish in the control group were fed twice a day, whereas no feed was given to the experimental group. After the first week, five fish from each subgroup were weighed and muscle and liver were sampled. These samples were named as C-1-M (control, first week, muscle), C-l-L (control, first week, Liver), E-1-M (experimental, first week, muscle), and E-1-L (experimental, first week, Liver). In the second week, fish in both groups were fed twice a day, and fish were weighed and sampled at the end of the second week as described. These were named as C-2-M (control, second week, muscle), C-2-L (control, second week, Liver), E-2-M (experimental, second week, muscle), and E-2-L (experimental, second week, Liver). Tissue samples from the same subgroups were mixed equivalently for RNA isolation. The remaining fish were fed twice a day and weighed every week until the end of the experiment, and weights were subjected to statistical analysis. The Specific growth rate (SGR) was calculated as described previously [12]. Briefly, SGR means ((In W_2_-W_1_)/(T_2_-T_1_) × 100), where W_2_ is the weight at the end of the growth interval and W_1_ is the weight at the beginning of the growth interval, while T_2_-T_1_ represents the duration (days) of the growing interval. In this study, SGRs were calculated for control and experimental group during the three time intervals: 0 ~ 1 weeks, 1 ~ 2 weeks, and 2 ~ 5 weeks. In addition, an independent repeated experiment was carried out by the similar method that described above. The repeated experiment was lasted for three weeks and samples were used for qPCR analysis.

### RNA isolation, library construction and sequencing

RNA was isolated using Trizol reagent (Invitrogen, USA) according the manufacturer’s protocol, concentration was measured by the Qubit RNA assay kit (Life Technologies, USA), and integrity was assessed with the RNA nano 6000 assay kit (Agilent Technologies, USA). RNA of sufficient quality was used in library construction. Sequencing libraries were generated using the NEBNext Ultra RNA library prep kit for Illumina (New England Biolabs, USA) following the manufacturer’s protocol. Briefly, mRNA was purified from total RNA using poly-T oligo-attached magnetic beads and fragmented by NEBNext first strand synthesis reaction buffer (New England Biolabs, USA). First strand cDNA was synthesized using a random hexamer primer and M-MuLV reverse transcriptase. Second strand cDNA synthesis was subsequently performed using DNA polymerase I and RNase H. After adenylation of the 3’ end of DNA fragments, NEBNext adaptors with hairpin loop structure were ligated in preparation for hybridization. 3 μl USER enzyme (New England Biolabs, USA) was used with size-selected, adaptor-ligated cDNA at 37°C for 15 min followed by 5 min at 95°C prior to PCR using phusion high-fidelity DNA polymerase, universal PCR primers and index (X) primer. Finally, PCR products were purified using an AMPure XP system and library quality was assessed using an Agilent Bioanalyzer 2100 system. Libraries were sequenced on an Illumina Hiseq 2000 platform and 100 bp single-end reads were generated.

### Data analysis

Raw data reads in fastq format were initially processed using in-house perl scripts. In this step, clean data (clean reads) were obtained by removing adapter, poly-N and poor quality data. The Q20, Q30, and GC content of the clean data were calculated, and all downstream analysis was performed the clean high quality data.

Clean data were mapped to the grass carp reference genome (Bioproject: PRJNA39737, unpublished data) using TopHat2 software [30]. Two base mismatches were allowed in the mapping process, total mapped reads were calculated, and the mapped regions (exon, intron, and intergenic) were counted.

HTSeq software was used to count the number of reads mapped to each gene [31], and the reads per kilobase of the exon model per million mapped reads (RPKM) were calculated for each gene based on the length of the gene and the number of reads mapped to the gene [32].

### Differential expression analysis

Differential expression analysis of two groups/conditions was performed using the DESeq package [33]. The resulting p-values were adjusted using the Benjamini and Hochberg’s approach for controlling the false discovery rate. Genes with an adjusted p-value <0.05 (padj <0.05) found by DESeq were assigned as differentially expressed.

Gene Ontology (GO) enrichment analysis of DEGs was implemented by the GOseq R package [34], in which gene length bias was corrected. GO terms with corrected p-values less than 0.05 were considered significantly enriched by DEGs.

The Kyoto Encyclopedia of Genes and Genomes (KEGG) database is used for understanding high-level functional information in biological systems from molecules, cells, organisms and ecosystems, and is particularly powerful for large-scale molecular datasets generated by genome sequencing and other high-throughput experimental approaches [35]. In this study, KOBAS software was employed to test the statistical enrichment of DEGs in KEGG pathways [36]. KEGG terms with corrected p-values less than 0.05 were considered significant.

### Validation of DEGs by qPCR

In order to confirm the reliability of data obtained by RNA-seq, twelve DEGs were random selected for validation by qPCR. The primers were listed in Additional file 2 and the cDNA sequences (completely or partially) of these genes were shown in Additional file 3. The RNA samples from an independent repeated study and were used for reverse transcription. First strand cDNAs were obtained using a random hexamer primer and the ReverTra Ace kit (Toyobo, Japan). qPCR was carried out in a Bio-rad fluorescence quantitative PCR instrument (Bio-rad, USA). Each qPCR mixture contained 0.8 μL sense and reverse primers, 1 μL template, 10 μL 2 × SYBR mix (TOYOBO, Japan), and 7.4 μL ddH_2_O. Three replicates were conducted for each sample and β-actin gene was used as an internal control to normalize. Only the primer with efficiency of 90% ~ 110% was used for qPCR. The program for qPCR was as follows: 95°C for 10 s, 40 cycles of 95°C for 5 s and 60°C for 20 s. Relative expression level was calculated using the 2^-△△^Ct method [37]. All data are given as mean ± standard deviation of three replicates.

## Results

### Changes in body weight and SGR during compensatory growth

**Figure 1:**
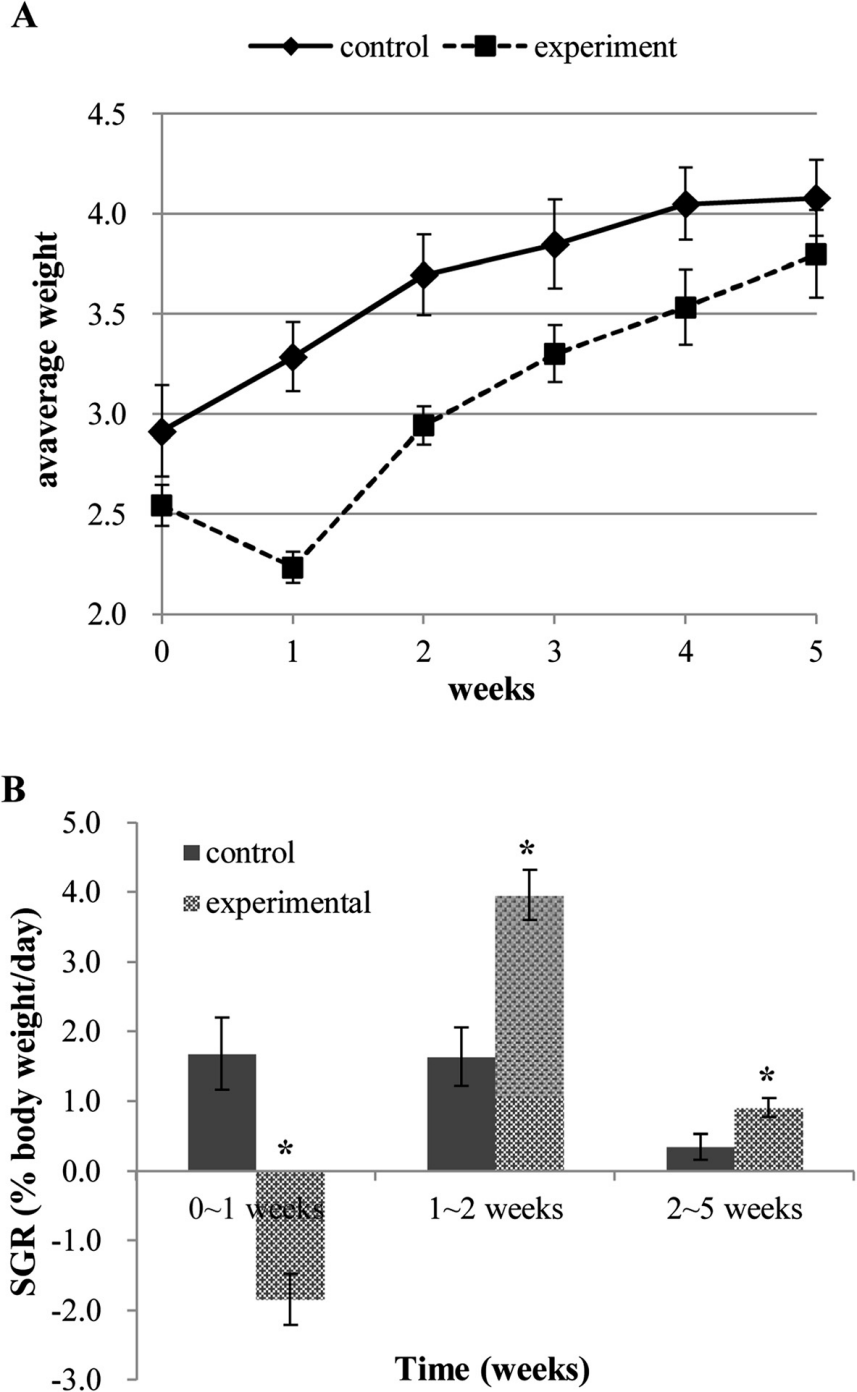
**Growth curve and SGR of grass carp during compensatory growth. (A)** Growth curve of grass carp during compensatory growth. Fish in experimental and control groups were weighted at six time points and the weights were subjected to curve drawn. In each time point, 15 grass carp from three subgroups were random selected and weighted. Data are given as mean ± standard deviation (S.D.). **(B)** SGR of grass carp during compensatory growth. SGRs were calculated for control and experimental group during the three time intervals: 0 ~ 1 weeks, 1 ~ 2 weeks, and 2 ~ 5 weeks. Asterisks represent significant differences between groups at each time intervals (P < 0.01) that calculated by *T* test.

The weight of fish in two groups was recorded at six time points and curves were drawn (Figure 1A). For the control group, a total increase of 1.16 g in body weight and a growth rate of 39.8% was obtained. For the experimental group, a total increase of 1.26 g in body weight and a growth rate of 49.5% was acquired. Moreover, the SGR in different time intervals was calculated (Figure 1B). In the first week, the weight of the experimental group decreased sharply following the induced starvation (12.2% decrease in body weight), indicated by a negative SGR (−1.84 ± 0.52). During the following week of re-feeding, the weight of the experimental group increased rapidly and resulted in a positive of SGR (3.95 ± 0.36), which is significantly higher than (P < 0.01) that in the control group (SGR = 1.64 ± 0.42). The elevated SGR that characterizes compensatory growth subsequently declined back to low level during the 2 ~ 5 weeks of realimentation, whereas the SGR of experimental group (SGR = 0.91 ± 0.14) was still significantly higher than (P < 0.01) that in the control group (SGR = 0.34 ± 0.19) (Figure 1B).

### Preliminary analysis of RNA-seq data

At different time points, muscle and liver samples from control and experimental groups were used for library construction. Duplicates were performed for each class, therefore 24 libraries were constructed in total. These libraries were sequenced using the Illumina Hiseq. 2000 platform, and raw reads, clean reads, Q20, total mapped reads, and unique mapped reads for each library were recorded (Table 1). All libraries gave Q20 ≥ 95%, total mapped reads ≥89%, and unique mapped reads ≥85%. Moreover, the percentage of the total mapped reads that mapped to the genome region was calculated and ≥83% mapped to the exon for all libraries (data not shown). This confirmed the high quality of the sequencing data and suitability for further analysis. The sequencing data in this study have been deposited in the Sequence Read Archive (SRA) at the National Center for Biotechnology Information (NCBI) (accession number: SRP055685).

**Table 1:** **Summary of sequencing data and mapped results in the study**

**Sampling conditions**	**Group**	**Tissue**	**Sample name**	**Duplicates**	**Raw reads**	**Clean reads**	**Q20**		
(%)	Total								
mapped									
reads	Uniquely								
mapped									
reads									
After									
fasting	control group	muscle	C-1-M	a	15314862	15128756	97.33	14059595 (92.93%)	13217716 (87.37%)
				b	14823783	14646957	97.41	13619629 (92.99%)	12843759 (87.69%)
				c	16321214	16109369	97.33	14958284 (92.85%)	14160730
(87.9%)									
		liver	C-1-L	a	14438249	14251753	96.49	13182134 (92.49%)	12514167 (87.81%)
				b	17127547	16903996	96.56	15592380 (92.24%)	14753416 (87.28%)
				c	14523380	14310106	96.5	13233306 (92.48%)	12636047
(88.3%)									
	experimental group	muscle	E-1-M	a	14988606	14778249	96.82	13616046 (92.14%)	13093152
(88.6%)									
				b	17791102	17541598	96.93	16214273 (92.43%)	15661812 (89.28%)
				c	14762762	14589546	96.98	13498507 (92.52%)	12890714 (88.36%)
		liver	E-1-L	a	16289196	16107092	97.55	14933900 (92.72%)	14405289 (89.43%)
				b	11491146	11345300	97.52	10536721 (92.87%)	10183044 (89.76%)
				c	17191329	16972319	97.38	15807590 (93.14%)	15308206
(90.2%)									
After re-feeding	control group	muscle	C-2-M	a	19323422	18945419	97.6	17275859 (91.19%)	16426146
(86.7%)									
				b	17903385	17565549	97.62	15988557 (91.02%)	15240472 (86.76%)
				c	21096997	20647765	97.63	18828972 (91.19%)	17985183
(87.1%)									
		liver	C-2-L	a	17502755	17349119	97.56	16078785 (92.68%)	15316916 (88.29%)
				b	17230237	17050272	97.52	15895193 (93.23%)	15188285 (89.08%)
				c	16617660	16399951	97.5	15177704 (92.55%)	14472205 (88.25%)
	experimental group	muscle	E-2-M	a	12796800	12303323	95.99	11089012 (90.13%)	10562927 (85.85%)
				b	20644903	19733726	95.82	17856999 (90.49%)	17012127 (86.21%)
				c	16427709	15639982	95.88	14047696 (89.82%)	13295885 (85.01%)
		liver	E-2-L	a	13892743	13713405	97.42	12794265 (93.3%)	12273895
(89.5%)									
				b	17895233	17661014	97.42	16454496 (93.17%)	15843241 (89.71%)
				c	15687125	15496141	97.43	14440618 (93.19%)	13845040 (89.35%)

### Analysis of gene expression level

The total reads number that mapped to the genome region of each gene was calculated using HTSeq software, and the expression level of each gene was calculated according to the method of RPKM [32]. RPKM intervals and gene numbers in each RPKM interval of all 24 libraries are listed in Additional file 4. RPKM intervals were similar for duplicate samples. However, RPKM intervals of samples from muscle were different from liver samples. The number of genes with a low RPKM interval (1 ~ 3) was greater in liver, whereas the number of genes with a high RPKM interval (15 ~ 60) was greater in muscle. The RPKM of each gene was calculated from the average of all replicates. Moreover, the R2 value of the Pearson product–moment correlation coefficient was ≥0.92 for all replicates (data not shown), which further confirmed the repeatability of the sampling.

### Identification of DEGs

**Figure 2:**
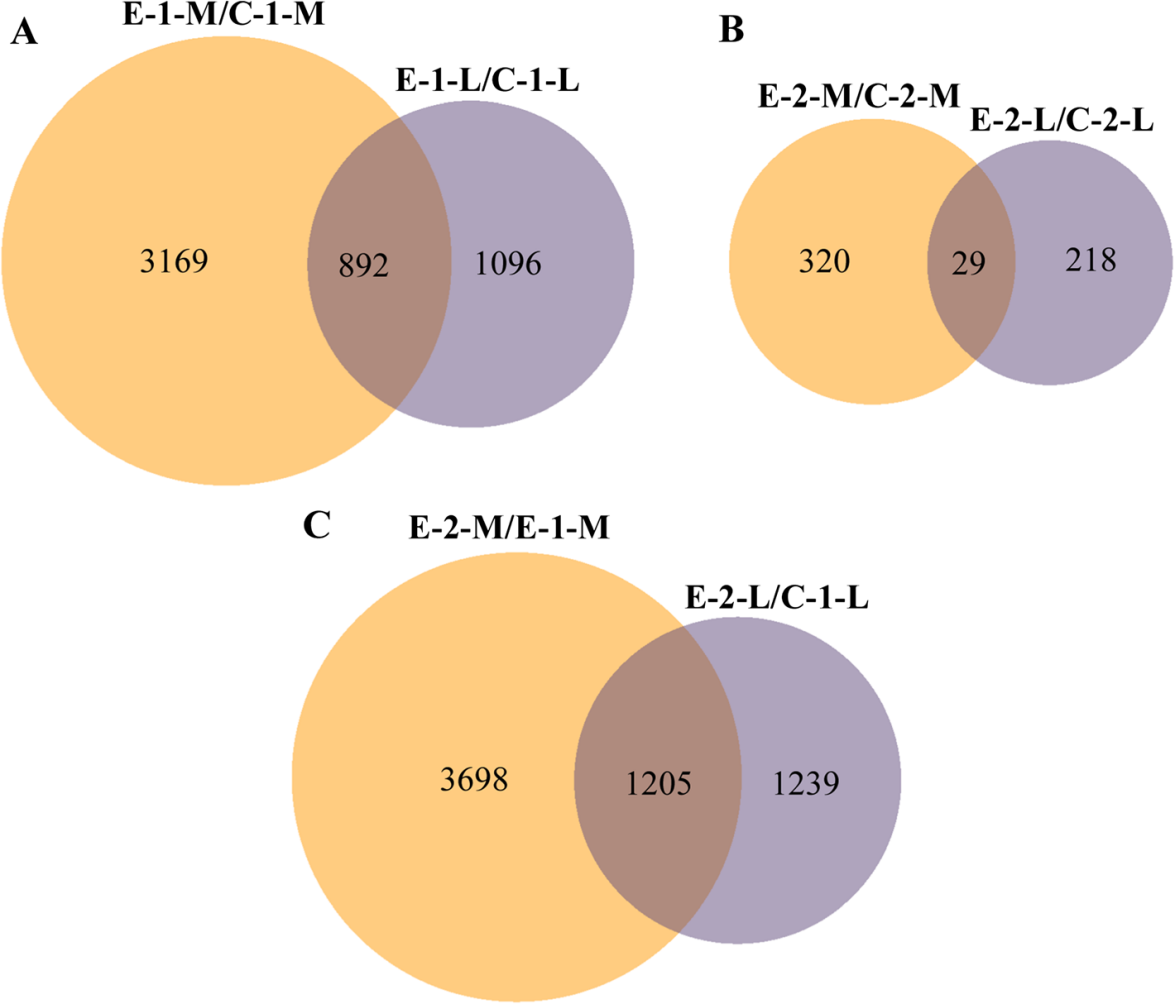
**Venn diagram of DEGs between different comparisons.** Overlapping regions represent DEGs in both comparisons. The size of the circle was made proportional to the number it represents. **(A)** Venn diagram of DEGs between E-1-M/C-1-M and E-1-L/C-1-L. **(B)** Venn diagram of DEGs between E-2-M/C-2-M and E-2-L/C-2-L. **(C)** Venn diagram of DEGs between E-2-M/E-1-M and E-2-L/E-1-L.

From integration of the replicates, the 24 libraries were condensed into 8 sample groups named C-1-M, C-l-L, E-1-M, E-1-L, C-2-M, C-2-L, E-2-M, and E-2-L. To identify DEGs, samples were subjected to a series of paired-comparisons. Muscle and liver samples from the experimental group that experienced fasting conditions were compared with the appropriate control group (E-1-M/C-1-M and E-1-L/C-1-L). Muscle and liver samples from the experimental group following re-feeding were compared with the appropriate control group (E-2-M/C-2-M and E-2-L/C-2-L). In addition, muscle and liver samples from the experimental group following re-feeding were compared with experimental groups following fasting conditions (E-2-M/E-1-M and E-2-L/E-1-L). The number of DEGs identified from the different paired-comparisons is listed in Table 2. In fasting conditions, 4061 DEGs were detected in muscle (2124 up-regulated and 1937 down-regulated) and 1988 DEGs were identified in liver (761 up-regulated and 1227 down-regulated). Following re-feeding, 349 and 27 DEGs were discovered in muscle (281 up-regulated and 68 down-regulated) and liver (148 up-regulated and 99 down-regulated), respectively. Moreover, when fasting and re-feeding experimental groups were compared, 4903 DEGs were identified in muscle (2668 up-regulated and 2235 down-regulated) and 2444 DEGs were detected in liver (1512 up-regulated and 932 down-regulated). Detailed information of all DEGs is shown in Additional file 5. These DEGs were subjected to Venn diagram analysis (Figure 2), which identified 892 genes in both E-1-M/C-1-M and E-1-L/C-1-L (Figure 2A), 29 genes in both E-2-M/C-2-M and E-2-L/C-2-L (Figure 2B), and 1205 genes in both E-2-M/E-1-M andE-2-L/E-1-L (Figure 2C).

**Table 2:** **Summary of DEGs in different comparison**

**Condition/group**	**Tissues**	**Comparison**	**DEGS**		
			**Up-regulated**	**Down-regulated**	**Total**
After	muscle	E-1-M/C-1-M	2124	1937	4061
fasting	liver	E-1-L/C-1-L	761	1227	1988
After re-feeding	muscle	E-2-M/C-2-M	281	68	349
	liver	E-2-L/C-2-L	148	99	247
Experimental group	muscle	E-2-M/E-1-M	2668	2235	4903
	liver	E-2-L/E-1-L	1512	932	2444

### GO enrichment analysis

**Figure 3:**
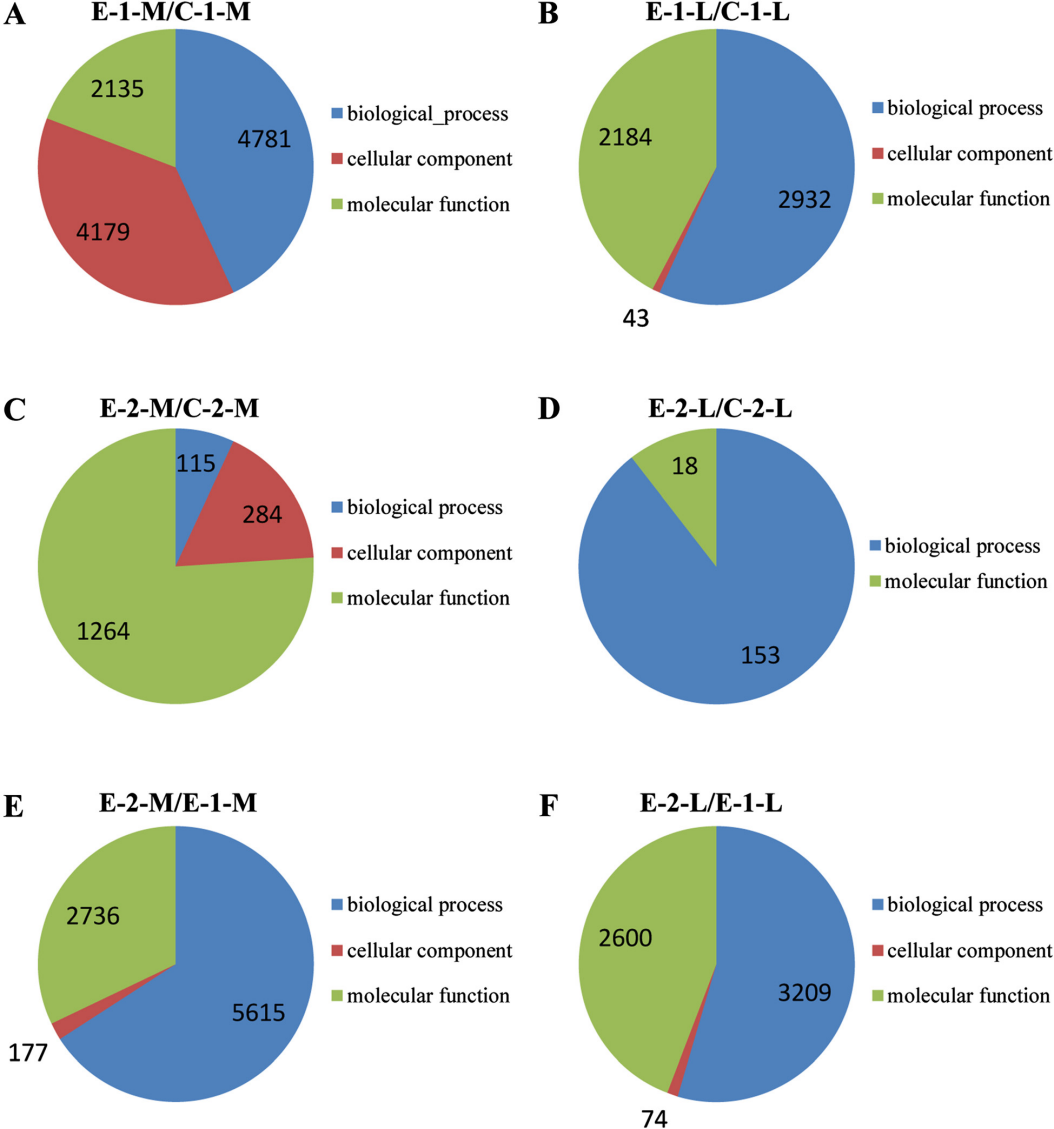
**Gene ontology of the top 30 enriched terms in different comparisons.** Annotated genes were placed in three main categories, namely biological process, molecular function, and cellular component. The number of genes in each comparison is shown. **A**, E-1-M/C-1-M; **B**, E-1-L/C-1-L; **C**, E-2-M/C-2-M; **D**, E-2-L/C-2-L; **E**, E-2-M/E-1-M; **F**, E-2-L/E-1-L.

GO enrichment analysis was performed to investigate the possible roles of DEGs. For all six paired-comparisons, annotated genes were categorized into three main categories, namely biological process, molecular function, and cellular component (Figure 3, top 30 most enriched terms). The biological process category included high representation for genes involved in single-organism metabolic processes, organonitrogen compound metabolism, small molecule metabolism, oxidation-reduction, general metabolic and organic acid metabolic processes. Catalytic activity, oxidoreductase activity, cofactor binding, coenzyme binding, and other binding terms were significant enriched in the molecular function category. In the cellular component category, intracellular, myosin complex, extracellular matrix, actin cytoskeleton, and non-membrane-bound organelle terms were abundant. In the comparison of E-2-L/C-2-L, no cellular component term was enriched, and the number of terms for biological process and molecular function was also low. Detailed information of enriched terms is listed in Additional file 6.

### KEGG analysis

In order to identify possible biochemical pathways in which DEGs operate, KEGG pathway analysis was carried out for all six paired-comparisons, and significantly enriched pathways are listed in Table 3. The most of enriched pathways were metabolism- or biosynthesis-related pathways such as metabolic pathways, carbon metabolism, fatty acid metabolism, glycine, serine and threonine metabolism, citrate cycle (TCA cycle), and biosynthesis of amino acids. In addition, some pathways involved in genetic information processing were also enriched, such as protein processing in endoplasmic reticulum, DNA replication, aminoacyl-tRNA biosynthesis, ribosome biogenesis in eukaryotes, and RNA transport. In all the significant enriched pathways, metabolic pathway was the top category that included the greatest number of annotated genes. In the comparison of E-2-M/C-2-M and E-2-L/C-2-L, fewer pathways were enriched, indicating less DEGs in these comparisons.

**Table 3:** **KEGG pathways of DEGs in different comparisons**

**KEGG term**	**E-1-M/C-1-M**	**E-1-L/C-1-L**	**E-2-M/C-2-M**	**E-2-L/C-2-L**	**E-2-M/E-1-M**	**E-2-L/E-1-L**
Metabolic pathways	331	242			372	284
Carbon metabolism	51	30	10	8	50	36
Fatty acid metabolism	26	17			26	18
Glycine, serine and threonine metabolism	24	17	5		30	20
Citrate cycle (TCA cycle)	22	11	48		18	
Biosynthesis of amino acids	37	22	9		43	24
Glyoxylate and dicarboxylate metabolism	15				16	11
Glycolysis/Gluconeogenesis	26	18				
Biosynthesis of unsaturated fatty acids	11	9				9
Steroid biosynthesis	10	13			13	
Terpenoid backbone biosynthesis	11	11	4			12
Selenocompound metabolism	8	9			9	8
2-Oxocarboxylic acid metabolism	10				13	
Fructose and mannose metabolism	18					
Cysteine and methionine metabolism	16	13	6		23	
Amino sugar and nucleotide sugar metabolism		21				21
N-Glycan biosynthesis		17				17
Glycerophospholipid metabolism		23				26
Arginine and proline metabolism		18			30	21
Starch and sucrose metabolism		13				14
Pyruvate metabolism		13			22	14
Pentose phosphate pathway		10				
One carbon pool by folate	9	7			11	7
Porphyrin and chlorophyll metabolism			7			12
Pyrimidine metabolism			9		47	
Propanoate metabolism					19	
Phenylalanine metabolism						7
Ubiquinone and other terpenoid-quinone biosynthesis						5
Valine, leucine and isoleucine degradation					22	
Protein processing in endoplasmic reticulum	53	41			67	56
DNA replication	20		6		31	13
Proteasome	22	19				22
Aminoacyl-tRNA biosynthesis		20	7	7		22
Ribosome biogenesis in eukaryotes			12	7	36	
RNA transport					68	
Mismatch repair					16	
Spliceosome					55	
Protein export					14	
Nucleotide excision repair					23	
Homologous recombination					15	
Base excision repair					16	
ECM-receptor interaction	33				36	
Cell cycle	47				58	33
Peroxisome						23

### Identification of significant DEGs between experimental and control groups

The more significant DEGs may play an important role in response to changes in the environment [38], therefore these were identified and annotated. The top five significant DEGs (up-regulated and down-regulated) are listed in Table 4. In fasting conditions, DEGs exhibited a log2FoldChange ≥5 for both up- and down-regulated genes, indicating a significant change in expression level. Specifically, calcium-binding and coiled-coil domain-containing protein 1 (*CALCOCO1*), kelch-like protein 38 (*KLH38*), methyltransferase-like protein 21C (*METTL21C*), F-box only protein 32 (*FBOX32*), SPRY domain-containing SOCS box protein 3 (*SPSB3*), hepcidin-1, nociceptin receptor, insulin-like growth factor-binding protein (*IGFBP*) 1 and 4, and krueppel-like factor 9 (*KLF-9*) were all up-regulated significantly. Type-4 ice-structuring protein (*AFP4*), major facilitator superfamily domain-containing protein 2A (*MFSD2A*) and genes involved in the metabolism of glucose and fatty acids such as group 3 secretory phospholipase A2 (*PLA2G3*), glucokinase, fatty acid binding protein 6 (*FABP6*), elongation of very long chain fatty acids protein-4 (*ELOVL-4*), apolipoprotein *A-I*, and long-chain-fatty-acid-CoA ligase 1 (*ACSL-1*) were all down-regulated significantly. Following re-feeding, changes in expression level were less pronounced than those under fasting conditions. However, it was apparent that all up-regulated DEGs were muscle-related (myosin heavy chain (*MYL*), myosin-*13*, parvalbumin beta, parvalbumin-*2*, myosin regulatory light chain 2 (*MYL2*), myosin light chain 3 (*MYL-3*), and troponin C). Genes such as lovastatin nonaketide synthase, kelch domain-containing protein 1 (*KLHDC-1*), ankyrin repeat domain-containing protein 29 (*ANKRD-29*), kyphoscoliosis peptidase, ankyrin repeat and SOCS box protein 2 (*ASB-2*), cytosolic phospholipase A2 gamma (*CPLA2*-γ), growth/differentiation factor 15 (*GDF-15*), ankyrin repeat domain- containing protein 37 (*ANKRD-37*), nuclear receptor coactivator 7 (*NCOA7*), and ferritin (middle subunit) were all down-regulated significantly.

**Table 4:** **Significant DEGs between experimental group and control group after fasting and re-feeding**

**Condition**	**Up or down**	**Tissue/comparison**	**Gene name**	**Log2Fold change**
Fasting	up	Muscle E-1-M/C-1-M	Calcium-binding and coiled-coil domain-containing protein 1 (*CALCOCO1*)	7.47
			Kelch-like protein 38 (*KLH38*)	6.67
			Methyltransferase-like protein 21C (*METTL21C*)	6.49
			F-box only protein 32 (*FBOX32*)	6.09
			SPRY domain-containing SOCS box protein 3 (*SPSB3*)	5.99
		Liver E-1-L/C-1-L	Hepcidin-1	8.39
			Nociceptin receptor	5.98
			Insulin-like growth factor-binding protein 1 (*IGFBP-1*)	5.73
			Insulin-like growth factor-binding protein 4 (*IGFBP-4*)	5.64
			Krueppel-like factor 9 (*KLF-9*)	5.60
	down	Muscle E-1-M/C-1-M	Group 3 secretory phospholipase A2 (*PLA2G3*)	−8.10
			Glucokinase	−7.10
			fatty acid binding protein 6 (*FABP6*)	−7.08
			Elongation of very long chain fatty acids protein-4 (*ELOVL-4*)	−5.59
			Apolipoprotein A-I	−5.27
		Liver E-1-L/C-1-L	Glucokinase	−8.15
			Type-4 ice-structuring protein (*AFP4*)	−7.31
			Long-chain-fatty-acid--CoA ligase 1 (*ACSL-1*)	−7.21
			Major facilitator superfamily domain-containing protein 2A (*MFSD2A*)	−7.14
			Group 3 secretory phospholipase A2 (*PLA2G3*)	−7.10
Re-feeding	up	Muscle E-2-M/C-2-M	Myosin heavy chain, fast skeletal muscle (*MYL*)	3.39
			Myosin heavy chain, fast skeletal muscle (*MYL*)	3.37
			Myosin heavy chain, fast skeletal muscle (*MYL*)	3.12
			Myosin-13	3.10
			Myosin heavy chain, fast skeletal muscle (*MYL*)	3.05
		Liver E-2-L/C-2-L	Parvalbumin beta	5.73
			Myosin light chain 3, skeletal muscle isoform (*MYL-3*)	5.43
			Troponin C, skeletal muscle	5.34
			Myosin regulatory light chain 2, skeletal muscle isoform (*MYL-2*)	5.27
			Parvalbumin-2	5.26
	down	Muscle E-2-M/C-2-M	Lovastatin nonaketide synthase	−2.53
			Kelch domain-containing protein 1 (*KLHDC-1*)	−1.97
			Ankyrin repeat domain-containing protein 29 (*ANKRD-29*)	−1.47
			Kyphoscoliosis peptidase	−1.41
			Ankyrin repeat and SOCS box protein 2 (*ASB-2*)	−1.38
		Liver E-2-L/C-2-L	Cytosolic phospholipase A2 gamma (*CPLA2*-γ)	−3.17
			Growth/differentiation factor 15 (*GDF-15*)	−2.99
			Ankyrin repeat domain-containing protein 37 (*ANKRD-37*)	−2.77
			Nuclear receptor coactivator 7 (*NCOA7*)	−2.70
			Ferritin, middle subunit	−2.63

### Identification of significant DGEs in experimental group between fasting and re-feeding conditions

To further investigate the mechanism of compensatory growth, significant DEGs in experimental group between re-feeding and fasting conditions were also identified and annotated. All listed DEGs showed a log2FoldChange ≥5, indicating a marked change in expression level between re-feeding and fasting (Table 5). *LA2G3*, Actin, *ELOVL-4*, glycine amidinotransferase (*GATM*), *MYL*, *AFP4,* elongation of very long chain fatty acids protein 6 (*ELOVL-6*), *ACSL-1*, and zinc finger FYVE domain-containing protein 9 (*ZFYVE9*)) were all up-regulated. *CALCOCO1*, alpha-2-HS-glycoprotein (*AHSG*), protein-glutamine gamma- glutamyltransferase (*TGM*), *SPSB3*, inactive dual specificity phosphatase 27 (*DUSP27*), calcium- independent phospholipase A2 (*iPLA2*), heme oxygenase (*HO*), solute carrier organic anion transporter family member 1C1 (*SLCO1C1*), solute carrier family 13 member 2 (*SLC13A2*, and suppressor of cytokine signaling 2 (*SOCS2*) were significantly down-regulated DEGs. These significant DEGs may play an important role in the response to re-feeding and fasting in teleost fish. The cDNA sequences (completely or partially) of significant DGEs could be available in Additional file 3.

**Table 5:** **Significant DEGs in experimental group between fasting and re-feeding**

**Condition**	**Up or down**	**Tissue/comparison**	**Gene name**	**Log2Fold change**
experimental group between fasting and re-feeding	up	MuscleE-2-M/E-1-M	Group 3 secretory phospholipase A2 (*PLA2G3*)	6.13
			Actin, alpha skeletal muscle 2 (*actin*)	5.85
			Elongation of very long chain fatty acids protein 4 (*ELOVL-4*)	5.68
			Glycine amidinotransferase, mitochondrial (*GATM*)	5.41
			Myosin heavy chain, fast skeletal muscle (*MYL*)	5.04
		Liver E-2-L/E-1-L	Type-4 ice-structuring protein (*AFP4*)	7.75
			Elongation of very long chain fatty acids protein 6 (*ELOVL-6*)	7.20
			Long-chain-fatty-acid--CoA ligase 1 (*ACSL-1*)	7.08
			Zinc finger FYVE domain-containing protein 9 (*ZFYVE9*)	7.01
			Group 3 secretory phospholipase A2 (*PLA2G3*)	6.95
	down	Muscle E-2-M/E-1-M	Calcium-binding and coiled-coil domain-containing protein 1 (*CALCOCO1*)	−7.82
			Alpha-2-HS-glycoprotein (*AHSG*)	−6.45
			Protein-glutamine gamma-glutamyltransferase (*TGM*)	−5.95
			SPRY domain-containing SOCS box protein 3 (*SPSB3*)	−5.73
			Inactive dual specificity phosphatase 27 (*DUSP27*)	−5.41
		Liver E-2-L/E-1-L	Calcium-independent phospholipase A2 (*iPLA2*)	−7.50
			Heme oxygenase (*HO*)	−6.60
			Solute carrier organic anion transporter family member 1C1 (*SLCO1C1*)	−6.32
			Solute carrier family 13 member 2 (*SLC13A2*)	−6.21
			Suppressor of cytokine signaling 2 (*SOCS2*)	−5.71

### Confirmation of DEGs by qPCR

**Figure 4:**
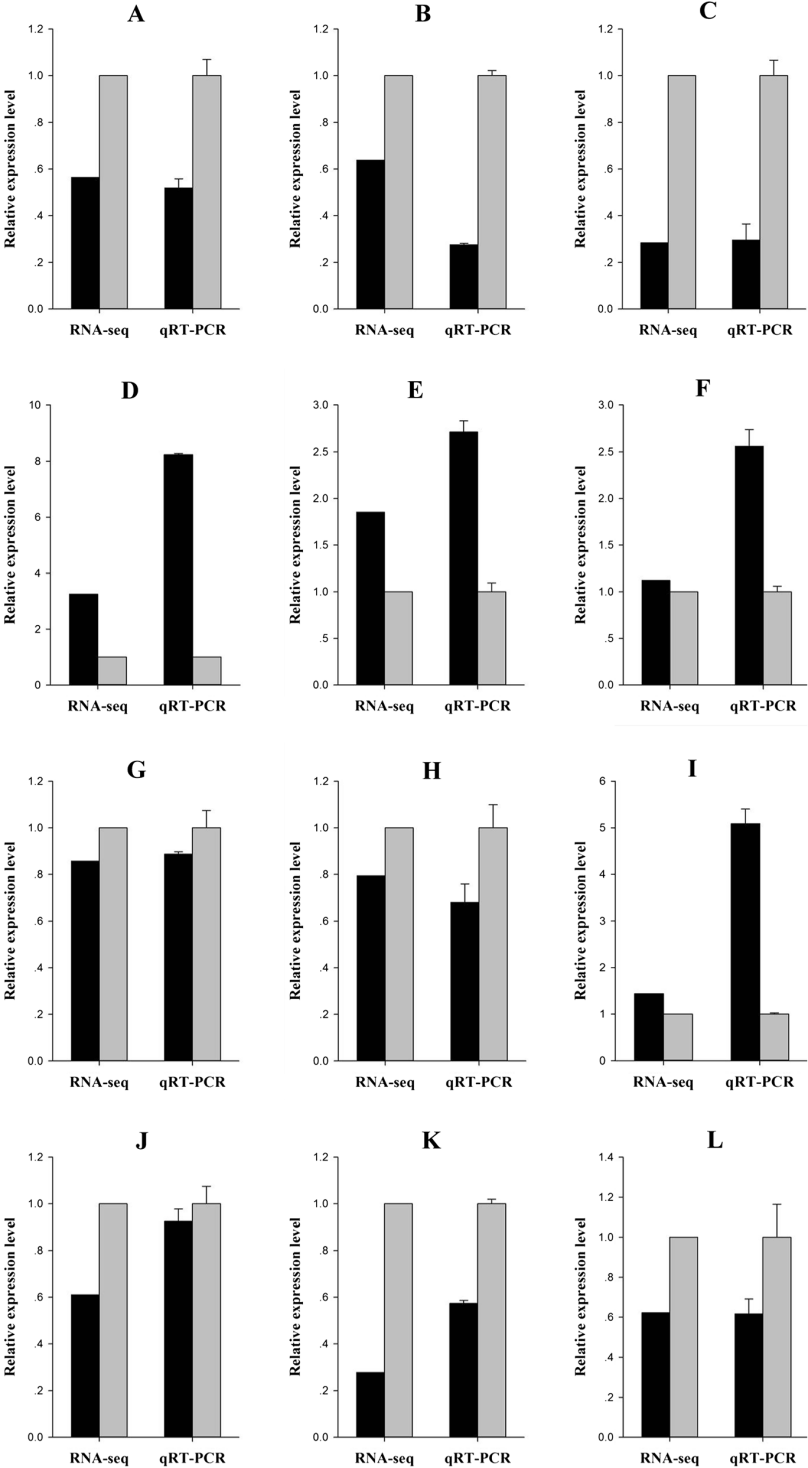
**Validation of DEGs by qPCR.** Twelve DEGs were random selected for qPCR analysis and compared with the equivalent RNA-seq data. The data from qPCR were presented as mean ± standard deviation of three replicates. The data from RNA-seq were the read counts that normalized by DEseq from three replicates. **A** and **B**, Expression level of *MIF* and *PRDX3* in comparison E-1-M/C-1-M (grey bars, C-1-M; black bars, E-1-M); **C** and **D**, *APOEb* and *EF-1*α in comparison E-1-L/C-1-L (grey bars, C-1-L; black bars, E-1-L); **E** and **F**, *APOA-I-1* and *PAIP2B* in comparison E-2-M/C-2-M (grey bars, C-2-M; black bars, E-2-M); **G** and **H**, *PSD2* and *ALDOb* in E-2-L/C-2-L (grey bars, C-2-L; black bars, E-2-L); **I** and **J**, *ALDOa* and *CFD* in E-2-M/E-1-M (grey bars, E-1-M; black bars, E-2-M); **K** and **L**, *EFF1*α*1L2* and *GAPDH* in E-2-L/E-1-L (grey bars, E-1-L; black bars, E-2-L).

To confirm the RNA-seq data, twelve DEGs were random selected for qPCR analysis. The RNA samples that form an independent repeated study and were used for reverse transcription and qPCR analysis. For each of paired-comparison, two genes were random selected. The random selected DEGs were macrophage migration inhibitory factor (*MIF*),peroxiredoxin 3 (*PRDX3*), apolipoprotein Eb (*APOEb*), elongation factor 1-alpha (*EF*-*1a*), apolipoprotein A-I-1 (*APOA*-*I*-*1*), poly (A) binding protein interacting protein 2B (*PAIP2B*), pleckstrin and Sec7 domain containing 2 (*PSD2*), fructose-bisphosphate aldolase b (*ALDOb*), fructose-bisphosphate aldolase a (*ALDOa*), complement factor D (*CFD*), eukaryotic translation elongation factor 1 alpha 1-like 2 (*EFF1a1L2*), glyceraldehyde-3-phosphate dehydrogenase (*GAPDH*). As shown in Figure 4, the expression patterns of all twelve DEGs that obtained by qPCR were similar to that in RNA-seq, although the relative expression level was not completely consistent. The results confirmed the reliability and accuracy of the RNA-seq data (Figure 4).

## Discussion

Due to changes in season, variation of temperature, unbalanced food availability, and other growth-stunting conditions, the growth and development of fish can often be restricted. However, when conditions returned to normal, fish can undergo obvious compensatory growth [39]. Despite numerous reports on compensatory growth in fish [40-42], the mechanism remains unknown, and global gene expression patterns resulting from compensatory growth are unclear. In order to reveal the mechanism and explore growth-related genes, a compensatory growth experiment was carried out on grass carp and global gene expression patterns were determined using an RNA-seq approach. As reported previously, compensatory growth can be classified into four types: over-compensation, full compensation, partial compensation, and no compensation [43]. In our study, the total increased weight of the experimental group was 1.26 g in five weeks, which was more than that in the control group. Moreover, the SGR of experimental group was significant higher than that in the control group during the week of re-feeding. A phenomenon of hyperphagia was observed in the experimental group during re-feeding (data not shown), which is a characteristic of compensatory growth. In addition, the RNA-seq data obtained in the study also implied that the compensation growth happened. Thus, although the initial body weight of fish in experimental group was slightly lower than that in control group, the results still demonstrated that at least partial compensation occurred.

Under fasting conditions, 4061 and 1988 DEGs were identified in muscle and liver, respectively, many of which were involved in metabolic processes, catalytic activity, binding functions, and participated in metabolic and biosynthetic pathways, according to GO and KEGG annotation results. Up- and down-regulation of metabolic and biosynthetic pathways may therefore be a strategy undertaken by grass carp in response to fasting conditions. Following re-feeding, 349 and 247 DEGs were identified in muscle and liver, respectively. Although less than that in under fasting conditions, most DEGs were up-regulated. These results suggest differences between the experimental group and control groups were reduced after re-feeding, but the differences that were apparent may contribute greatly to the fast increase in body weight observed. In addition, when samples from experimental groups in different conditions were compared, 4093 and 2444 DEGs were identified in muscle and liver, respectively, indicating extensive differences in gene expression between starved and re-feed fish. These DEGs may be particularly important for compensatory growth in grass carp.

The more significant DEGs that showed the largest changes in expression level were annotated. Under starvation, most of the significant down-regulated genes were involved in the metabolism of glucose and fatty acids (*PLA2G3*, Glucokinase, *FABP6*, *ELOVL*-*4*, *ELOVL-6*, Apolipoprotein *A-I*, and *ACSL-1*) [44-49]. *MFSD2A* and *AFP4* are known to be important for growth and development [50,51], and down-regulation of these genes indicates that metabolism of glucose and fatty acids is decreased sharply during fasting conditions, which prevented growth and was consistent with the observed reduced weight under these conditions. Of the significant up-regulated genes, *CALCOCO1*, *KLH38*, *FBOX32*, *SPSB3*, and *METTL21C* are associated with modification, ubiquitination, or degradation of proteins [52-56], whereas the nociceptin receptor plays an important role in response to stimulation [57]. Up-regulation of these genes suggests muscle proteins may be used as the major energy source to maintain basic metabolism in response to fasting, which may also contribute to the decreased weight. In addition, two insulin-like growth factor binding proteins, IGFBP-1 and 4, were down-regulated significantly. IGFBPs are reported to bind to insulin-like growth factors (IGFs), which prevents binding between IGFs and their cognate receptors, thereby inhibiting the activities of IGFs [9,12,58-61]. Thus, significant up-regulated of IGFBP-1 and 4 may be another reason for the decreased weight of fish under fasting conditions.

After re-feeding, the most significantly up-regulated genes were myosin-related, such as *MYL*, Myosin-*13*, *Parvalbumin beta*, *Parvalbumin-2, MYL-2*, *MYL-3*, *and* Troponin *C* [62-67]. This strongly indicates that biosynthesis of myosin or muscle occurred, which resulted in increased weight following re-feeding. Of the significantly down-regulated genes, Lovastatin nonaketide synthase is known to participate in the methylation of proteins [68]. *KLHDC-1*, *ANKRD-29, ANKRD-37,* and *ASB-2* are also related to the ubiquitination or degradation of proteins [69-71], and *Kyphoscoliosis peptidase* hydrolyzes muscle-specific proteins [72]. *GDF-15* is associated with appetite, and high expression level of GDF-15 may reduce appetite and weight in mice [73]. Ferritin (middle subunit) is a protein involved in response to cellular emergencies [74]. Down-regulation of these genes suggests that following re-feeding, fish exit from the emergency response, and the presence of food stimulates appetite. Subsequently, muscle proteins are no longer used as the major energy source, and rapid compensatory growth occurred.

Significant DEGs in experimental group between fasting and re-feeding conditions were also identified. *PLA2G3*, *ELOVL-4*, *ELOVL-6*, and *ACSL-1,* involved in the metabolism of glucose and fatty acids and identified above, were all significantly up-regulated. Actin, *GATM*, and *MYL* encode muscle-related proteins [75], and *AFP4* and *ZFYVE9* are important for growth and development [51,76]. Up-regulation of these genes indicates stimulation of glucose and fatty acid metabolism, and enhanced biosynthesis of muscle, which may explain the increased body weight. Of the significantly down-regulated genes, *CALCOCO1* and *SPSB3,* associated with the ubiquitination and degradation of proteins and identified above, were down-regulated. *AHSG* is able to make mice insensitive to insulin, and inhibits growth [77]. *DUSP27* encodes a protein that catalyzes the hydrolysis of amino acids [78], while *HO* is important in the response to cellular emergencies [79]. *SLCO1C1* encodes a receptor that mediates uptake of thyroid hormones [80] and *SOCS2* negatively regulates growth hormone and IGFs [81]. Down-regulation of these genes indicates that muscle proteins are no longer used as the major energy source following re-feeding. In addition, growth hormone and IGFs may be positively regulated, which may accelerate the growth and development of grass carp.

## Conclusions

In conclusion, grass carp were subjected to compensatory growth and global gene expression patterns were determined by RNA-seq. Numerous DEGs were identified and several significant DEGs were annotated. This study expands our understanding of the mechanism of compensatory growth, and will provide a reference for growth-related genes in grass carp.

## Availability of supporting data

The sequencing data in this study could be available form the Sequence Read Archive (SRA) at the National Center for Biotechnology Information (NCBI) (accession number: SRP055685). Other supporting data are included as additional files.

## Additional files

Additional file 1:
**The components of the feed that used in the study.**
Additional file 2:
**Sequences and efficiencies of primers that used in qPCR analysis.**
Additional file 3:
**The cDNA sequences (completely or partially) of DEGs that mentioned in the study.**
Additional file 4:
**Summary of RPKM level in different samples.**
Additional file 5:
**Detail information of DGEs in different comparisons.**
Additional file 6:
**Top 30 GO enriched terms in different comparisons.**

## Abbreviations

DEGs: Differentially expressed genes
RNA-seq: RNA-sequencing
RPKM: Reads per kilobase of exon model per million mapped reads
GO: Gene ontology
KEGG: Kyoto encyclopedia of genes and genomes
CALCOCO1: Calcium-binding and coiled-coil domain-containing protein 1
KLH38: Kelch-like protein 38
METTL21C: Methyltransferase-like protein 21C
FBOX32: F-box only protein 32
SPSB3: SPRY domain-containing SOCS box protein 3
IGFBP-1: Insulin-like growth factor-binding protein 1
IGFBP-4: Insulin-like growth factor-binding protein 4
KLF-9: Krueppel-like factor 9
PLA2G3: Group 3 secretory phospholipase A2
FABP6: Fatty acid binding protein 6
ELOVL-4: Elongation of very long chain fatty acids protein-4
MFSD2A: Major facilitator superfamily domain-containing protein 2A
MYL: Myosin heavy chain
MYL-2: Myosin regulatory light chain 2
MYL-3: Myosin light chain 3
KLHDC-1: Kelch domain-containing protein 1
ANKRD-29: Ankyrin repeat domain-containing protein 29, ASB-2, Ankyrin repeat and SOCS box protein 2
CPLA2-γ: Cytosolic phospholipase A2 gamma
GDF-15: Growth/differentiation factor 15
ANKRD-37: Ankyrin repeat domain-containing protein 37
NCOA7: Nuclear receptor coactivator 7
GATM: Glycine amidinotransferase
ELOVL-6: Elongation of very long chain fatty acids protein 6
ZFYVE9: Zinc finger FYVE domain-containing protein 9
AHSG: Alpha-2-HS-glycoprotein
TGM: Protein-glutamine gamma-glutamyltransferase, DUSP27, Inactive dual specificity phosphatase 27
iPLA2: Calcium-independent phospholipase A2
HO: Heme oxygenase
SLCO1C1: Solute carrier organic anion transporter family member 1C1
SLC13A2: Solute carrier family 13 member 2
SOCS2: Suppressor of cytokine signaling 2. MIF, macrophage migration inhibitory factor
PRDX3: Peroxiredoxin 3
APOEb: Apolipoprotein Eb
EF-1a: Elongation factor 1-alpha
APOA-I-1: Apolipoprotein A-I-1
PAIP2B: Poly (A) binding protein interacting protein 2B
PSD2: Pleckstrin and Sec7 domain containing 2
ALDOb: Fructose-bisphosphate aldolase b
ALDOa: Fructose-bisphosphate aldolase a
CFD: Complement factor D
EFF1α1L2: Eukaryotic translation elongation factor 1 alpha 1-like 2
GAPDH: Glyceraldehyde-3 -phosphate dehydrogenase
