# Supplementary material for: Global gene expression patterns of grass carp following compensatory growth
Source: BMC Genomics. 2015 Mar 14;16(1):184. doi: 10.1186/s12864-015-1427-2 (PMC4374334; doi:10.1186/s12864-015-1427-2)
Supplement: Additional file 1: — The components of the feed that used in the study. [file 12864_2015_1427_MOESM1_ESM.pdf]

Additional file 1 The components of the feed that used in the study

Components	percent
crude protein	≥30.0%
crude fiber	≤15.0%
crude ash	≤12.0%
crude fat	≥2.5%
total phosphorus	0.6~2.2%
Water	≤13.0%
lysine	≥1.3%
