# Supplementary material for: Global gene expression patterns of grass carp following compensatory growth
Source: BMC Genomics. 2015 Mar 14;16(1):184. doi: 10.1186/s12864-015-1427-2 (PMC4374334; doi:10.1186/s12864-015-1427-2)
Supplement: Additional file 2: — Sequences and efficiencies of primers that used in qPCR analysis. [file 12864_2015_1427_MOESM2_ESM.pdf]

Additional file 2 Sequences and efficiencies of primer that used in qPCR analysis

Gene name	Primer sequences	efficiencies
MIF	GTGGTGAACACAAATGTAGCAAAG	96.0%
	GTCAGGTGAAATGCCAAGGTG	
PRDX3	CCCAAGAAAGAGTGGAGGATTAG	101.0%
	CAACCGGCAGGTCATTTACAC	
APOEb	AAGGACCGTTTAGAGCCATACC	93.1%
	ATCTTCTCGGAATAGGGGGC	
EF-1α	AATCACCAAGGAGGTCAGCG	98.1%
	AATCTTCCATCCCTTGAACCAG	
APOA-I-1	AAGTCTGTGCTCCAGGTTTATGC	95.9%
	ATTGGGGAGATGGCTTGAAA	
PAIP2B	TGGAGCAGGAGTTTCTTGAGC	103.1%
	CTGACCGACAACCCGCTAAG	
PSD2	CACGACACCTGGGCAAGAATA	100.5%
	CTCTCCTGCGTCTCTCCCATC	
ALDOb	GAAGGGACAAGCAGCGAACA	99.6%
	CTGGCGGTAAAGAGGGACTG	
ALDOa	ATGGTAGTTGGCATCAAAGTGG	103.1%
	CATTTAGCAAAGTCTGCCCCA	
CFD	GGGCTCTTTGAACAACCTGG	100.8%
	ACCATCACAGGTGTCCTTTCG	
EFF1α1L2	CAAGGACATCCGTCGTGGTAA	102.9%
	GAAAACTTGCAGGCAATGTGAG	
GAPDH	TCACAGCCACACAGAAGACCG	95.0%
	TCAGGAATGACTTTGCCCACA	
β-actin	AGCCATCCTTCTTGGGTATG	99.2%
	GGTGGGGCGATGATCTTGAT	
