# Supplementary material for: Global gene expression patterns of grass carp following compensatory growth
Source: BMC Genomics. 2015 Mar 14;16(1):184. doi: 10.1186/s12864-015-1427-2 (PMC4374334; doi:10.1186/s12864-015-1427-2)
Supplement: Additional file 3: — The cDNA sequences (completely or partially) of DEGs that mentioned in the study. [file 12864_2015_1427_MOESM3_ESM.pdf]

Additional file 3 The cDNA sequences (completely or partially) of genes that mentioned in the study

>Lovastatin nonaketide synthase, CI01000358_00662699
TTCAATGAGTTTGACCACAAGTTCTTCGGCATCACTAATGCTGAAACAATCACCATGGACC
CTCAGCACAAACTTCTGTTGCAGTGCACCTACAGGGCACTTGAGGATGCTGGGATACCAAT
GGAAAAGGCCAGTGGAACGAGAACAGGAGTATTTATGGGCCTAATGAACAGAGACTTTGA
GCTGGCAAATGTGAGGATGAATCCAAAACACATAGACCACACCAATGCCACTGGTGCCGC
TATGAGTATAGCTGCCAACCGCATCTCATACGTATTCAACTTCACTGGCCCCTCAGTGTCCA
TTGACTGTGCCTGCTCCTCATCTCTCGTTGCCCTTCACTTAGCTTGTCAAGCCATAAAACAA
GGAGATTGTGATATGGCCTTGTGTGGTGGTGTGTCCTGTATATTGGAGCCGACCCTTTTTGT
GACTCTCTCCAAGGCAAAAATGATCTCCTCTGATGGAACGAGTAAACCTTTCAGCAGCAA
AGCAAACGGATATGGCAGAGGTGAAGGATGCGGGGTTGTTCTATTAAAGCCTCTGAAAAA
AGCTTTTGAAGACCATGATCACATTTGGGGCATCATCAGCAAAACTGCAGTAAATCAAGAT
GGCCACACCGTTAGTCCAATCACCAAACCATCCATGGTACAGCAGAAGGAGCTGCTTAGA
AAAATCTACTCAGCAGAGACTGACCTGACTAGTGTCCAGTACATTGAGGCTCATGGGACA
GGGACTCCAATTGGAGATCCAATAGAGGCAGGTAGCATCTCAAAAGTCATTGCCAAAGCA
AGACCTCAAAAGTCAGGGCCACTCATCATTGGTTCAGTTAAGAGCAATATTGGACACACA
GAATCTGCTGCAGGTGTTGCAGGGCTCATAAAGGTTCTCTTGATGATGAAGCATGAAACCA
TTGTGCCATCATTGTTCTACTCCATTGAAAACTCCAGCATAGACACTGAATCTCTCAATATG
AAAATCCCCACCACAGCAGAAAAATGGATCAGTAACTGCAGAGCAGGGAGGTGTGCAGG
AATCAATAACTTTGGGTTTGGTGGAACAAATGCACATGCAATTGTCAGACAATATGTGCAG
TCAAAAAAGCCAAAAGCTCAGTTGATAAGCAAATCCCATCAGTATTTTGTGGTCTCTGCAG
CCTCTGAAAAATCCATGAAAAATATTATTGAAGATACGGCAGAACAGATCAGCGCAGGGA
GAATATCAGATCTACAGTCTTTACTGTACACATCCGCATGTAGAAGAAGTCACTTGAAACA
CAAATACAGGAAAGTATTTCAAACATCATCATTGGCAGATCTGCAAGAGAAACTTACTGCT
GCTGTAAAGAAGAAGCTTGTACCTTCAAAGACAGACTCCAAGCTAGTTTTTGTGTTTTGTG
GCAATGGTGTTACATATCAGGGTATGTGCAAGCAGCTCCTAAAACAAGAGCCAGTTTTCAG
AGAGGAAATCTTGAAGATTGAAACTCTGTTGAAAAGCTATACAAGTTCGAATCTGATAGAG
ATGCTGGAAAGTGAATCTGAGAAAAGTACAGAGCTGTCTGATCCAAAAATTGTCCAGCCT
CTTCTGTTTGCTATTCAGGTTGCTGTTGTCAAACTTCTGAAACACTGGGGCATCAGGCCTG
ATGCTGTTTTGGGGCACTCTATTGGAGAAGTTGCTGCAGCTCATTGTTCTGGTTTGCTGTCA
CTTGAAGATGCAGTGAAAGTCATCCACTATCGCAGTTCTTTACAAAGCACTGTGACAGGA
GGAAAGATGCTGGTAGTCAGTAATATGGCTGTTTCTGAAGTCTTGAAAATTCTCCCATCTTT
TTCGGGAAGGGTTTGTCTGGCTGCTTATAACAGTCCTCAGTCATGCACCCTCTCAGGAGAT
GCAGATGACATTGACAGGTTGCAGAAGAATTTGAGCAACTCAGCCAGTGGAAAAGATTTG
TTCCTTCGCATTTTAGATGTACCTGCAGCATACCACAGTCACAAGATGGATCCCATTTTATC
CAAAGTGAAACAGAGTATAGGCTCATTGCAGGCACATAATTTAGAGACAGAATTATTCTCA
ACAGTGACAGGTGTTAGTGTGTGTTCCTCAGATTTTATTACTGGTGAATATTGGGCAAGGA
ATATCAGAGAACCTGTTGAATTTGAACAAGCTGTACAGTCTGCAGCTGAAAACAAAAGGA
GTGCTATATTTGTTGAAATTGGTCCAAGAAGATCTTTGCAGAGGTACATCAATGAGACTCTA
GGAAATGAATTCACTGTGATTCCCTCAGTGCAGCCTGATAAAGATCAGGAGACAATTCTTG
CAGCTGTTTCCAAACTGTTTGAGCTTGGGTTCAAAGTAGATTGGGAAACATTCTACAAAGG
TTTTGAGACTGAACCAATTCCCTACCCACAATACCAGTTTGATGATGTGAAGAGAGATGCC
TTTGCTTCAAATTTGCAATCGATCGGTCCCACTGGCAACCATCCAGTAGTTACACAAATGG
GTACAGACAGTTCAATGTTCAGCTGTGATATATCCTCTGATTCAATGGCCTTCCTGCAGGAC
CACAAGAACAATGGGGTTGCCATCATTCCTGGGGCATTTTATGCAGAGTTGGGTTTAGCAT
CTTACATGGCATATGCAAAACCTAAAGTTCCACTAAGTTCTCTGCAGCTCAGCATAACATTC
CAGAGTCCATTTGTTTTCACGCAGAATGCCCCAGAAATAAAAGTACAGCTGGATCATTCGG
ACTATTTGGCTGATAACTCATGTAACTTCAAAATACAGTCTACTTCTGCAGTTTATGCATTTG
GCACAGTAGAAGCAAAACCAGGTAGAACTCCTGAAGAACAGTCAATTTCATTAGACTGTA
TTTCCAAAAGATGCACATTCCACATGAGTACTGAAGAACTCTACAAACTTCTAAGTCAAGC
AGGATTTGAGTATGGGTCTGTCTTCATAAATAATGCAGATGTTTATTATGGGGAAGAATTTA

GAGAGGCTATATCTGTTGTGAAGGTCCCAAAGGAAATACTGCCTCAATTACATGACTATCA
CCTTCACCCTGTGCTCTTGGATTACGTCATGCAACTTGTTCTTGCAACTATAGGAGGTGTAT
CAACAAGGCCCCAATATCCTGTGCAAATAGGAAGCCTGACTGTATTTGAATCATTGCAAGA
GGAGATGGTTGTATATCTGAGAGCAGTACATGTAGGAGAAGATGATTTTGACATTTGTGGC
TGCTTAGCCAACAAACAGGGTAGGGTGTTAGTCGAACTGAAGCATGTGAAGATTAGAATG
CTAGGAAGTCGTCCCCAGGTAGTCAAGGAATACTTCTTCCACAACGATTTCAGTATCATCT
CTGAAGTTGCCACGTTTGATACACAGATTAAGGCACTGGTCTTTTCTGGCCTGAAAGGAAT
TTGTAAAGCTTTGCAACAGTACTTGGACCCAACATCTAGATATGTGTCTTCTTCAAACAGTA
AAATACTGTTAGAAAATGGAGTTGAATTTCTTTTGTCAAAACTGAAAATTTCAAGTGTAAA
AAAAAACTTCCAGGAAATTTTGTTCATGTGGGGTGATGCAGACCTAACCTCCCTCAAATCA
GAGAAGGTCTTGGACTGTATGGTGGGGTGTTGTGAGATTTTCCGAAAGCTTGTCAGATATC
TCAAGGCACTTAATTTCCCAGGTGATTTCAGAGTAATAACCTATAGGTGCTCTGAGAGCTTA
GTGGACCACATAAACCCTGGGTTTGTTCTGTCAGGCATGACAAGAGCATGTGCAGCTGAAT
TGTCAGAACTTTCCTTCCAGCTGATTGACATGGGCTCTGCCACTTTTGAAGACATCAAAGC
TTTGGTTCAGGTCCTCAGATCATACCCCTGTAACAAATATCCAGAGCTAGTAGTAAAGGAG
GGCAAAATTCTCAAACCTGAAATCACCCACACACCTTTGCCAACCATGGCAATCTCTTCAA
GAAATGTTCACATGTTGGATGAAGAAGTCTTCATGTTGCAAACATCTGACCCATATATAATG
ACAAATGTGTCCGCGACTCAAATGGATGATTCCATTGAACTGATTCAAGGAAAAAAATATTG
AGCTCCATCTCAGTAAAATCTGTGTTCATTCATCAGATTACTTTCCAGTCAGCATTTCTGAC
CTGAATTATGGTCGGACATTGTACTGGAACAAGCACACAACTGAAAACCATAAGCTCTTAG
CCCTTGACTTCAGTGGCACTGTCACAGCTGTGGGTAAAGATGTCAGCAAATTTAAAGTTGG
TGATCATGTTGTGTCTTGTTACCCTGTTGCTGCCACCACTAAAGTAGTTCTCCCAGCAGCTG
TTTGCTGCAAAGCTAAAAGGCTTTCATTCTTGAATGACATGCCTTGTGTCTCTTATATGGTA
CTGGCTTGGGAGATCTTGCATGAGGCTTTACCCAAAGCTAAACAGCAATGGAAGTTGGGC
ATTTTCTCCACTGTTCCTGACTCAGCTTTGATGAATGTCTTAATTGCTATTGCAAACAGATC
AGGTTGGAATGTCAGAGTGAGCACGCAGGCTGATCAGCTGTCTTGTGATTTTAGTGAGGTT
GTGGGAGCTGTTCTTCTACCTCCTTATAATGTAGAAACCGCTAAAATAGCTAGCAGTGTTAG
AGGCATCAAAGACATTGTTCTTGTCTATGACAACCAGACAGAATTTCCATTAAATACTACA
ACATTCCGTGGTACAAATGAGGAAGTCCACTTCCATGTCCTCCTCATGACCCAGATAATGC
AAAAAGGGTCTCTGCAAATGCAAATGCCACACATCTACCGTTGGCTGAAATCCATGCATTT
GGACAGAAAGTCTTTGTCTTTGGATGCAACAGCTATTCAAAGAGTGAAATTTGAAAAAGA
CATTGGTCTTCTCTCCGTAAAAGAATCTGAATCATACTTCAGATGCCAGATCCTACCCATTAT
CATCATGAACAGTGAAAACAAGAACCAGCTGTCTGACATTCCAGTGATGCCCAAACACAG
TCTATTCAAGACAAATTCAGTCTATATTGTCACAGGTGGTCTAACAGGGTTGGGCTTTGAG
ACAGTAAAGTTCATTGCTCAGAAAGGAGGAAGAAACATTGTCATTCTCTCTAGAAGTAATC
CAAACACTCAGATGCAACAAGAGATAAATCAGGTCAGTAGCCGTTGGGATTCTGTCATTAA
GTGTCTGCCGTGTGATGTTTCTGTATCTGAGCAAGTGGACCAGACAATTGTTAATATTGGA
AAACTTTTCCCATCCAGTCCAATCAAAGGGGTATTTCACAGCGCAGTGGTCCTGCATGATG
GGCTGATTGAACGTCTTGACAAATCTCTTTATGAGAAAGTTATGAGGCCAAAAGTAAATGG
AGTGATTAACCTTCACCGTGCTACCATACAGTGCAATCTGGATTATTTTGTGTGCTATTCCTC
CATTGCTGCCTTCATTGGCAATGCCTCACAAAGTAATTATGCTGCGGCTAATACATTTATGG
ACACTTTCTGTCAGTACCGCAGAAACATTGGGCTTTCTGGACAGTCCATTAATTGGGGGGC
TTTGAATCTTGGTCTTTTGTTGAACAAAGTCCATGTCCAAAGATTTCTGGAGGCAAGAGGA
ATCTTGCTTATGGAGATTCCAGAAATCCATGATAGTCTTGAGCAGTGCCTCTTGATCAACAA
ACCTCAACAGGTTGTATGCAAATTCAATTTTAAAAACAACTGGAATAACATTCTCAGTCAG
AACAAAACACTGAATGTGCGCTTGTTCAAAATAGCACAGGAAGCTATAAGTAAAGTTGGA
GTGAATTTATCTAGACCTGAACAACCCAAGAAATCATCCTCACCACGTGATTATCTCTGTCT
CATGATTAGTGAAACGACTGGTATTGAGATGGATGAGCTGAATGATGATGTTCATCTTTTCG
ATTTAGGCATTGACTCAATGTTAGCTATGACTCTGCAGAATCGTATCTTCAATGATACAGGT
GTGACTGTCCCTCTAGTGACTCTGATGGATCCAAATAGCACACTGACAACTCTCATTAGAA
TCCTGGAGAAGAACTGTGTAGATGGATATCAGAGTGAAGATGAGAGCAAATCCACATACTT
ATGA

>Group 3 secretory phospholipase A2, CI01000168_01115503
ATGCAAAGCAGCCACCTTTTAAAGGCAGTTCTGTGCCTTTTCATGCTGTTTCATGCTTGTTT
CAGTGCTGACAATTTTAAATCGAGTACTTACTGTATTTGGACCAGATCCACTTCAGGTGGG
CAAACACAGTATGCATTTCTCCGGAGAACTCAGACATCTCTTCTCTTGTTTGACAGCATCT
GGAACAAAAACAACAGCCTTTTTAGTTGCATAACAAGTGATGAGCAATCTGTTATTGAGAG
TTACCTGTCCAGGTGTTGGGAAGACAGCTCTTTCTCAGAGACCCTTGATGCACGCTTTGAC
ATCAGTCAGCTGATTGAACCTGATGGCCCCTGTGAGGCTGCTGGTATTACGGAGGAATTTA
CAGCATCCGTACGAAGGGCCAGAGACCTAAAGAGTGTTAAACCCGATGGATCCATGTACC
AGGATAGTGGAGAAAGTTCCAGACAGACACTTCACCGATCAAAGCGCTCGTGGATGATTC
CAGGGACTCTGTGGTGTGGCTCGGGAAATAAGGCTACTGGCTTTACTGACCTGGGTATCTT
TGAGGAAACCGATAAATGCTGCAGAGAACATGACCACTGTAAAGACACCATCGACTCTTT
TTCATACAACTATGGTGTCTTCAACACAAATATCTTCACTTTATCACACTGCGATTGTGACA
ACAGGTTCCGCCGCTGTCTCCTGGGTGTTAATAATACCATGTCCAACCTTGTGGGCTACGG
ATACTTCAACGTGCTAAAGATGCGCTGCTTCGAGTTTTCTCAACGGATGCAGTGTGCGAAG
AGAACGTGGTGTGTAAATTCAGAACTTGCTCAATACGCCGTGGTCAAAGACGCAGCAAAC
TACACCGACACGCTCCCTGAACAAGACATGGAGACTCTGGAAATGAGTTTCCATCAAGCA
ATCACTTTAGGACAAAACCAGGTCACCAAAAACATGGAAGAATCGCTTATCATGCAATCCA
AAGATTCCAAGAGCAGCACTGAAGAGCCGAGTCCAGTTTCTACATCATCACCATGGCCATC
TGTTACAACGCTGCTGTCCCGGAAATCTGAAGCTTCCGTAGTCACAACATACGCACCAAA
GACAACAATCCCCCAAAATAAACCAGACAGACAACATCAAGGCAAGTTTGAGATGTGTGG
CCTCTACAGGGATCTGGATTCATGCCATCTACAGATCCCTGCTTTACAAGAGAAATTCGGTT
TACGAAACCCTGACCTGAGAACCCTTTATCACTGTAACTGTACTGCCAGGCTCGTCCAAAA
GATCTTGTCGAAAGAACTGGATGAAACTGACCCTGTGCATTTGCTGTTGATGGACTTTGTG
TCACAATCCTGTTTCACCCTACCACAGACTGAGAACTGTCTCAGTGGAAAAAGTTGCTCA
ACCCATCCACCAGAAGCACAGCTCATCCAACACTGGAGGAAAGACGTGGCAAGTGGACG
ACATCTAGTGGACTTCAAGCGCAAACTCAAGAGGATGAACCTCAAGCGCTCTAAGAGAAA
AGACTCTCCAGTCAGATTATATAAGAAATGCATCAGGATGCACACTAAACGGCAGAGACCT
CGAGTTCCTGAACGGAAACTGTCATAA

>Alpha-2-HS-glycoprotein, CI01000004_02018248
ATGGCTGTTCTTGGGCTCCTGGTAATGGGCTCATGGGCACAGGGAGTGATGCCCACCTTCA
GCTTGCCTCCCTGTGATTCCCCGGAGGTTGAAGCAGCGGCTCTGGTGGCACAGGATTTCCT
TAACGCTCAGCACACCCACGGTTACAAATATGCCCTGAATCAGATTGACAAAGTCAAGATC
CTCTCCAGGCCAGGTCATGCCGACACATATCTTCTGGAGTTGGACTTGCTGGAAACTACAT
GTCACGTCTTGGATAAAACACCAGTGAGCCTGTGTCCAGTCAGACAAAAGCATAGTACGG
CTGTTGAGGCAGATTGTGATTTTGCTCTGACAAATACAACTCAAGGTCTGTCTGTTGTGGC
ATTCAAGTGTAAAACCGAAACAGAAACAGAGGACTACTGCGTAGGCTGCCTTCACCTCGT
TCCTTTAAATGACACAGATGGCCTCCAACTCATTGAAAGCTCTTTAGATAGTTTCAATAAAA
ACAATTCTCTAAACACTAAGTTTGCACTCCTTGAAATAGGACGAATGGCATCCCAGATTGT
CAGCGGCGGACACAAGTACTTTGCAGAATATGCCATTATTGGGACCAACTGCACAAGTCA
AGATGATGATATATGCATCCCTCCAGATTACACTGTTGCTAGCCAAGCTGTGGACCCAGCAA
GCAACGCCACAGTGCAAGTCCAACAACTTCTGCATGCACACACCTTTGGACCTCACCACA
ACCCCACCATTCATGGCCTCAGACATCACAAGCTAACAGCACTTCATGACCCTGCGGCAA
GCGGCCTGATCTCTGCAGAATCTGTTGAATCAGCTGAGGTAGTGGTTGTGCCAAAGGAAG
CTCCTGTTGTAAAGAGGGAGGTTTCTGCACCAGAAGTAGCAGCAGTAGATGCAGCAGTAG
ATGCAACAGTAGTGCCAGAAAAAACATCCATTCTTTCAGACCCAATTCTTCTTGTCCCAAT
CTGTCCAGGAAAAAAGAAACATTTCTAA

>Myosin light chain 3, skeletal muscle isoform, CI01000299_04126314
GCTGGAGAATTCTCTGCTGACCAGATTGAGGACTTCAAAGAGGCTTTCGGTCTCTTCGAC
AGAGTTGGTGACAACAAGGTTGCCTACAACCAGATTGCCGACATCATGCGCGCCCTGGGT
CAGAACCCCACCAACAAGGATGTGAAGAAAATCCTGGGTGACCCATCTGCTGACGATATG
GCCAACAAAAGAATTGACTTTGAGGCTTTCCTGCCAATGTTGAAGACTGTTGACGCCGTC

CAGAAGGGTACCTATGATGACTACGTTGAGGGTCTGCGCGTCTTCGACAAAGAGGGCAAC
GGCACAGTGATGGGCGCTGAGCTGCGTATTGTGCTGTCAACACTGGGTGAGAAGATGACC
GAGCCCGAGATTGACTCTCTCATGCAGGGACAGGAGGACGAGAACGGCAGTGTCCACTAT
GAGGAAGCCACCTCAGGCCTTGTATGGCGAGAATCTTTCCTTAAGCATGTCTGCTTCTCCT
CTTACAGCTTTCGTCAAGCACATCATGTCCGTGTAAGAGGCCGTCCGTTGAGGGAGTGGTG
A

>Inactive dual specificity phosphatase 27, CI01000299_06359190
ATGATTCTTATCGTGGTGTCAGTCGATGATAACAGTGTGATGGCGTCGTCCGGTGAGAGCA
GTGCTGCGGAGGATCAGCAGGTCCCACATCAGGATGAAGCAGCGAGTGTTAAAGACGTTC
AGTCGCATTACTTGCGCTGTCCTTCGCCCAGCTTCTCCATGATGTCCGATCGCTTCTCCACC
ATTTCGGACCGTTTCTCCATGATCTCGGGCTCAGAAGCAGAAAGTATCTTCATGGACCCGA
TTCATCTGTCATCCTCCATCGCTGCCAAGAAGATCATCAGTGAAGAGCTGAAGTCCAGAGA
CGTCAGGACGCCGTCGACCCCTGACAGCATGCTGGAATCCGCCGAGCAGCTGATGGTGGA
GGATCTGTACAACCGGGTGAAGGACATGATCGACGACCGCAGCCCCTACAACACGCCGTG
TGTGCTGGACATCCAGAGAGCGCTGATCCACGACCGGCTGGAGGCTCCGTTCAACCCCGT
GGATGAAGTCTGGCCCAACATCTTCATCGCAGAGAAGTCCGTGGCTGTGAACAAAGCCCG
TCTCAAACGCCTCGGCATCACTCATATTGTGAACGCCGCTCACGGCACCGGCGTCTACACG
GGCGAGCTGTTCTACCAGGGCATGAACATCACCTATATGGGCATCGAGGTGGATGATTTCC
CAGATGCGGACATCTCTCCTTACTTCCGCACGTGTGCTGAGTTTCTGGATGACGCTTTGCT
GACACACAGGGGCAAGGTGCTTGTGGACTCGATGATGGGCGTGAGCCGGTCAGCCGTGCT
TGTCGCTGCTTACCTCATGATCTTCCAGAACATGAGCATCATGGAGGCCCTGCTGGAGATC
AGGAAGAAACGTGCCATCAACCCCAATGAAGGTTTCATAAAGCAACTGCGGCAACTCAAC
GAGACCCTAATGGAGGAGCGAGATGAAGACGATGACGACACCCTCAGTCAGTGCTCTGTG
ATTGACACCCGTGCTCGTCTCGATGAGGAGGAGAGCATGTTTGGAGTTAAAGCCGACTCTA
TTATGGTGGATGAGGAGGAAGACGGCGGCAGCGTCATGAGCAGTGTGGCTTCTTCTGCTG
CTGCCGCTGCGCTGAGAGCAGGACTTCTTGGCGGCCCAAACAAACCCGAGCTGGAGGTG
ACCGCAAAGGACCCGACGCTCCCTGGAAAAGCCAGGGATGATGAAGACGGGGATGTGGA
CAGCATGATAAGGGAGTGGCAGAAGCGCAACGAGAAGTACCAAAATGAGGACTGGTGGG
AGGCGCAGCTACTGTGCGACGGGGAGGATGGCGAGTCTTTGCTGGGCGAGAGGAAGCAG
CCAGCCGTGCGACCGGAGGACCTGGAGAGCGTGACCAGCGAAGATGTACAAATGGTGAA
GGAACGCATCAGACGTCGCCCCCGTCGACCCGCTTCAGATTCTGGATCCACAACCAGCTG
CAGCAGTTATGCCGATCTCTGGAAGCAGCGTCTGAAGGAGATCGAGGAGCAGGCCGCTGC
ACGATACCGACTCAAAGAGGCTGACGAGGACACTAACAGCGAAGCAAGCCAGAAGAAAA
TCGACGATGATGTCGAGAGTATACTCTCAGACAGCAGCTCCATGTACAACTTCTGCAAAAA
GAACAAAGAAAATCTAACACCTCTGGAGAGGTGGAAGATTAAGCGAATTCAGTTCGGATG
GAACAAAAAAGACGAGAACGGAGAGCAGACGGAGGCTGAGACGGAAACGCCCGCTCCC
TCATTGGAGGACGTCAACTTAACGGCCTATCAGACCTGGAAACTGAAGCAGCAGAAGAAG
CATGGCGGTGAAGAGAACAAGGACGAAATTCTGGAAATGAGTCGAGCAGAGGACACGGC
CACGATCAAGAGAAGACAGCGAAGAGAGGAGCTTCTGGAGCGCACACGGAAAACACTC
GAGGAGAGCCAGTCGGTGTGCGGCTGGGAGACCGAGAGCGCTCTGAGCGGCGGGAGCA
GCATCCCGCTGTCTGCTTTCTGTGCAGGAGCCTTCCCGTCTGCAAGCGTTGCCGGAGACGA
TAACATGTCTGTGCTGAGCGGAAGGTCCTCTGTCCTATCAGGGCGAAGCACACGATCCCA
GCCTCCTGTTCCCCAAGAGCCTCCTACACCACCCACTCCGGTCATCGGACCTAACGGCGA
GCCCATGGTAAACCTTGCCAATATCCAAAACTGGATTGCCAACGTGGTCAATGAGACTCTC
ATGCAGAAGCAAACAGAGATGATGATGGGAGCGAGTCTCGCCCCTTCCAGAGCGGGATCA
GTGTTTAGTCTGGGTGCTGGAGTCTCAGCACGTGGCGTAGACGATGATAAAGTCTCAGTGT
TGAGTGGAGCCACGTCCTCAAGCGTACTGTCCCGAAGTAGAGCCGAGTCCGTCCGCTCTG
TGCTGTCGGCTGGCGGTCGCGCCGAATCCGTGCTTTCTGCTGGAGGAGCATCCAACCTCTC
CTCGGTGTCCGGTTTGGGCTCACGGAGAAGCAAGATTACTACAACCAGTGTGCCGCTGTA
CAGCCTCTTCCAAGACCAGGTGAACCTTCACAAACTGGACACCATGGAGAAGGAAATCA
AGTCTGACATGCGGGACAAGATGGCGTCTTATGAGGTGAAGAAGATTGCAGAGGACAACA
AGCGCAGCACGTTATACAAGAAGAAGAAAACAAAGGAGGAGGACGATGAGGATGAGGCC

GGTCTTGGAAAGTCCAATGGATTTGAGGATTTGGCAGCCCGTTCCTCTGAGAAACCCAAA
CCCAAAAGAGATTACGGCCGTTCAGGAATACTGAACCTTCCGGCTTCAGCCAGTAACCCC
ACCAGCAGCATTGATGAATGGCTCAAAAATGTAAGACCTCCTCCGAACAAACCAAAACCG
AATGATGGAGACGTCGAGCCTCTCCGCATGACTCGGCCATCCGGGATCGAGGAACCCTCG
GAGTTCGACTTTCCGAGCCGCAGGAGCTCAATTTCAGTGGATGAAGAAGCAGAAGAGGA
GGAATATAGCTTTGCCTCAAGATTTTCATCCAGGCATCTGGCTGATGCTGACATGGATTTGG
ATCGAGATCCCAGTCCAGAGTTTAACTATCGATCTCGGAGGTCCCCGCCTTCTGTTGAGCC
GTCGTATAACGGCTTAAGTGAAGCTACTACTTACAGAACTAGGAGATCTTATGCAAGATATG
AGGAGGAGGAGGAGAGGGGCAGTTATCAGAGCTACTCAACAAAGCAGAAATTACATGAG
AACACTGAGACTGTGAGGACGGCTAAAACTGGACAGGAAGACGAGGAAGATGATGAAAT
CTCTGCTTTCATTGCTCAGATAAAGCAGAGGGCGAGAGCGCGGTTGGCCGAAGAAATGGA
GGATGATGAAGTTCTTTCCGCATGGAGGAAACAGGAGGAGTCAAAATCACACGTTCACAA
AAACTAA

>Actin, alpha skeletal muscle 2, CI01000302_01067213
CACAAACACCCAGTGCTGGCCTCTGTGTCCATTCGTTTGGTGGCGCTGGACTCTCCTTACA
CGGCCCTGCACCCGCCCGCTGTGGTTTGGGGTCTAGAGCGCCATATATGGACCGTCCCGCG
CGCCCGGAGCGCGGCGCTGTGCAGCTCACAGGCGTTGAGACCCTCCGCTCGACCCAACG
ACACCCAAATATGGACGAGGATCGGGAAGAATTCCATTCGAAAGCATGGACATTCCTTAAT
GGTTGTTCGAAGAGCTGAAAAAGCAGAGGACGCCGGGATGTGTGACGACGACGAGACTA
CAGCTTTGGTGTGCGACAATGGCTCTGGCCTGGTCAAGGCTGGTTTTGCCGGTGATGACG
CCCCCAGGGCTGTCTTCCCTTCCATCGTCGGCCGCCCACGTCACCAGGGTGTGATGGTCGG
TATGGGTCAGAAAGACTCCTACGTTGGAGACGAGGCTCAGAGCAAGAGAGGTATCCTGAC
CCTTAAGTACCCAATCGAGCACGGCATCATCACCAACTGGGACGACATGGAGAAGATCTG
GCACCACACCTTCTACAACGAGCTCCGCGTGGCCCCTGAGGAGCACCCAACCCTGCTCAC
TGAGGCCCCTCTGAACCCCAAGGCTAACCGTGAGAAGATGACCCAGATCATGTTTGAGAC
CTTCAACGTCCCCGCCATGTACGTGGCCATCCAGGCCGTCCTGTCCCTGTACGCTTCTGGC
CGTACCACCGGTATTGTGCTGGACTCCGGTGATGGTGTGACCCACAATGTGCCCATCTATG
AGGGTTACGCTCTTCCCCACGCCATCATGCGTTTGGACTTGGCTGGTCGTGATCTGACAGA
CTACCTGATGAAGATCCTGACTGAACGTGGATACTCTTTCGTCACAACCGCTGAGCGTGAG
ATTGTCCGTGACATCAAGGAGAAGCTGTGCTATGTTGCCCTGGACTTTGAGAATGAGATGG
CCACCGCCGCCTCTTCTTCCTCCCTCGAGAAGAGCTACGAGCTTCCCGACGGTCAGGTCAT
CACCATCGGTAACGAGCGTTTCCGTTGCCCTGAGACCCTGTTCCAGCCTTCCTTCATTGGTA
TGGAGTCTGCTGGCATTCACGAGACCGCTTACAACAGCATCATGAAGTGCGACATTGACAT
CAGGAAGGACCTGTATGCCAACAACGTCCTGTCTGGTGGTACCACAATGTACCCTGGTATT
GCTGACCGTATGCAGAAGGAGATCACAGCCCTGGCTCCCAGCACAATGAAGATCAAGATC
ATTGCTCCTCCCGAGCGCAAGTACTCCGTCTGGATCGGCGGTTCCATCCTGGCATCCCTGT
CCACTTTCCAGCAGATGTGGATCACCAAGCAGGAATACGATGAGGCCGGCCCCAGCATCG
TCCACCGCAAGTGCTTCTAA

>Kyphoscoliosis peptidase, CI01180000_04335433
ATGGATGAAGCTCAAGCTGATCAGCAACCAATCACAACAAAAAACAGCAACCGAGGCCA
CCAACCAGAAGAAACTGCCACAGACTCAAACAATGACACACAGAAACAAAACTTCGATT
TGAAAGTTGAAGATGGTGTGGTGAAATACAAACTTCAAAATGTATCTGGAAGCCAAGAGA
CCGGCATGAGGGATAAGAACAGCACCAAAGTCTGTTTGACTATTAAGAGCCACCGAGGAA
GTTCACAGTCAGTGGAGCAAAATGACCCAAAGCCCTTAACAATTGTACCGATACAAGAAA
ATGCTGTTTCACATAAGGTACTGTTGGAGAACTATGAAGGGAAGTTATGTGAGGAACAACG
GCCGAGCACAAAGCGCCAACTGTCCGCCGAGAGCGCGGCGAAGTCAGTGAGCGTAAGAA
AGAGGAGCACTGTGAAAAAGAGCCAAGAGGAGATTGTGAAACCCCTCTCAGTTGCCAGC
CCTGGCACGCAATCTGGGAGACCTTATCTACCTGCAGTCTCCTCCTCTCAGAGGAAACCCA
GAAAATTGCTGTTCCCCAGCACTGATATTTTCAACAAAGTAGACACATTCTCAATCAAGAA
AGGAAAAGAGCTGAGGGAGCTGGAAGTATTTGATGCTCAGAAGATTGCGTGTGCAGTCAC
TGAGGGATGCAGAAATGATCTGGAAAAACTCAGGGCCATATGGATCTGGCTATGCCATAAT

ATTGGCAAATTGTCTCATGCGTTTGTGTACGTAGAGTATGATTTAGAGGGATACCTTGGTCT
TTCTCAGAAGATTTGCTCATCTGATGAGGTCATCAGGCAGGGAAAGGGTGTATGTAGTGGA
TACTCCGGTCTCTGTGTGGAAATGTGCAGGGAGGTGGGTATTGAGTGTGTGGAGGTGAGT
GGGTACAGCAAAGGCGTTGGATACCAGGCAAGGCACAGTTTAGCTGAGAAGCGTTCCGAT
CATGAGTGGAATGCTGTCTTCGTGGGGGGTCAGTGGTGGCTGCTTGATGCTTGCTGGGGC
GCTGGCACTGTGGATATGAAAAGCAAAACATTTGTGAAGAGGTATGATGACTTTTATTTCC
TGACTGAACCCAGTGAGTTCATAAACTCACATTTCCCTGATGACCAGACCTGGCAACTACT
CACCACACCTATCTCTATGGAAGAGTTTGAAATGAGACCCTTGAAGACCTCAGCATTTTAT
CAGTTTGGATTGACTCTCACACAACCTACACAATATAAAATAATCACAGATGACGGGGAAG
CAGTTGTGTCTGTGAGATCCTCAAGACCATTGACCTTTTCGTATGAGATGAGGCAGCAGGA
TCCCCAGACAGGAGCTCTAAAGCAGGAGGAGGTTGACAGCTCCTGTGGTCTCCTGTCAGT
TACTCAACAAGGGATGAAGCTGCGATTACTGCCCCCAGAGCCTGGGGCCTATGAGGTGAA
ACTGTTTGCCCGGCGAGAGGGTGAATCTGGTGTTCTGCGGTGGGTTTGCTCTCTCGAGCTG
GAGTGCCCTAGTATCCAGAAGAGGCAGGCCCTACCTGATAATCCCTACCTAAACTGGGGCC
TGGCAGCAGGGGCATCGGCACTAGGTGTCAAGGATTGCAGCCTCGCTGGAGAAGAGGCT
GTGGAGGTGGGTGAGGGTGGCGAATGCAAGGTGATCCTACACACATCACGACCTCTGATG
ATGGTCTGTGAACTCGCGCACCACGAGCTCGACGCCACTACGGCCAAGCGCTGCCTTGCG
TCGCAAATCACAGCAGAGCAGCTGGTGTGCAACGTCATGTGCTTGTACAAAGGATTTTACC
GGCTGTCCGTATTTGTGCAGGATTACGATAGTGGAAGTGGGACTTTTCAAAACGCCGGGA
ACTACCTGCTGCACTGTCAAAGTCGACGGTTGAATCCAAACGTTTTGTATCCAGCAGACCT
TGGCCTGTGGTGTGGTCCTGGGATGCGTACCCAAGAAGCCGGACTGTCTCATTTCAGCCAC
ACGGGGGCAATAGTGAATGCATCACAGGGTCGGTGCAACATCACCTTCCATTGCGCCTCGC
CAGACCTGCAAATACACGCAGTCCTCTGCGCTGAGCTCCACGAGGAGGCCCATTTTCCTCT
GTCCCGATATGTACTCCTGACATTTACAGAAACTAAGGTAACTGTGAGTGTGTGCGCACCC
AGAGCAGGAGTTTATCGTCTTGCTCTTTATGGACGCAAACCCCCTCAGCAGGACTACATGT
CGCTGTGTGACTTCATAATCAGGAGTGTGTGTGAAAAGCCCGGAGATCCATTCCCCTGTGT
ATATTCAGCGTGGGGAAGAGGGTGTGTGTTGCTGGAGCCCCGCACAGGTGTGCTGGCTCC
ACAAAGTTCAGTGAGTTTTCGCGTGCGGGTACCAGGTGCTCAAAGAGTGTGTGTCGTGGG
GGAGAAACGAACGGATCTGAAGATGAATAAGAGCAGAGTTTGGGAAGGTGAGGCTGCCA
CTGGAAATGCCACACAACTCAAGCTCGCTGCTGTCACTAAAGAAAGCAACGACATGCATG
TTCTCATGACCTTTGATGTCCTTAACCTGGAGAATGAACTGTGA

>Methyltransferase-like protein 21C, CI01000299_06034445
ATGGAGGAGAGCTTGAACACTGGGGAAGATGATACAGATGAAGTTGAAGTCCGTGTGCGT
AAGGCATCCTGGGAGCCCGCTGTATTCTCCACCATTGGAAAGGAGATCCATTATTTCGCTG
GTCATGAGATCAGAATCTGGGAGTCTCTTGATTCATTTGGTTCTGTCATCTGGCCAGGAGCT
CTGGCTCTGTGTCACTACCTTGAGTCCAATCGAGCAACGGTTGATCTTCTTGACAAGGCGG
TGCTAGAGATTGGAGCCGGCACGGGTCTCGTGTCCATCGTGGCCAGTCTGTTGGGGGGTT
GGGTGACAGCAACAGACCTGCCGGAGGTCCTCGGGAACCTGAGATGCAACCTGTCCAGA
AACACACGGGGCCGCTGCAGATACACCCCGCAGGTGGCGGAGCTCTCTTGGGGTTATGAA
CTCGATAAGACCTTCCCTCATTCAGTCTACAGATACGACTACATTTTAGCAGCCGATGTGGT
CTATCATCATGACTTCCTAGCAGAATTACTGGTCACCATGCGCCACTTCTGCCAGCCCGGA
ACAACCCTCATATGGGCCAACAAGACCCGCTTCGATTCTGATCTATTTTTTTTGGAGAACTT
CAAAAAAACGTTCAATACCACCCTGCTGGCAGACAACGGAGAGGTTAAAATTTACTCTGC
CACCACAAGAGACGAAAGTGGACTAGCGACGTCAAAAGAGACCAAAGGGGTTGCGAGG
AAAGGAGAGGACCAAGATGAAGCGGTCAAGAAAGAGTCAAAGCAAGAGATTATTGAAGG
AAAGATTAAACTGACAGTAGAACAGATGCAAGAATATGAAAAGAAAAATGTGCAAAGGA
AACCAGGGTATGCTGATTTGGAAAAACAGGAGGAAACATTAAATTTGGAGGATGGAAATG
TGAACAGTATCAAAGAAGAAGAGGAGGACAACACAAACCTTGAAACTGAGGACAGTGGA
GATGAAGAGAACGACGGCTGCTCTGAGATGGATTCAACATTAGTAGAGCAGAAATCTGAG
GAAGGAAGCACAGAGAAAGAGTATGTGAGATCATGGGCACCGACTATTTACTACAGACCT
GGGAAAGAAGTTTACAATTTTTTGGGACAGGAAATCACAATTCAGGAGTCAATCGATTCTT
ACGGTGCTACTATATGGCCAGCGGCACTTGCACTTTGCCGATTCTTAGAAACACCGCAGGG

TCGTCAACAGATTGATTTACTTGACAAATCTGTACTTGAGCTTGGAGCTGGAACCGGCTTA
CTTTCGGTTGTCATCACGCTGTTGGGTGCCAAACTCACAGCTACGGATTTACCCGAGATTC
TGAGTAATCTGACGTGCAACCTCAACAGGAACACGAGAGGCCGGCGGAGGCACGAGCCC
CAGGTAGCAGAACTCTTCTGGGGTCACAAGCTGGACGAGACCTTCCCTAAATCCACACAC
CAATATGATTATGTGTTGGCGACTGATGTGGTCTATCACCACGACTTCTTGGCCGAGCTGCT
GGTCACCATGCGTCACTTCTGCCAGCCAGGAACTACTCTTGTCTGGGCGAACAAGGTCCG
CTATGCCTCAGATCTGGGCTTCATTGACAACTTCCTCAGATATTTTGATATCACACTGCTTG
AAGAGTTGGATGATGTGAGGATTTATGTAGCAACCAGCAAGACACCAGAGAAGGAAGGTG
ACCAGGTTCAAGAGACGAGCGAGAAAGTGGAGGACAACTGTGACCTTGAGTCAAACAAG
CAGGATGCCGGGGAACCAAACTCTTCAAGCGAGACAGGAAACAATGCAGAGGAAGTGGA
ACAAGAGCAGGAATGTGACAATACTCAGAGTTTAGAAAACATCAAAAAGGAGAAACAAG
TAAAGGAGGAACTCCAGGATTCTGGAAGATCTGTGGAGGAAGAGGAAGAACTCCAGGAT
TCTGGAAGATCTGTGGAGGAAGAGGAAGAACTCCTGGATTCTGGAAGATCTGTGGAGGA
AGAGGAAGAAGCTGAACCTGACAACACAGAACCAGCTGAACAGAGATCCTGGGCTCCCA
CTGTCTACTACAGTCATGGCAAGGAGATCTACTACTTTCTGGGTCATGAAATCAAGATTCA
GGAATCCATAGACCATTATGGCAGCGTAGTATGGCCAGCGGCAGTGGCCCTTTGCCGATTC
CTGGACACCGCAACAGGCCGACAGCAGATCAATCTACTTGACAAATCGACCCTGGAGCTT
GGAGCAGGAACAGGCCTTGTCTCGATAGTTGCCACACTTCTAGGTGCTAAACTGACAGCC
ACAGATCTGCCAGAAATCCTTGGAAATCTGAGGTGCAACTTGAACCGGAACACCAGACAG
CAGCGAAAACATGAGCCTCAGGTTACAGCATTGGCATGGGGCTACAAACTAGAGGAGATG
TTTCCCCGCTCCACCCACCACTATGATTATGTATTGGCAGCTGATGTGGTCTATCACCACAA
CTGTCTCACTGAGCTTCTCGACACCATGCGTCACTTCTGCAGACCGGAGACAACTGTAATC
TTTGCAAACAAGGTCCGGTATCAGTCCGACCTGGTATTTATAGAGAACTTTCAGAAAGCTT
TTAACACCACATTACTGACAGAGCTGGATGAGAAGGCTTTCCAACCTACACTGCTGGCAG
AGGTAGAAGAGGTGAAGGTCTGCATGGCTGTGGCGCATGGACGGCCGTTTGTTTAA

>Solute carrier family 13 member, CI01000041_00392440
ATGACAACTAATGAAAAGCAGGAAAATAGAGAAATGCAACAGCACTCCATCCACAACACA
CCTGACATCTGCAGCCGAGGGCCAGAAGACATGGGACTTCCAAGGATTCCGTTAGTTCTA
CGCTGGATATGGCTTCACAGAAACTACCTGATCATCTTCATCACCCCTCTTCTTATATTGCCC
CTGCCACTGGTCCTCCCAACTCCAGAGGCAAGATGTGGTTTTGCAATCATCCTAATGGCGC
TGTACTGGTGTACAGAGTGCATGCCGCTGGCCGTCACTGCCTTGCTGCCTGTCGTCCTCTT
CCCCATGATGGGCATCATGGAGTCTGGAAAGGTGTGTGTTCAGTATCTGAAGGACACCAAC
ATGCTGTTTATTGGGGGTCTGCTGGTGGCTATAGCCGTTGAACACTGGAATCTCCATAAGCG
CATCGCTCTGAGTGTCCTCCTGATAGTTGGAGTCCGGCCTGCTTTGCTGATGTTGGGGTTCA
TGATCGTAACGGCCTTCCTCTCCATGTGGATCAGCAATACGGCCACCACAGCCATGATGTT
GCCCATTTCTCAAGCTGTTCTTGAACAGCTCTGTGCCACAGAAGCAGACTCTGATGAGAA
AGAGCTGAGGGAAGGCCAAGATAATCAAGCGTTTGAGTTGACCGAGGTCAACATCAAACA
TCCTTTGGACAATGTCTCTGGAGACAAACCCAACGACAATCCAGATCTGAAGGCAAGATT
GGATACACTCTCTGAACTCAGGAGGAAAGAACGTGAAGTGAAGTACCTTCATCTCTTTAA
AGGAATGAGTCTATCTGTGTGTTACTCCGCCAGCATTGGAGGAACTGCCACCCTAACGGGC
ACCACACCAAATCTAATTCTCAAGGGCCAGATGGATGAGATTTTTCCAGAAAATCATGATG
TGATCAACTTTGCCAGCTGGTTCGGTTTTGCCTTTCCTAACATGGTGTTGATGCTTGCTCTG
TCTTGGCTTTGGCTGCAGTTCATGTATTTGGGCTTTAATTTTAAAAAATCTTTTGGCTGCGG
TTCGAAGAACGAGGGAGACAAAGATGCTTACGGAGTGATGAAGAATGAATATAAGAAGTT
GGGTTCGATGTCTTTTGCTGAGGGCGCAGTGCTTGTCATCTTTGTGATCCTGGTCATCCTGT
GGTTCACACGAGAACCAGGCTTCATGACAGGCTGGGCTACTGAGCTCTTCAACAAAAACG
GACAATATGTCACTGATGGTACCGTGGCCATTTTCATGTCAACCCTGTTCTTCGTCATTCCA
TCTCGGATAGACTTTCTCTATTCAATCAAATCGCATGATGAACAGGATGAAGAAGCTGAAG
GAGCTGAGCAGGAAGGAGAAGCAAAACAGGAAAAGAAGAAGAAACTGAAGGGTACGCC
CACTCTACTAAACTGGAAGGTTGTGCATGATCGCATGCCCTGGAACATAGTCCTGCTCCTG
GGGGGAGGATTTGCCTTGGCAAGTGGAAGTGAGGAGTCTGGACTATCGCTGTGGTTGGGT
CAAAGCCTGGCCCCTCTGCAGAGCATCCCACCCTTCGCCATCTCTCTGATTTTGTGTTTGCT

GGTGGGAATGTTCACAGAGTGTTCCAGCAACACAGCCACCACCACGCTTTTCTTGCCCATT
CTTGCCTCTATGGCCACAACCATCGGGCTCCATCCTCTCTATGTGATGCTGCCTTGCACGAT
TAGTGCTTCGCTGGCATTCATGCTTCCCGTAGCCACTCCACCCAACGCCATCGCTTTCTCTT
ATGGCAACCTCAAAGTCTTGGACATGGCAAAGGCGGGCTTCATTCTCAACATCATTGGCAT
TCTGTGCATCAACCTCGGCATCAACACATGGGGAGCGGCCATGTTTAAGCTAGGAACCTTT
CCCGCATGGGCCAATGCCACAAATACCACAAATACACCATGA

>Long-chain-fatty-acid--CoA ligase 1, CI01051147_00003814
ATACGGTTTGCTGTAGTCATGCAGGCACACGAGCTGTTAAAGCAGCTGAGAATCCCTGAG
CTGTCCGAGGTCAGGGATTATTTCCGCAGCCTCTCCACCAACACACTCCTCGGCATGGGCG
CGTTCACCGCCGCCACGGCGTACTGGTACGCCACGAGACCCAAAGCCCTCAAACCGCCCT
GTGACCTGCGCATGCAGTCGGTAGAGCTCCCGGGTGGAGAGTTTGCTCGTCGAGCGGCCA
TTTTAAACGGAGGCCCACTCTTGACCCATGTCTACGAAGATGCCAAGACCATGTACGAGTG
CTTTCACAGGGGCCTGAGGGAGTCGAAAAACGGTCCCTGTCTGGGATCCAGAAAACCAA
AGCAGCCGTATGATTGGCTTTCATACTCAGAGGTGATTGAGCGAGCGGAGAATTTGGGATC
AGCATTTCTACACAAAGGACACTCAAAAAACGGAGACCCCTATGTCGGCATCTTCTCTCAG
AACAGGCCAGAG

>Calcium-binding and coiled-coil domain-containing protein 1, CI01000057_01092073
ATGGAGGAAAACAGAACTGTGAAATTCAGAGATGTTGCAGAGAACTATTTCTCACAGACG
AGGGTGGACTGTCGCTACACTATAAGCAGTCAACACAGTTGGAGCAGTCATGACTGGATA
GGTCTATTTAAGGTGGGATGGCTGACAGTGAAAGATTACTACACTTTTGCCTGGGCTCTAG
CACCGGAAGGGTACCAAACGGGAACAGATGCAAACTGTAGTGTGGCCTTTTACTCATCTTA
TCTACCAAAACCCGAAGGGGAGGCGTATCAGTTTGTGTATGTGGATGAACATGGTGAGATT
TGTGCGGTCAGTTCTCAGTTTACTTTCTGTGCTCCAAGACCTCTGGATGAACTTGTTACGCT
CGAGCAGGAGAAAAACGGTGAGGAAGAGGAGGAGGGAGATGACCTGCTGCTGGTGGTG
CCGAAGGCTCTGATCCTGCAGAGTCTCTTAGAAGGATGTCAGCGTGAGTTACACGAGTTG
CATAAGAGGCTTGAGGTTGCAGATGATGAGGTGGAGAGAGAGAGGGAGAGATGGAAGAC
GGACAGAGATGAGTTTGAAAGAGATGTGCAAAGCACACATGAAAACATGACTGCTGAAC
GTAGTGGACTTCTAGCAGAGAGAGCTGAAAACCATCAGCGAATCAAAGAACTCAAACAG
GATGCTGCCACTCTGACTCAACAAAAACAGGACATAGAAGCCGAGCTGGACAGAATGAA
GGAAAGAGTTAAAAAGATGACCACACAAAGGCGAGATGAAGAAAAGGAGAAGAAAGAT
TTGCAGATTGAGAATGAGCATTCACATGCTGAGCTGCAGACCTTGCAGGAGCGTCTGGAG
GCCAGCGAGCGCAGCGCTGACGCCCTCCGCAGGGACCTGAGTGAACTGGGCTCCATGCA
GAGTCACAGCCATGCAGAGCTGCATCAGGTCAGACTGCAGTCTGCACAGATTAATTTACA
ACTGTCCCAGGCTAACCTGGCTCTGCGTGAGGGACAATCCACCTGGGCCCAGGAGAGAG
AGACTCTGAGACAAAGTGCAGAGTTGGATAAGGATCGTGTCCAGAAACTGAGCCGCGAG
CTTCAGAAGAAGGAAGAGTGGCTCCAAGAAGAGAGAACAGAGCGAGAGAAACTGGAAG
TGGAGCTGGGGAACGAGAAGGATTGTAACCGGGAACTGAGAGCCAGTCTGAGAGCCCTG
CAGAAGGAACAAGAGCAGCATCAGCTGGAGAAACAGTGTTTTATTCAAAAACAGGAGCT
CTTAGATCATATCCATGTGCTGGAGCTGAGGCTAGACACTGAGACAGATGCTAAGTGGGCT
GAGGCAGCGGCTACACCAACATATTGCATTGATAGACCTCCATCAATTGAAGAAAAGCCAC
CACAAGAGTCTTTAAGTGTGCTGGACAGTGAGCAGTTTAGCTCTCATGGGTCAACAGAGC
AAAGCCAAAAGGATAATGAGAACGATATGATGGCAAGTACCACAAAAATGAAATAA

>Suppressor of cytokine signaling 2, CI01000020_00951237
ATGACCTGTCACTCATCCGACTCCACGGAAAGCATCGAGAATGAAAGGAGATCGCAAACT
GAAACCCAAATCACGGAGTCCGAACAGAGTCGCATTGCCACTGCCATGAGAGACCTTAAA
AACACCGGCTGGTATTGGGGCAGCCTGACAGCCAATGAAGCCAAGGAGATCCTGCAGGA
CACGTCGGAGGGCACATTTCTGGTCCGGGACAGCTCTCAGAGGGACTACCTCTTCACCAT
CTCCGCCATGACATCTGCGGGACCAACCAACCTGCGTATCGAATACAAGGACGGCAAGTT
CAAGCTGGACTCCGTGGTGCTGGTCAAGCCCAAACTCAAGCAGTTCGACAGCGTCGTCCA

TCTTGTGGAGCACTACGTGCAGCTGTCCCGGACGTCTTGTAAGGGCAGGACTACCCCTCTC
GCCCCATCCAACGGCTCTGTGCAGCTGCTGCTCACGACTCCGGTGTACACAGCCACACCG
TCTCTGCAGCATCTGTGTCGTATTGCCATTAATAAAACCACACGGCGGGTACAAGAACTTC
CTTTGCCTAACCGGCTGAAGGATTACCTGACAGACTACACCTATAATGTATAG

>Ankyrin repeat domain-containing protein 29, CI01000030_01431670
AAGGAGACTCCTCTTGCCAACGCTGTGTTTTGGGCAGCAAGGAAAGGAAACTTGGCTTTG
CTTCAGCTGTTGTTGAACAGCGGCCGAGTGGATGTAGACTGCAAAGATGGTCAGTATGGC
ACCACTGCGTTAATGGTGGCATCATACAGTGGGCATTATGAGTGTGTTCGGGAACTCATCAT
GCAAGGTGCAGATATTAATCTGCAAAGAGAGACAGGTTCCACCGCCCTCTTCTTTGCTTCA
CAACAGGGCCATAATGAAATTGTCAAGCTCCTGTTTGAATTTGGAGCGTCCACTGAGTTTC
GGACAAAGGATGGCGGCACAGCACTCTCCGCAGCCTGTCAGTATGGTCACTCGACAGTGG
TGGACACCCTACTGAAGAACGGCGCCAATGTCCACGACCAGCTCAATGATGGTGCGACAG
CCTTATTTTTGGCTTCCCAAGAGGGTCATGTGACTGTAATACGTCAGCTTATGTCATCTGGG
GCTAAAGTCAATCAACCTCGAGAGGATGGCACAGCTCCGCTCTGGATGGCAGCCCAGATG
GGACATAGTGAGGTGGTGAAAGTTCTGCTGCTACGAGGTGCGGACCGTGATGCAGATAGA
AAAGATGGCTCCACGGCTCTGTTCAAGGCTGCACATAAAGGATACTGTAACGTCATTGAGG
AACTGCTCAAGTTCTCCCCTTCACTTGGCCTTCTGAAGAACGGTTCTACAGCTCTTCATGC
AGCCGTCATGGGCGGGAATCCGAAAACAGTTGCACTGTTGCTGAAGGCAAATGCCGATCC
AGCTTTACCCAACAAAGTCAGTTTGAGACAACAAGGAACTGCCGGAGGATCTTACGAGGA
ACGAGCGCATCCTAAGAATCCTGCGACTGCCGATGCTGAATGGAGGGAGTTGATGACTGC
TGTGTTTGTTTTTAAGTCCATGACAAATGAATGCACTACATCTCAGTCCCTTGAGGCTGAA
AACAGCAGTCGCTGCCTGCAACAGCTTTCCAGTCTCGTACAGCGGATGCTTGAAGCTTCA
GATCAACAACACAAATGCACTGAAAATCAGAATTGCTTATTCTTTATCCTGGAAATGTTATG
A

>Apolipoprotein A-I, CI01000195_00994487
ATGAAGGTTCTTGTGGTGCTTGCACTTGCTGTATTTACAGGTTGCCAGGCCAACCTATTCTA
CGCTGATGAGCCCAAGCCACAGCTGGAGCAGCTGACTGATGCATTCTGGAGCTATGTTTCC
AAGGCAACACAAACCGCAGAGGAAACCGTCAAGATGATCAGGTCTTCCCAACTGGGACA
GGAAGTCAATGCTAGACTGACCCAGAGTGCAGACATGGCCAGCGAATATGCCGTCACCCT
CAAGAAACAGATGGATCCTCTGGCTGAAGAGCTGATGACCAAAATCACCAAGGAAGCTG
AAGTGTTGAGGGAGCGTTTGGGCCAGGACCTGATCACTGTGAGAGACCAGCTGGAGCCCT
ATGCTGACAACCTCAAGAGCCAGATCCAGCAGAGAGTGGAGGAGCTCAGGACGGCCATG
GCTCCATATGCAGATGGATTCTGA

>Type-4 ice-structuring protein LS-12, CI01000027_01519711
TCTGAGTCAGTATCTCTGGTCAAGAGAGACGCTCCTGCTGAGCTGGACCAGATCGCCAAG
TACTTCCAGGACCTTGTGGACAATCTGAAGAACGTTGAGGGCCCTGAGCTGGCCAACAAG
GCCAATGCTTACCTCGAGCAGAGCAGAGCCCAGTTCCAGCCCATGATTGAGAAGCTCCAG
GAGCAGCTGAAGCCCCTCTCCAGCAACATTGAAGAGCACATCAAGCCTCTGGCCGCCTCC
GTCCAGGCTCAGGTCGCCCCCCTGGCCGGCATGGTCCAGACCCACGTTGAAGACGTCCTC
AAGTTTGTGGCTGACAAGACCAAAGCCATCCTGCCGCCTCAGTAA

>F-box only protein 32, CI01000027_05795504
ATGCCGTTTCTTGGACAAGACTGGCGGTCTCCTGGTCAAAGCTGGGTGAAAACGGAGGAC
GGCTGGAAAAAAACGATCAAAGATGAAAACGAGACGAATAACAATGTTTCAGAACGGAA
GAGCTACTGCAAGGAAGAACATGACAAGGAGAATTTGATCCTCAGCATTAATTATGATGTG
GCTGCAAAGAAGAGAAAGAAGGACTTGCTAAACAACAACACAAAGATTCCTTATTTTTAC
AAAGACAAATGGATTTACGTCCACAAAGGAAGCACCAAAGAGCGACATGGATATTGTACT
CTGGGAGAAGCATTTAATCGCTTGGATTTCTGCAGTGCCATCAAAGACACCAGGCGATTCA
ATTATGTCGTACGGCTGCTGGAGCTGATCGCCAAGTCCCAACTGCCTTCGCTGAGTGGAGT
GGCACAGAAAAACTACATGAACATTCTGGAAAGGGTTGTGCAGAAAGTTCTTGAGGATCA

GCAAAATGTTCGACCAATTAAAGAACTGCTGCAGACACTATATGCGTCGTTGTGCAGTCTG
GTTCAGGACATGGGCAAGTCTGTCCTGGTGGGCAACATCAACATCTGGGTGCACCGCATG
GAGAACATACTACAGTGGCAACAGCAGCTTGACAATATTCAGATCAACAGGCCTACAAAC
ACAGGGATGACATTCCTTGATCTGCCGGCTAGCTTACAACTGAATATCATGCACCGTCTTTC
AGACGGACGAGATCTGGTTAGCCTGGGTCAAGTATGCCCTGACCTAAGTGTGCTGACAGA
AGATCGGCTGTTGTGGAAGAAGCTCTGCCAGTATCACTTTGCAGACAGACAGATCCGCAA
GCGCCTAATAGTGTCAGATAAAGGACAATTGGAATGGAAAAAGATGTATTTTAAGTTGTGC
CGGTGCTACCCTCACAAAGAACAGTACACTGACACGTTACAGTTCTGCACACATTGCCAC
ATCCTGTTCTGGAAGGATACAGACCATCCTTGCACTGCCAATAACCCAGAGAGCTGCTGCA
AGCCTGTCTCTCCACAGGGTTTCATCAACCTTTTCAAATTCTAG

>Nociceptin receptor, CI01000021_04476829
ATGGAGTTCCCAAATGATTCCACCGGCTTTGCTGACCCCAGACACTTCCGTCTGTACAACG
AATCCCTGTTCCAGAACAACCTCAGCTCCTTCAACGGGACTGACAGCTTCTTCCCAAGGG
GTGTTAAGATCACTATAGCAGTGGTCTACATGATCGTCTGCGTGGTGGGGCTGGTTGGAAA
CTGCCTGGTCATGTATGTCATTATCAGATATACCAAGATGAAGACGGCTACCAACATCTATAT
CTTTAACCTGGCACTGGCTGATGCACTGGTTCTGGCTACGCTTCCCTTCCAGGGCACAGAT
GTATTCCTTGGTTTCTGGCCCTTTGGCAATGCCCTGTGCAAAGCGGTCATCTCCATCGACTA
CTATAACATGTTTACTAGTGTGTTCACCTTGACCGTGATGAGCATGGATCGATACGTGGCCG
TGTGCCACCCAGTGAAAGCCCTGGACATGCGGACGCCCCACAAGGCCAAAGTGGTCAATA
TCTGCGTGTGGGTCTTGGCTTCAGCCATAGGGGTCCCGGCTATGATGTTTCATGGAAGTGA
TGATGTAGCAGGCAGGAAGTCCAGATTCACACATCTCAGGAATGGAGAGGCTTTTAATGCT
CTCAGATGCATCGAGTGCATCTTGGTTCTCCCGGATCCTCGCAGTTACTGGGACCCGGTGT
TCGGGACGTGTGTTTTCCTTCTCTCCTTCCTGATTCCTGTGGCGATCATCAGCGTGTGCTAC
AGCCTCATGGTGAAGCGCCTCCGCAGCGTTCGCATCCTATCAGGATCCAAGGAGAAAGAT
CGCAACCTTCGGCGCATCACGCGTATGGTGTTGGTGGTAGTCGCGGCTTTCGTTGTATGTT
GGACGCCCATCCAGATCATGGCCCTGGCGCAGTCACTGGGCTTCAACTTAGCCAGCGTCC
AGACGGTGGTGTTTATGCACTTCTGCATTGCCCTGGGTTATGTCAACAGCAGCCTTAACCC
CGTCCTCTATGCATTCCTCGACGAAAACTTCAAGCGCTGCTTCCGCGAGTTCTGCCACCCC
TCACCATTTGGCCTTGATGCGCAGCAGTCGAGCCGCATGCGCAACATTGCGCGTGAGGTG
GCCTACAACTGCAAGACTGCGGATGGGAATAGTAACCCCGCATGA

>Krueppel-like factor 9, CI01000339_03378630
ATGACGGACGTTGATATTGCAGCAAGGTCCTTGGAGCCGGTCTCAAACGGCTCCAGCTCT
GAGCGCTCAGACAAACTATCCGGCGAGGAAAGACCTGAAGAAAGTCAAGAGAGCCGGAC
TCTGTTAATGGTCGCAATGATTCTGCTGGACTTAAATCAGTGTAACCCAAACGGAATCAGC
ACCAAACGTATGGTGGACAGTGATGCGGAGAATCAAACCAGCGTAAAGGAGAACCGGGA
GAAATGCAGACAGAACGCAAGCAGGGTGAAAAGGACACGGAACCGTGACAGTCTGCATG
TGTCTGAGAAGAGACACTGTTGTCCCTATGCTGGCTGTGGGAAAATATATGGCAAATCGTC
CCATCTCAAGGCGCACTTCAGAGTACACACCGCGATCACCTCACCAAGCACGCCCGCAGA
CATGCTGGCTTTCACCCCAGTATGCTCCAGGGTCCCGCCGGACGAAGACGACACTCTTCA
ACGTCCACATCGTCCTCTGGTTCCAGCGATCACATGTCTGCTGGTGTTTGAGTAA

>Ankyrin repeat domain-containing protein 37, CI01000300_05954888
CTGGACTGTACGAGTAATCTTTTTGAAAGCGGATTTGCTGTGAACGGAGGTTTGGATGGTC
CCGGACACGGACAGTCTCCTGCGCATCTTGCGGCATGCGGGGGTCAGGCTTTCTGCTTGCT
TTGGCTGCTGCAAACAGGTGCTGATGCAAACCAGCAGGACGCAAGTGGAGAGACGCCCA
TTCATAAAGCAGCCAGAGCGGGCAGTTTGGAGTGCATTAGTGTGCTGATGGCAAGTTATGC
ACATTTCGACATTTGCAACAATGCTGGACAGACAGCTGAAGATATTGCATGGTCTTGTGGG
TTTGAGGAATGCGGGAGGTTTTTAAACATGCATCGGAGAACACAGGACTTAAAGAACGCT
TCATCATCCCCTCTCGCTGAGCGGCATATACCTGCCAACACAGAGCAGATGCCAATCAAGG
CAAAAGCAGAGAATTTCCTGGTGGAGGTTGAGATTGCACTGGAAAACGTGCAACATTTAA
CTTCGATCACATACATCTTGTGA

>Nuclear receptor coactivator 7, CI01000027_00141799
ATGAGGCCAATGCAGGAGAGCATTAAGATCTTGTACTTTGCTAATGGATCCGAGGAGCCGT
TTGTGGAGATCGTCACTGTCAATCAGCAGAAGCGTCGTCCGAGTGTTTGCAGCTCTGCCG
ACTCTGAATCCGAGGACCTGCAGCCAGTTTTAGTAGATCGCAGTCAGATCCTGAAAGAAC
ACCATCTCGAACAACTGATGAACCACATTCCGGCTCGGACTCAAGGTTATCCGTGGAAGCT
GGTGTACAGCACGGCGGAGCACGGCACCAGTCTGAGAACACTGTACCGGCAGATGGCAG
AAATAGACAGACCCGTCCTGATGGTCATCAAGGATTCAGACAATCAGGTGTTCGGAGCGTT
TTCCTCTGACCCTTTCAAAGTGAGCAGCTACTGCTACGGGACGGGAGAGACGTTCCTCTA
CAGCTTCAGTCCAGAGTTTCAGATCTTCCGCTGGAGTGGAGAAAACTCCTACTTCGTGAG
AGGATTCCTGGACTCTCTTCAGATGGGAGGAGGAGGGGGTCCGTTCGGATTGTGGCTGGA
CGCTGACCTGTATCGCGGCTCCAGTTACTCCTGCGACACCTTCTGCAACCGGCCGCTCAGC
CTCCATCACGACTTCACCATTCAGGATCTGGAGGTCTGGACCTTCATCTGA

>Kelch-like protein 38, CI01000027_05784234
ATGTGCTTTAAATCAGCTGAGACAAGCTTAGATGGTCCAATATCAGAAGTCCTGCATTTTAA
AGACAAAGACCTGATCTCCAGCCTCCTTCTGGAGCTGAACGCCTTGAGGAAGGACCAAAT
TCTCACTGATGTTGTTCTTTACTCTGAGGGCAAAGAGATCCCATGTCACCGCAATGTTCTG
GTGTCCAGCAGCCCCTACTTCTACGCTATGTTCTGCAGCTGCTTCCTGGAGACCCAGAAGC
CCCGGATAGACCTCAAAGGTGTTCCTTATGACATTCTTATGGGTATAGTAGAGTATGTTTAC
ACAGGGTCCATCAGCATCACCATGGAGCAGGTTCTCCCTCTGATGCAAGCCGCATCTATGC
TTCAGTATGGAAGACTTTTTGAGGCCTGCTCGACCTTCCTGCAGACCCAGCTCAGTCCGGA
CAACTGCCTGAGCATGATCCGGCTCTCTGAAATCCTGCATTGCTCTAGCTTGCAGGAAAAA
GCGAGAGAACTGGCTGTCAAGAGCTTCTCCGATGTGGTGGTCTCCGAGGACTTCTGCGAA
CTCTCTCTACCCGAATTAGTGAGCTACTTGGAGGACGACAAACTGTGTGTTGAGGAGGAA
CAAGTATTTGAGACTCTTCTAGCTTGGATTCACCACGACCCATTCTCCAGACGTGGCACTAT
CCACGACTTGTTCCGTCGTGTGAGGCTGAGACACGTCCATCCTTCATATCTTTTCCAGTTTA
TTGCCAATGACCCTCTAGTCCAGTCGTCTTCATTATGCACAGAAATTATTGAATCCGTTCGT
CGTCTTCTTTTTTCTGTTGGCACTCATTGCCCTGGTGATCTTGAGTCCCTTTGGGCAGTGCC
ACGACGGCAAAACTGCAAGGAGGCGCTTGTAGTAGTGGGAGGTCGTAAGAACGGTGAGC
GGACATCTCGTGAGGCGCTTCTTTACGACGAGCAGACTCGCTGCTGGCAGTGTCTCGCAA
AAATCCCGCTGCGCCTTTACCGGCCCTCTTATGTCTGCATGCACAGCATCCTTTATGTGCTC
GGGGGCTTAACCATGAGAGCAGAAGGGCAGTGCACAGCTAGCAACAACGTGTACACGCT
TTCTCTGAAAACAAACCAGTGGAGAACTGGAGAACCAATGCTGACGCCTCGATACGCCCA
TCAGAGCTCCACCTACCTGCATTTCATTTTTGTTCTGGGTGGATTGACGGCTGACGGACAG
CTGTCCAATGAGGTAGAGCGATACGACACCATGTTCAACCAATGGGAGGTCATGGCCCCCA
TGCCCACTGCAGTTTTGCACCCAGCTGTAGCCGCACATGACCAGAGAATATACATCTTCGG
AGGGGAAGACGCCATGCAGAAACCAGTTCGAATGATCCAGGTGTACCACATAGGAAGGAA
TCTTTGGTATAGGATGGAAAACAGGACGGTGAAGAACGTCTGTGCACCAGCTGCAGTGAT
CAATGATAAAATCTACATTGTAGGCGGCTATACAAGAAGGATGGTAGCCTATGATGTGAAA
ACCAACAGGTTTGAAAAATGTGAAAATATGAAGGCCAGGAAGATGCATCACTCTGCTGCT
GTTGTGAATGACAAGATCTATGTCACGGGGGGCCGCTTCATTAACGGTCACGAGAGTGTG
GAGGACTCGGACGACTTTGACTGCTATGACCCAAAGACAGACTCATGGGTGTCGATGGGA
ACTTTGCCATTCAAGCTATTTGACCATGGCTCAGTAAATCTGGTCTATCTCTCTGATAAGTTT
TTGCCTACGTGA

>Myosin regulatory light chain 2, skeletal muscle isoform, CI01000069_04133876
ATGGCCATGTGGCAGGGTGAGGCGGGAGGACTTGGACCCGAATCTTACGGCATCCCTATAC
TAGGAAAGGCATTAAAACCCTATTTTCAATTAGGGCTGATTTGGAAAGAGGCGATGGCAAA
GGTTATGACGCCAGAACAGCCTTTTATATATCAGGCACCCAAAAAGGCTAAGAGGAGGGC
AGGTGGAGGAGAGGGTTCCTCCAACGTCTTCTCCATGTTTGAGCAGAGCCAGATTCAGGA
GTACAAGGAGGCTTTCACAATCATTGACCAGAACAGAGACGGCATCATCAGCAAAGATGA
CCTTAGGGACGTGTTGGCCTCAATGGGTCAGCTGAACGTGAAGAATGAGGAGCTGGAGGC

TATGATCAAGGAGGCCAGCGGCCCAATCAACTTCACTGTTTTCCTCACCATGTTTGGAGAG
AAGCTGAAGGGTGCTGACCCCGAAGACGTCATTGTGTCTGCCTTCAAGGTGCTGGATCCA
GAGGGCACCGGCACCATCAAGAAGCAATTCCTTGAGGAGCTTCTGACCACTCAGTGCGAC
AGGTTCTCTGCAGAGGAGATGAAGAACCTGTGGGCCGCCTTCCCCCCAGATGTGGCTGGC
AATGTTGACTACAAGAACATCTGCTACGTCATCACACACGGAGAGGAGAAGGAGGAGTAA

>Parvalbumin-2, CI01000351_01710164
ATGGCCGTTGCTGCTATACTCGCCGCAGCTGATGTGGATGCTGCTATCGCTGCCTGCCAGG
CTGCTGACTCCTTTGACTACAAGTCCTTCTTCGCCAAGGTTGGCCTGTCAGCCAAGTCCTC
TGATGATGTCAAGAAGGCTTTCGCCATCATTGACCAGGACAACAGTGGCTTCATTGAGGA
GGAGGAGCTGAAGCTGTTCCTGCAGAACTTCAAGGCTGGTGCCAGGGCTCTGACCGACA
AAGAGACCAAGGCCTTCCTCTCTGCTGGTGACAGTGATGGTGATGGCAAGATTGGAGCTG
AAGGTAAATGA

>fatty acid binding protein 6, CI01000006_02159922
CACTATCCCATGATAGTCCTCAACTTCACTGATAATAATATCTCATCATCAAATGACCTTAGA
AGCTTCAAAGAAATCTCTAAATCCAGACAAATGTCACACCATTCCATGTTCCCCAGGAATC
AATGTGTGCTTTCAGAGAGGCAGAGTTTAATAGCGGTAGGGTATATAAGTGCAGGGAACTC
GATCCATCAGTACCTCAACTTTCAGTTCACTCTTCTTCTCCGCGAAACAATCAACACCCAA
ACCATGGCTTTCAACGGCAAGTGGGAAACCGAATCTCAGGACGGATATGACGAGTTCTGC
AAACTGATTGGTATCCCTGATGATGTCATCACGAAGGGCCGTGACTTCAAGCTTGTGACGG
AGGTCATCCAGAATGGAGATGATTACACATGGATCCAGTACTACCCAAACAACCACGTTGT
GACCAACAAATTCATCGTTGGCAAAGAAAGTGACATGGAGACTGTTGGAGGGAAGAAATT
TAAGGGTGTAGTCTCCATGGAAGGGAGCAAGCTGGCCATAAGTTTCCCCAAGTACCACCAT
ACATCTGAGATCAGCGGTGGAAAGCTGGTGGAGACATCCACAGCAACTAGCGCCAAGGG
TCCAGTTGTTTTTGTTCGTACAAGCAAGAAGATCTAA

>Elongation of very long chain fatty acids protein 4, CI01000001_00131285
ATGGCCGATGATGTCGTCGCCGCTCCCCACCCTGGGGATCAGCGTTCTCTACCTGCTCTTC
CTCTGGGTCGGACCCCTTTACATGCAGAACCGCGAGCCTTTTCAGCTCAGGAAAACCCTC
ATTGTGTACAACTTCAGCATGGTGCTGCTCAACTTCTACATCTTCAAAGAGCTTTCTTTGGC
GCTACAATTAACTCAGGCATTCATGTGCTGATGTATGGCTACTACGGCTTGGCAGCGTTTGG
TCCAAAGATCCAGAAGTACCTGTGGTGGAAGAAATACCTCACTATTATTCAGATGATCCAG
TTCCACGTCACCATTGGTCATGCCGCTCACTCTCTCTACACGGGCTGCCCGTTCCCGGCCT
GGATGCAGTGGGCTTTGATCGGCTACGCCGTCACGTTCATCATCCTGTTCGCCAATTTCTAC
TACCAGACCTACCGCCGCCAGCCACGGCTCAAGTCGGCAAAACCTGTCATTAATGGCTTCT
CCACAGCAAGTAATGGCACCACTAAGGCACCGGAGGTCACGGAAAATGGAAAGAAACAA
AAGAAAGGAAAAGGAAAACATGACTGA

>Heme oxygenase, CI01000023_00367202
GAAATGGAGACGATTGAGAAAACACAGACCGCAATCACAGACAGTGAACTATCAGAGCA
GATCAAAGCTGCGACTAAAGACAGTCATGTGCGAGCCGAGAACACGGAGCTGATGCTGA
GCTACCAGAGAGGAAACGTGTCCCTCCGACAGTACCAGCTGCTCTTGTGTTCGCTGTATAA
GATATACGAGGCGTTGGAGGAAGCGCTGGACAGGAACGCCTCCCATGATGCCGTGGCTCC
CATATACTTCCCACAGGAACTGGCCCGGCTGGAGTCCATTGAGAAAGACCTGAAGCATTTC
TACGGCCAGAACTGGAAGGAGAAGATCACTGTGCCGGCCGCCACGCTGAGATACGCCCA
GAGACTGAGAGAGGTGGGCAGAGATCATCCTGAATATCTGGTCGCTCACGCGTACACGCG
CTACCTCGGCGACCTGTCGGGCGGGCAGGTGCTGGGTCGCATCACGCAGAAGTCATTAGG
GCTGAAGAACGGAGAGGGCTTGTCGTTCTTCTCGTTTCCCGGTGTGAGCAGCCCCAACCT
CTTCAAGCAGCTGTACAGGAGCCGCATGAACAGCATCGAGCTGACGGAGACTCAGAGGA
ACGGCGTGCTGCAGGAGGCCGTCACCGCATTCGAGCTCAACATCCAGGTCTTTAACGAGC
TGCAGGACTCGGTGAGCTGCGTGTCAGAGACGGAAAATGAAATGAGACAGAGACACACA
CAACACAAGCAGAACACAACAACGACAGCAGGTGATGAAGCGCTGCAGAGCTCCTCGTC

TCTGATGCCGCGGTTCCTGCGGACGCTGCTCGGTGTGTCTGTGGTTCTGGCCGTCGGGATG
GTCTACGCCTTTTAA

>Insulin-like growth factor-binding protein 1, CI01000190_01575895
ATGAGAGCGCCGTTTGTGTTTGTGATGTTGGTGAGCGTCCTCGTCCATCCGGCGGATCTTT
CCCCCGTGATCGGGCCTGAACCCATCCGCTGCGCTCCGTGCTCACAGGAGCAGCTCGCCG
CCTGTCCCGCCGTGTCCTCAGACTGTCTGGAGGTCCTGCGAGAGCCGGGCTGCGGATGCT
GCTCGTCCTGCGCGCTGAAGAAAGGAGACTCCTGTGGCGTCTACACGGCTCACTGCGGCA
CCGGCCTGCGCTGCGTCCCGAGGCCTGGAGACCCACGGCCGCTCCACGCACTGACCCGCG
GTCAAGCAGTCTGTGCAGAGTATGACCAGACAGAAGAGGACACAGACATCACTTCTGATC
AGGGCGCGCTGCATTACCTGCTGGGACTCAACAGGCCGCTCGACCCGCAGGACATGGCCG
AGGTGCAGGAGAGCATCAAGGCCAAGGTCAACGCCATCCGAAAGAAACTGATCCAACAG
GGTCCATGTCACACCGAGCTTCACGCCTCTCTGGACGTCATCGCACAATCACAGCAGATGT
TAGGCGAGAAGTTCACGAGCTTTTACCTCCCCAACTGTGACAAACACGGATTCTTCAAGG
CCAAACAGTGCGAGACATCTCTCATCGGCCAGCCCCCACGCTGCTGGTGTGTGTCGTCCT
GGAGCGGAAAGAGAATCCCAGGAACTGATGATTTATCGGCTGATTCCCATTGTCATCAGGA
GCTCACGCACTGA

>SPRY domain-containing SOCS box protein 3, CI01000329_01153690
ATGTCTTGGAATAAGTATGCTGTTAGTGAAAATGACCCGGAGACTGAAGCCACGCCCATCA
ACACGCCCCTGGAGACTGAAGCCACGCCCCTCCGCCCAGAAGACAAACAGGCTGTCGCA
GCGCAGTCGGAGGCTGACTCTACAGGGTACGAGGCCGTGACGGTGTGTGTGCCAGCGGA
GATGCCACCGGTGGTGCCCGTGACGGGCGAGTCGTTCTGCCAGTGTCCAGCTCAAACTGA
ACTCAGCTCAGACGTCCAGATCTGCCCGTACATCCACAGCTGCACCTGTGGAGAGGAAGA
ACAGGGATGTGATTGGGTTTGGGATGATGAGGGCAAATCATTCTCCGTGTCTCTGAGCTGC
TCTAACCGGAAGGTGAGCTTCCATTCGGAGTACAGCTGTGGAACCGCCGCCATCAGGGGC
GCAAAGGCTCTCAGTGACGGACAGCACTTCTGGGAAATCAAGATGACATCACCCGTCTAC
GGCACAGATATGATGGTTGGTATCGGGACATCAGAAGTGAATCTGGATCAGTTCAAACATA
GTTTCTGCAGTCTGTTGGGCACTGATGAGGACAGCTGGGGTCTGTCCTACACAGGTCAAC
TCCATCATAAGGGGGTCAAAGGTGAACTTTTCTTCACGGTTTGGTCAAGGGTCCATAATCGG
GGTTCATCTGGACAGCTGGCACGGCACCCTGAGCTTCTACAAGAACCGTCGCTGCATCGG
CATAGCAGCTACTCACTTGCAAAACAAGCGTCTGTATCCGATGGTTTGTTCGACTGCGGCC
AAGAGCAGCATGAAACTGATCCGTTCACATTCAGCGCCCACCACATTACAGTACCTGTGCT
GTACGCAGCTGCGCAAAATGCTGCCCAACTGCGCAGACGCTCTGCGTGTGCTGCCGCTCC
CGCCGGGTCTGCGTCTGCTGCTGTCTAACCAGCTGGGATGGGTGCTGACCCTCGGCTGCG
CGGACGCCTGCGCAGACAACAGCGAGGACAAAGCCACACAAACAGAGGACAGCAACTA
CACAAGCCCCTTTCCTTCCCACAATCCCTCTGACACTGAACGTGTTTTTATTTCTAGTTCCT
TCTCTGATAACCCCGGTTCTTACTGTAGTAAAGATGACCCTCTTGATTCAGCCTCCATATTTC
CTGATTGTGCTTCCTGTCCAGATGATGCACATTATCAAAACTCCATAATGGATGTCGCCTGT
TACCTTGATCCCTCCTCCCCTTCTGACCTCTACTGTGGCTCCGCCCCGATCTCTGACCACAA
CTCCCTTCCTAACCCTGAAGGTCAGCACATATCTGCTTCCTGTCCTTGTTGTGACCCCTCGC
TCTCTGTTTCCAGTGATGGCCGTGAGGTAAATGACCTCTGTGCTGTGTATAAACCAGAACA
CACCGGTTCCTGCGTTCTCTGCCCGTCCTACACTCCAACAGGCCACACCTCCTTTTGGTCT
GACTCCGCCTCTGAGAGTGACTCGGACGACTTCTCCTCGGATCGCAAGACATGCAAGAGG
AAACGCTGCCGCTGGACATGA

>Zinc finger FYVE domain-containing protein 9, CI01000051_07283654
ATGAGGCAAGCAGTAACCGCCACTCTGTCTGTGTGGGGTGAGAGGCGTTTCGGGTACTCC
ACCCTCAAGGACTCCAGCGAGTCTCTGTTAGACGAGCGCAAGCTGAGGCCTGGGCTGAA
AGAGGACTCTGTCATTGAAGAAAAAGAGCGGGCAGAGAATGAGCAGGAGCAGCTGGAG
GCTGTGCTGGGACTTCCATCGTCCCCAGTGGAGGACACTCAGCAAACAGGCAGCACGGA
GCTTTCAGACTCTACGCTGGAGTCATGCCAGGACCATAAGAAGAGTGGTGGGCTGGAAGC
ATCACTACTGTTTGGCCCTAAGAACACTGACTCCGCGGGGAGGAAGGGTTCTCTTGGAGC

TTTGCCTGTGCAGCGCTGCAGTTCCCTGGGCACCACGGCCCCTCAGTGGGTCCCTGATTCT
CAGGCACCAGCATGCATGAAATGTGGATCCAAATTCTCTTTCACCAAAAGACGTCATCACT
GCAGAGCTTGTGGGAAGGTTTTTTGTGTGGTTTGCTGTGACCTGAGGTTTAAATTGACTCA
TCTTGGTGGGAAAGAGGGGCGTGTCTGTGTCACCTGTCATTCAACTTTGATTAACCGGACA
CTTCGGAAAGACCAGAAGAAGGTTTGGTTTGCAGATAATCTCCTTCCCCAGTCTGAGAGT
AGAAGTGCCAACAGCTCTCCTATTCACAGGAGTGCGTCCCACACACAGAGGGCTGAGCTC
GCTCAGGCGGAAGTGACTGACCTTAGAGCAGGAGATGAAGTCAGGGGTCTTAGTGGTAAC
TCCTCAAAACCCACTCCACCAAAGGACCTTCCTCCTATTCTGACATCAACAGGGGTCAAA
GGAGACTATACTCTGGAGGAGAAGAGAACAGAGTCGTCACTCTTAGAGGAGTTGGAGAG
AGGTCTTGCAGAGCCTTTGGTCTTTGTGCTTAACGCAAACCTGCTGGCTGTGGTGAAGATT
GTCAGCTATGTGAACCGGCGGTGCTGGTGTGTTATGTCGAAGGGTATGCATGCTGTTGGAC
AGCCAGAGGTGGTCATTTTATTGCAGTGTTTGCCTGAGGAGAAGAGATTCCCAACGGATAT
TTTCAGTCATTTCATTCAGATCTACTGGGATGCCCAGACAGCAGGAAAGCTTCTGAATCAC
CTAAGTCACTCTTTGGTATCCCGGGGGTTTCTGGGTAATAATGAGCATGCAGGCTTTCTTTA
CGTGCGTCCGACATTCCAGTCTCTGGAGGGCCTGCCGTTACCTGTGCCTCCCTTCCTGTTC
GGGCTCCTCATGCTCCGTGCAGAGGCTCCATGGGCCAAAGCCTTTCCCTTGAGACTCATGC
TACGTTTAGGAGCCGAGTACAGATTCTATCCCTGTCCCTTGTTCAGTGTGCGCTTCAGAGA
GCCTCTCTTCGGACCCGTTAACAACAGTATCATGAGACTCTTAGTGCTGCTGAAGGCTCTG
AATAAGTCTAATGAGAGAGTGCTGGCCTTGGGCGCCTCCTTTAATGAGCAGGCCGACTCTC
ATCTCATTTGCGTGCAGACGGCAGATGGACAGTACCAGACCCAGGCCATCAGCATTCACA
GCCAACCACGCAGAGATGGTTTGATGGTGCAGGTAACCATGGAGACCATGGCTGAGTTAC
GCCGATCTCTACGGGACATGAAAGACTTCACAGTCACCTGTGGAAAAATCCACCCAGCAG
AGAGGCAGGAATATGTGCACATTCAGTGGCAGGACGAAGAGCCACGCTTTAATAAAGGCA
TTATCAGCCCCATTGATGGGAAGTCTATGGAATCTCTCACAAACATCAAGACCCATCAGCG
CTTAGAATACAAAGCCAATGGGAAGCTCATACGCTGGACAGAGGTGTTTTACTTGCTGAAA
GACCAACATCCAAATGGTCTTAATAACCACACCAGCCATAACCGTCTAACAGAACGTCTTG
CACGTGCGTTCTGCTTGGCACTGTGCCAGTCTCTTTGTTTGCTGAAAGAGGACGGCATGAC
CAAACTGGCACTGAGAGTAATTCTGGATGGGCAGAAGATGGAGTTTTTGGCAGGCAGTAA
CGGTCAGCGCCTTCCAGCACCGTACCATGACTGTCTTAACCAATCATTAACATCCGTGGTG
CAGAGTAACTCTCAGTACCAGGGTCTCGCCGCTTGCCAGCTGGAGCTCATCTTCTATATTCT
TGAGGATACGGCATAG

>Major facilitator superfamily domain-containing protein 2A-B, CI01000054_08112291
ATGGCAAGAGGGGAAGGCGCAGAGCAGTACTCTAATGCCAATCTTCTGCAAAAACCCAGC
CCAGATGGAATCAAATTGGCAGCAAAGCGTGAATCAAAAGGCCGGTTGTCGGTGTGCAAC
AAGCTGTGTTATGCTGTTGGAGGAGCACCATATCAGATAACAGGATGTGCAATCGGCTTCT
TCCTTCAGATCTACCTGCTGGATGTTGCTTTGTTGGACCCATTTTATGCCTCCATCATCTTGT
TTGTGGGTCGAGCGTGGGATGCTATCACGGACCCAACTGTGGGTTTTCTGGTCAGCCGGA
GTCCCTGGACTAGATTTGGACGAATGATGCCCTGGCTCAGGCGGAAGAAATTCCTTTTTCG
TTCGTGCTGCATGCCTTGGGGGCTGGGAGATATATGCAAATTGCGGCTCATGAGTGGTCCA
ACCCACAACACACCACCTCTGTCATCCAATCAGAATGAAGGAAGGCAGACCAGCACTGCC
TCAAAGTCTAAAGGCGGCATACAGAGCGCTGTGGGGATCATTCTCTCTACCCCATTTGCTG
TGCTGTGCTATTTCCTCATCTGGTACGTGCCGTCTGTTGAGCAAGGCAAGGTTGTCTGGTA
CCTGATCTTCTACTGCTCCTTCCAAACATTACAGACATGCTTCCATGTACCGTACTCTGCTC
TTACCATGTTCATCAGCACTGAACAAAAAGAAAGGGATTCGGCCACAGCCTACCGAATGA
CTGTTGAGGTTTTGGGCACACTCATTGGCACTGCTGTTCAAGGTCAGATTGTGGGAATGGC
TAACGCTCCCTGTATCAGTTCTGGAGAGGATCTGAACTCCACAGATCTACAGGTGACTCCC
GAGGTCAACATCACCGAGCCTCATGTTTCACTAGAGCACCTGAGAAATGCTTATATGATCG
CGTCTGGTGTCATCTGCTCCATTTATGTCATCTGTGCCGTGGTCTTGTTCCTTGGTGTGAAA
GAACAAAAAGATGTTCCCGGGGCTTTTCTACACTGCTCAGGTTACTTTGAGTACTTTAAGC
AGAGCTCAGCTGGTCCCAGGGAAATGCCAATGAATAAGCTTAGCAGGGTGATTAGCTTTC
ACAGCACAGAGAGGTGTCTAAATGTGGCGCATGAGACCTGCAGGGTTAGGTCTGAGCCCA
TGTCATTTTTCCAGGGCATTCGTATGGTAATGGGCCACGGTCCATATGCCAAACTGGTCATG

GGCTTCCTCTTCACCTCTCTGGCCTTTATGTTTCTAGAGGGAAACTTTGCACTGTTCTGTAG
CTACACCCTGGGCTTTAGAAACGACTTCCAAAACATCCTGCTTGTCATTATGCTCTCTGCCA
CACTTGCCATCCCATTTTGGCAATGGTTCCTCACGAAATTTGGCAAGAAGACTGCTGTGTAT
ATTGGGACTACATCTGTGGTGCCTTTCCTGATCTCTGTAGTCTTGGTGCCAAGCAGTCTTAT
TGTGACGTACGTTGCATCTTTTGCTGCTGGAGTCAGCGTTGCAGCGGCCTTCCTCCTGCCC
TGGTCCATGCTTCCCGACGTTGTGGATGACTTTAAAGTCCAAAATCCTGAATCTCAGGGCC
ATGAAGCCATATTCTACTCCTTCTATGTGTTCTTCACCAAGTTTGCGTCTGGAGTCTCCTTG
GGGGTCTCAACTCTTAGTCTTGATTTTGCCGGCTATGTGACGAGAGGTTGCACGCAACCCG
CCGAGGTCGATTTAACCCTGAAGATCCTGGTGTCTGCTGCTCCAATAATTTTGATCATTATC
GGGCTGCTAATATTCATCTCCTATCCCATTGACGAGGAAAAGCGACAAGGCAACCGCAAAC
TTCTTAATGAACAACGAGAAAATGAGATCGACTCAGAAGCAGACTCCACTGAGAACAATG
TGGTTTAA

>Myosin heavy chain, fast skeletal muscle, CI01105482_00000540
CTGCTGGAGAAGTCTAGAGTGACATTCCAGCTTCCAGATGAGAGAGGCTACCACATCTTCT
ACCAGATGATGACCAACCACAAGCCTGAGCTGATTGAAATGACGCTCATCACCACCAACC
CCTATGACTTCCCCATGTGCAGTCAGGGTCAGATCACAGTGGCCAGCATTAATGATAAAGA
GGAGCTGGTTGCTACTGATACTGCTATTGACATTTTGGGCTTTAGTAATGAGGAGAAAATG
GGCATCTACAAGTTCACTGGAGCTGTGCTTCATCATGGTAACATGAAGTTCAAGCAGAAGC
AGCGTGAGGAGCAGGCTGAGCCTGATGGCACAGAG

>Ankyrin repeat and SOCS box protein 2, CI01000325_05750106
ATGGCAGTAGCCAGAGCAACAATGGCTGCTTCAAGGACAACAAATGAAGACCTAGAGGA
CTATAGTGCTTATGTACACATGTCTGAACAACAGCTGCTCCAGTTAGCTATTGAGCGAAGTC
TAGCTGATACAAATTTAACCCCATGGCAGGACCGACAACTGCATACCCACACAGAGCCAG
CTGGCCCTACACAAAGACCACAATTCATCCCTAACTCGGCAAACCCCCCCAATGAGAATAA
GTGTGCACAAATCAACAGAGATTGTCAAGATCATTACTTCAGTAAGGACAAAGACAAGGT
GATTGCCTGGACAAGGTACAATGGACACCTGCAGGTCACAGTGGAGCCTATGAATGATATA
GACCCTTTTCTCTCAGCCATTTGGAGAGGAGATGCAAAGGCTCTACGACACATCGTTCATT
TCAAATCTAAAAATCTCGATGAACCCAATAAAGAAGGCTGGATACCGCTTCATGAGTCTGC
ATACTATGGCCATCTCGAGTGCCTTAAGATTTTGCTCAGAGCCAAGCCAGATACAATCAAC
AAACGAACACACACAGAAGTCAGACACCGCTTTTTCTGGCTGTGGGTCGCAAAAATGTTTCT
TGTGTGAAGCATCTCTTGGAGCATGGAGCTGATCCTAACCTTGCAAATAACCAATGGGAGA
CACCACTGCACAGAGCATGCGAGAAACCTAATGAAGAGATTGTGGAGCTTCTTTTGAAAT
CCGGAGCTTCACCAATCAGAGCCTGTATTCAAGGCGGGACCCCACTACATGAAGCAGTGA
GGAACAAAAACTTAGAGATTTGTAAGATGTTGGTACAAGCAGGAGCAAAGCTGTGTGCCA
AAAATATTTATGGCATTGACAGTCTGTTTACGGCTGCTCAGTGTGATGCTGTTGATGTGCTT
AACTACCTAATATTGAAAGGAGGTAATATTAATACCCAGGCAAATGATAATGCATCCGCATT
GTTTGAGGCTTCTAAGAATGGTCATGATGAAGTTGTTGAGATCCTCTTGACGAAAAGAGCT
GATGTTAACAAAGCCGACAAAAGTGGATTACTTCCTATTCATGTTGCAGCAAAGAATGGTC
ATGAGAGCATTGTTGCCATGCTAATCCCTAGAACTAACATGGTCAAAGTGAAATACTCCGG
CATCAGTCCTCTTCATTTTGCTGCAGAACAAAACAGAGATGATGTTCTAGAAACGTTGATT
GAGGCTGGATATGACGTCAATGCCATGCTGTCAAATGATTGGTCAAAAATGCATGAAGATC
GCCGCAGCACAGCTCTGTACTCGGCTGTGGCAAACAGGAACGTTGAGGCAGCTACTATGC
TGTTAGAGGCAGGTGCTGACCCCAACCTGGACATTTTCAACCCCCTGCTAGTGGCTGTGA
GGAAAGGAAGTATGGAAATTGTGGCCTTGCTGATAAAGCATGGTGCTAATGTCAATGCCCT
GCTGCCAACTCATCCCACCAGCTTCCCAGCGGTTTTGGTTTTCTGTGTGAGGTACACGTTG
ATGTTGAAGTATCTACTGGACAATGGCTGCGATGCACTATCATGTTTTAACTGTCAATATGG
CAGTAATCCTCATCCCCCTATAAAACTAAGACGAAATGGACGAGAAACAATATATTATTTAA
ATGATGAACCATCAGAACACTGTGTACAG

>Myosin-13, CI01105456_00000678
AAGAAACTGGCTCAGCGTCTGCAAGAGGCAGAGGAACAAATTGAGGCAGTGAACTCCAA

ATGTGCATCTCTGGAGAAGACCAAACAGAGACTCCAGGGTGAGGTGGAGGACCTCATGAT
TGATGTGGAGAGAGCCAATGCTTTGGCTGCTAACCTTGACAAGAAGCAGAGGAACTTTGA
CAAGGTCCTGGCAGAATGGAAGCAGAAATATGAGGAAGGTCAGGCAGAGCTGGAAGGTG
CCCAGAAAGAGGCTCGTTCACTCAGTACTGAACTGTTCAAGATGAAGAACTCATATGAGG
AGACTCTGGATCAGCTGGAGACACTCAAGAGAGAGAACAAGAATCTGCAGCAGGAGATT
TCAGATCTGACAGAGCAGTTAGGTGAGACT

>Myosin heavy chain, fast skeletal muscle, CI01105408_00000861
ATGATTGATGTGGAGAGAGCCAATGCTTTGGCTGCTAACCTTGACAAGAAGCAGAGGAAT
TTTGACAAGGTCCTGGCAGAATGGAAGCAGAAATATGAGGAAGGTCAGGCAGAGCTGGA
AGGTGCCCAGAAAGAGGCTCGTTCACTCAGTACTGAACTGTTTAAGATGAAGAACTCCTA
TGAGGAGACTCTGGATCACCTGGAGACCCTCAAGAGAGAGAACAAGAATCTGCAGCAGG
AGATCTCAGATCTGACAGAGCAGTTAGGCGAGACTGGTAAGAGCATCCATGAGCTGGAAA
AGGCCAAGAAGACAGTGGAGACTGAGAAGGCAGAGATTCAGACTGCCCTGGAGGAGGCT
GAACACCGTGGTCCTGTGAGGCACGACATTTCCTTCCACTATATGTCACCACATGTAGCGG
AATGA

>Parvalbumin beta, CI01000351_01706079
GTTTGCTGCTCTTGTAAAGGCTTAAATTTTCATCTGACCAAGACCCTTCCATATTCAAGGAA
CCAGAACTCACGACAGCTCACACCTTTCTCAGCTGAACTTTTTTATATGATCTTATTTATGA
CTGACTTTGGACAGTTTACTCAGTTTCGAGTATGTGTCTGGATAAGGGATAAATATAGTACA
GCGTGTCTGCTGGGCGCATGTCAAGCCTCCAGCGGGTGTCTATTACATTATGATTCATATTT
GGAGTGGCTGAAGGTATATAAGGGGGAGATCAGGGGCCCGGAGATCACTGCAGTTCACAG
CTCGAGTCTTCTGAAACTACCTTCAGCACCAAAAGACAAAAGAGCTGCTGACTCCTTCAA
CCACAAGGCTTTCTTCGCCAAGGTCGGTCTGAGTGCCAAGTCCGGCGATGATGTCAAGAA
GGCTTTCGCAATCATCGATCAGGACAAGAGTGGCTTCATTGAGGAGGATGAACTCAAACT
GTTCCTGCAGAACTTCAAGGCTGACGCCAGGGCACTGACTGACGCCGAGACCAAGATTTT
CCTTAAGGCTGGAGATTCTGACGGTGACGGCAAGATCGGAGTTGATGAGTTCGCTGCCTT
GGTTAAGGCATAA

>Myosin heavy chain, fast skeletal muscle, CI01105431_00000857
TACAAGGTGCTGAATGCCAGTGTTATCCCAGAGGGACAGTTTATTGATAACAAGAAGGCCT
GTGAGAAACTCCTGGGATCCATCGACGTTGACCATGACCAGTACAGATTTGGACACACAA
AGGTGTTCTTCAAAGCTGGTCTTCTGGGTACTCTTGAGGAGATGCGTGATGAGAAACTGG
CTGCTCTGGTCACAATGACTCAGGCTCTCTGCCGTGGCTACGTGATGAGGAGGGAGTTTGT
GAAGATGATGGAAAGGAGGGAGTCCATTTACACCATCCAATACAACATCCGGTCATTCATG
AATGTCAAACACTGGCCATGGATGAAGGTTTATTACAAGATTAAGCCTCTGCTGAAGAGTG
CCGAGACTGAGAAGGAGCTGGCAACCATGAAAGAGGATTTCACAAAATGCAAAGAAGAT
CTTGCCAAGGCTGAAGCCAAAAAGAAAGAGCTTGAAGAGAAGATGGTAGCACTGCTGCA
AGAGAAAAATGATCTGCAGCTGCAAGTAGCATCTGAAGCTGAGAATCTCTCAGATGCTGA
GGAG

>Cytosolic phospholipase A2 gamma, CI01113241_00000638
ATCGATGAACACACTCTCACAGAGCAGTGGAACCAGCAGAGTAAAGACCCGTTTCCCATC
TACACAGTGATCGACAAGCAGTGCAAACAGAAAAAGGAAGCAGATCCCTGGTTCGAGTT
CACTCCACAAGAGGCAGGTTATTCCCTCATTGGAGCCTTTATGAGAGTCTCCAACTTTGGC
AGTCAGTTTAAAAAAGGCTCTATGATGAACGAGCAGCCTGAAATTGACATGCTGTACCTGC
AA

>Growth/differentiation factor 15, CI01000004_07830772
ATGCAGAACTCAGTAGCACAGTGCTTTTTCCTTTCTGTTGGGGCTGTTATTTTTAGCTCTTG
TCTGAGAGAGTCTACAGCTCAACTAGATCATGAGGAAACATACACGGGCCTGCAGCTTGA
GGCTCTGAAGTCTATTATTCTGGAGTACCTGGGGATGGACGCACCACCCAGGCCTGGAGG

AAGAGCTTCTCACCAGGACTTGGTCAGGATGTACCGTCAGTATAGGAGAATTGGACAATTG
CTTAAAGGGAATTCCAGTGAAGAACAAGAGCTTCAGCCTGCAAGAAGAGCCTCTACGGTG
CTCTTTCCCACGACAGTACAGCCTCTGAATTCCAGCATGGATTCAAGGCAGCAGTGGTTCA
GAGCTGCTTTCCAGAAGAACGCACACATCAAAACTGGAGTCAACCTTAAACGCGCAAGG
CTGCAAATTAAGAGACCACGCTTGGATAAAATTACACCCGGCCAGCCATGGCTATCAAAAG
ACATTGTGGTTAGAATACATAAACCTCTTAGTTTTCATAAAGAGACTGTAATTCGTGCAAGG
CACTTGAACTCTCGGGTTGTCACGTTGGATTTAACAGTTGCGGTTGAGAAGTGGCTTAAGG
ACACAAGTGCTGAGCTTTTAGTTGTTGAAATTTGCCTTCTCAAAAAACAAGAAGTGGGTA
CACAATCCATGCCTCTGCTTGTTTTACAACTTGAACAAGCAGTGAGGAGAAGCAGGAGAG
CTCTCTCTACTAAGGAGGAAAGTGTTGAGGATGAAGGTCACTGCAGGCGAAAGTCATTAA
ATGTTTCCTTCAAAGAGATCGGCTGGTCAGACTGGGTTATCGCTCCTTCGGGCTATACTATG
CACTACTGTGAGGGTTCCTGTCCGCATAGTTATAAAGCTGCCAGTATGCATACTCTAGTTAA
GTCTCGTCTGCATCTTATGTCCAAAGGAACAACACCCGGGCCTTGCTGCGTTCCAGCTGCC
TATGAGCCAATGGTTCTCATGCACTATGACAGTCGTGGAAAGCTGAAACTCACACCTTTCA
ACGATTTGATAGTTAGTAAATGCCACTGTGCATGA

>Insulin-like growth factor-binding protein 4,CI01000051_01787612
ATGAACAGACTGCTTCTGAACTTTTTCTGGGTGGCAGCATTCAGCGCACTCCTTTCAGCGC
CGGGGCTCCGGGCTTCCCCAGTGGTTGGGCAGGAACCTATCCGCTGCGCCCCGTGCTCCC
CGGAGCGGCTGGCTGAGTGTCCTGCGGTGGATGCCGGCTGTGAGGAGGTGCTTCGAGAG
CCGGGCTGCGGCTGCTGCCTCGCCTGCGCGTTGAAGAGAGGTGACCCGTGCGGGATCTAC
ACTGCGCCCTGCGGCTCAGGGCTCCGCTGCTTGCCGAAACCCGGAGAAGCCCGACCCCTG
CACGCACTCACCCGAGGACAGGCGGTGTGCACTGAGACCCCCGAGCCTGATCAGAGCCA
AAGCGACACAACGCCAGGTGAAAATATGCGCTCTGATTTCATCATTACCGAAATGTTAAAA
AACCCATTTAAAAAGAGACTCCGGTACCATGTGCGGCCACAAGAGGGCAGTAGAAGGTTT
CTTGAAATTATGACGGCCCTTGACAAGATCACTAAATCACAACAGAAACTGGGAGACAAA
ATGACCAGATTCTACCTTCCAAATTGTGACAAACACGGTCTATACAAAGTCAAACAGTGTG
AATCGTCTCTGGATGGTCAGAGGGGGAAGTGTTGGTGTGTGTCATCCTGGAATGGGAAGA
AGATTCCTGGATCAAGTGACCTGCCAGCAGATGCCGAGTGTCCCGAGGAACTCAACCACT
GA

> Ferritin, middle subunit, CI01000069_03626925
ATGGATTCTCAGATTCGCCAGAACTACGACCGCGACTGCGAGGCTTTGATCAACAAGATG
GTGAATTTGGAGCTTTACGCTGGCTACACTTACACTTCCATGGCTCACTATTTTAAACGGGA
TGATGTGGCTCTTCCTGGTTTTGCCAAGTTCTTCAAGAAGAACAGTGAGGAGGAGCGCGA
GCATGCCGAGAAATTCATGGAGTTCCAGAACAAGAGGGGCGGACGCATTGTCCTTCAGGA
CGTAAAGAAACCTGAGCGCGATGAGTGGGACAATGGGCTGGTTGCTATGCAGTGCGCTCT
TCAGCTGGAGAAGACCGTTAACCAGGCTCTGCTGGACCTGCATAAGGCTGCATCTGAGAA
GAGAGACCCTCATCTGTGTGACTTCCTGGAGACTCACTACCTGAATGAGCAGGTTGAGGC
CATCAAGAAGCTTGGTGACCACATTACCAACCTTTCCAAGATGGATGCTGGCAACAACAG
GATGGCGGAGTACTTGTTTGACAAGCACACCCTGGATGGAGACAGCTAA

>Glucokinase, CI01000053_05710574
ATGCCGTGTCTTACTTCAGCTCGTAGGCAGAGGATACCGAGTGACTTTGAGTCAGTACTGG
AGAGAATACTCATGGTGGACCAAATTCTGTCTGAATTTCTGCTGAGTAAAGAAGAGCTAGA
GGAAGTGATGAGGAGAATGCGGAGAGAGATGGAGAGGGGACTGCGTGTGGAGACACATG
ATGAAGCCTGTGTCAAAATGCTGCCCACTTATGTGCGCTCCACTCCTGAAGGATCTGAGGT
GGGTGATTTCCTGGCACTGGATCTTGGAGGGACGAACTTTCGAGTGATGCTGGTGAAAGT
GGGTGAGGATGAAGAGCGAGGCTGGAAGGTGGAGACAAAGCATCACATGTACTCCATCCC
TGAAGACGCAATGACCGGCACAGCTGAAATGTTGTTTGACTACATTGCCGGGTGCATATCT
GACTTCCTGGACAAACATAATCTGAAACATAAGAAGCTTCCACTGGGATTCACCTTCTCTT
TTCCAGTCCGTCATGAGGATTTGGATAAGGGCATTCTGCTTAACTGGACTAAAGGCTTCAA
GGCGTCTGGCGCTGAAGGCAATAACGTTGTGGGTCTTCTGAGAGATGCCATTAAAAGGAG

AGGGGACTTTGAAATGGATGTGGTTGCCATGGTGAATGATACAGTAGCCACCATGATTTCA
TGCTACTATGAAGACCGCAGCTGTGAAGTTGGCATGATAGTAGGTACAGGCTGTAATGCGT
GTTATATGGAGGAGATGCGTAAGGTGGAGCTGGTTGAAGGAGAGGAGGGGAGGATGTGTG
TGAACACGGAGTGGGGAGCGTTTGGGGACAACGGTGAACTGGAGGACTTCCGCCTGGAA
TACGATCGTGTTGTTGATGAGACGTCACTGAACCCTGGACATCAGCTGTATGAGAAACTGA
TTGGTGGGAAGTATATGGGGGAGCTTGTGCGTCTTGTGCTGCTAAAACTAGTGAATGAAGA
TCTGCTGTTTAACGGCGAAGCCTCAGACCTGCTGAAAACACGTGGAGCGTTTGAAACTCG
CTTTGTCTCCCAGATTGAGAGTGACACAGGGGACAGAAAGCAGATCTATAACATCTTAAGT
TCACTGGGAATCTTGCCGTCCGAGCTGGACTGTGACATTGTGCGTCTGGCATGTGAGAGTG
TGTCTACGCGAGCCGCTCACATGTGCGGGGCTGGACTCGCTGGCGTCATCAACCAAATGA
GGGAGCGCCGTTGCCAAGAGGAACTGAAGATCACTGTGGGAGTCGATGGCTCTGTCTACA
AACTACACCCTCGTTTCAAGGAGAGGTTCCATAAGCTTGTGCGGGAAATGACTCCTCACTG
CGAAATTACCTTCATCCAATCAGAAGAAGGGAGCGGTCGGGGCGCGGCTCTCATTTCTGCT
GTGGCGTGCAAGATGGCCGCCTGCATGCTGACACCATGA

>Protein-glutamine gamma-glutamyltransferase, CI01000001_00213625
ATTTTTGTTGACAGTTTTGCTGATGACCGTTATGAAACACAGAATGAAGCTGTTAACACAC
CTGTGTTTAAAGACTTGCAGAAAGATCTAGTTTGGACAAGGAAATACTGTAGGCACTTCAG
TTACTCATGTGAATGGGTTGGGAATGGAGATGTGAATATATATACCGGGCTACAGCAGAGA
AAGGTGTCACAGCACAAGAAGCCGTGGTTCCAGCCAGACTTACTTTGCTTCAGTAATTTG
AGTTCACTAAGAACTTTTACTCTCTTCATATTTAACATCACTCCGGGAGAAAGAACAGCAA
ACATCATGTCTGTCTTTAAGTATGGCCAGTTCAACAACTTCTGCCATATCGGCCGTTTCCCA
GGCATCAAACTCGGGCATTTTGACCATTACTGGGGAGGACATGGCTACGTGCCGTTTTACG
GTAACCCTTGTGTGGACCCTTGCGTGAAGCCTTTTGTCAATCCTTGTGTGACTCCTTGTGTT
AAACCTGTTGTCAATCCTTGCGTGACTCCTTGTGTGAAACCTGTTGTCAATCCTTGTGTGA
CTCCTTGTGTGAAACCTGTTGTCAATCCTTGTGTGACTCCTTGTGTGAAACCTGTTTTCAAT
CCTTGCGTGACTCCTTGTGTCAACCCTTGCGCTAACCCTTGCGCTAACCCTTGCGCCATCC
CTTGCGACTATGTGAGACCTCGCTGTGATTACGACTGCAAGGAAGTCTTCCTCAAGGATTG
TGCTGATGACTGCGTGCGTACCTGTGAAGATTTGGTTCTGCAGGTACGTTGCGTTGACCTG
ATGAAATCCTGGAAGTGTCAGAATCGTCAGGAGCATCACACCAGCTGTTTCCGTAATGATC
ACCTCATTGTCCGTAGAGGACAGTGTTTCCAGATGTGGATTGAGCTGTCCCGTCCATTCAA
TCCAAAATGTGACCAACTTCACCTGGAGCTGAGACTTGGTAATGTTCCATCAATTTGCAAT
GGCACTCTGGTGATTGTTCCATTAGTGGATGAATTCAAAAAAAATCGCTGGGAGGCCAAGA
TTGTCGAAAGGATGCAAAACCGAGTCAAACTGTGTGTGTACTCCCTTCCAACTGCCTGTAT
TGGACGATACAGCTTGACCGTTGTGACTTGCGGTCCAAGGGGCAGGGCCACATCATCATG
CAACCCTTGCAATGACATTTACATGCTGTTCAACCCCTGGTGTAAAGATGACTCCGTGTAC
CTGGATGACGAGGCCCAGAGAGTTGAGTATGTGCTGAATGACGTGGGCAAAATCTACTAT
GGAACTAGATGTCAGATCGGTTGCAGAACCTGGAACTTTGGTCAATTTGATGAGGGAATCT
TGGCTGCGTGTTTGTTCGTGTTGGAGAAGAGCGGTGGACCTTGCACTGGATGGAGCGACC
CCGTTAACGTGGCCAGAGTGGTATCTGCTATGATTAACGCCAACGATGACTGTGGTGTGTT
GGTGGGTAACTGGTCTAACTGCTATGGAGATGGAACTTCTCCCACATCCTGGTGCGGCAGC
AGTGCCATCCTGAAACAGTACCACAGATGTGGAGGAGTACCTGTCAAATATGGACAGAGC
TTGGCCTTTGCTGGAGTCACCAACACACTGTTGAGATGTTTGGGCATTCCCGCTCGTCCCG
TCACAAACTTCTGCTCTGCTCACGACACTGATGTGTCTATGACAGTAGATATTTATCTTGAT
GAGAACTATGATCTGATCGATCACCTGAACCGTGATTCAATCTGGAACTACCATGTGTGGA
ATGAGGCCTGGATGGCTCGTCCTGACCTCCCATGTGGTTTTGGAGGTTGGCAGGTGGTTGA
TTCCACTCCTCAGGAGACCAGCCAGGGCATCTTCCGCTGTGGCCCCACCTCTGTTGCTGCC
ATCCGCAGCGGGCAGGTCTTCCTCAAATATGACACTCCCTTCGTTTTTGCTGAGGTGAACA
GTGACGTAGTCTTCTGGCAGAGGACAGCATGTGGAACCTTCGGTGCTGTCTATGTTGACAA
GAACGCAGTAGGTCACTGCATCAGCACCAAGGCTGTCGGCTCAGACAAGCGTGTGGATAT
CACAAACTGCTACAAACACCCAGAAGGTTCAGAGGAGGAGCGTCGTGCTGTGGAGACAG
CCCTTCGTCACGGCTCCAGGAGATGCACATACCCACTGCCCTGCGCTGAGGATGTTGTCTG
TGATGTCACCATGAAGGGTGATGGACTGTGTGTGGGCAAGGAAGCCGTGCTCTGCATCGC

TTTGAAGAACAAATGCAACTCACCACGTAGCGTCACCCTCCACAGCCAGGTGTCGGCCAC
CTACTACACCGGAGTCCACAAGGCCTTGGTGAAGAAGGACCAGACGTGCTTTGAGCTCAA
GGCCTCTGAATCCAAGGTTCTGGAGTGGGCTCTGCGATATGAGGATTACAAGAATCACCTT
GTGGATCATTCAACAATGATGCTCACTGTTGCTGGACAGGTCACTCAGACTAAGCAGGTTG
TGGCCAAACGCTTCAACTTCAGGATGTGCACACCAGATCTGGTTATTACTCCTGGATGTGA
CTGTGTGGTGGGCAAAGAAGTGCCGATCAAGATCGCATTCCAGAACCCACTGCCCTGCGT
TCTGAAGAACGCCATATTCCGCATCGAGGGACTGGGACTGAAGCACTGCAGATCCATTAAC
TATGGTGACATTGCAGGCCTGGCCACGGTGAACCTGACCGAGACCTTTATTCCCAAGTGTC
ATGGCCCTTACAAAATTCTGGCATCTCTTGATTGTCCTCAGCTCACTCAGGTTCATGGATTC
ACCAACGTTGTGGTCAAAGAACACTGA

>Hepcidin-1, CI01000086_00632087
ATGAAGTGCGCACACGTGGCTCTCGCTGCTGCAGTCGTCCTCGCATGCGTCTGCATCCTTC
AGACCGCAGCCGTTCCGTTCGTACAGGTGAGTTTCTGCTGCAGGAGCAGGATGAGCATCA
AATGGAGATTGAAACACCACAGCAGAACGAACACTCGACAGAAACAACAGAAACACAG
GGACAAACAAATCCCCTGGCATTTTTCAGGACAAAACGTCAAAGCCATCTTTCCCTGTGC
AGATACTGCTGCAACTGCTGTCGTAACAAAGGCTGTGGATATTGCTGTAAATTCTGATCACA
TGGGCATTTGA

>Myosin heavy chain, fast skeletal muscle, CI01105471_00000597
ATGAGTACGGACGCAGAGATGGCCGTTTATGGCAAGGCTGCCATTTACCTTCGTAAGCCTG
AGAAAGAGAGAATTGAGGCTCAGAACAAACCATTTGATGCCAAGACTGCCTGCTATGTGG
CTGATGTCAAAGAGTTGTACCTCAAGGGAACAATCAAGAGCAAAGATGGTGGCAAAGTCA
CAGTTGTTTTGCTTGACACTAAGGAGGAGAGAGTTGCTAAGGAGGAAGATGTCCACCCAA
TGAATCCTCCCAAGTTTGACAAGATTGAGGACATGGCCATGATGACCCATCTCAATGAACC
CTCTGTGCTGTATAACCTCAAAGAGCGTTATGCTGCATGGATGATCTATCAGTCTGACCACT
TCTCTGCTCATTTCAGACCTACTCTGGCCTCTTCTGTGCAACTGTGA

>Calcium-independent phospholipase A2, CI01000001_04971921
CATCCGGTCCTCATTCAGATCATCTGCTTGGACTCTGTTGGTACTTCACTGATGGGTTGGTA
TCTTGACAAAACACAGGTCACTTACCTGGGGTTAGGGCGGCTCTGGAGTATCTGCTCCTCA
CCTCAATCTACAAGGAGAATTCCTCTCAAATCTCATAGATCAATAAAAACTTTGAGACTTTT
TGGAGCATCTAGCAGTAATATGAAATATAAGAATGGATCAGGCCTTCAAATGTACCATTTAT
ACTCAACCTCCAGCAGAGGGACATTCAAAGCAGAAGCACCCATTGGAGTTTACCAGAAA
AGCCGGACTACTTTTCGGCTGCACTTAATTGGACTAAGATTAGGAGAGTCTTTTAATCGTCT
CTCCCGACACATTAACTTGTATTTTAAGCAGAAACAGGTCTCTGTGATAGATTATGGGCAG
AATGAGTTGATACCTGCAGTGCAAGAACCACTTACCAGAATACAGAGACGAGTTCAGAGA
GAGAGACGCTCTGGAGAGTCCAGAGATTTTATAGGGATGCCTTCCATTGAGCAGGTCCATC
AAACATCCTCAAATCAGCCTTTGACCTCGGCCTATCCTGGACTACAGCTGTTCCACATCAG
CTCCTTGGCAAACAGATTTGGGGAGACTTACAGCTATGTGGCCAGTCACATTAACTCTGTA
TTCTCTCGAAATCCAGCAAAGCAAATCCATGTGGAGGTATCACCAGATGATGGGTTCAGAA
GGTCTAGAAGCAGAAAGCGAAGAGTTATGGGTAATAAAAGTGTTCTTTGTAGGGAAACTG
AGCAAGCTCAGCGAAACATTAGTTTGGGTACAAGACCAGAGATTGATCACAATGTATCTAG
TTCCTGGGAGGAAGGATACCTTCATTTTGCCAGACACATCAATCGATACTTTGGTGCAAAA
GTAGCAGATACTGTTGAGGAATCATCTCAGTTGTCACATACTGAGACAAACATCCTGAAGA
CTTCAGTCTCACTTGACCCTCTAAACAAAACTAAAGACCCTACTTCACTTCTTCAGCCTAA
ATCCCCAGGCTTGTTCCACATGAGCAGCCTCACCACACGCTTTGGGGAAAATTACACTTAC
ATGGCCAACCACATTAATCGGTACTTCAAAGGATCTGCAGCTTCGGAGAATGAGGAGGTG
GGTGGAGAGCTATACACAGAACAATACAGAGGTTCATCTGAGCACATGGCGAGCCAGGAA
AAACCAGTACCCTTCTTTGAGTGCCTGTTGAAACCCTCAACTTTACCTGGCTTTGTGGGCA
GCTACCTAGGAATAAGTTCTAGCAGACGTAGTGACCATATAACTGTAACAAAGACCCCAGA
GGAAATGCTGGATAAAACGGTTTTAAGGAGGAGAAAGGCAGATGAGATGACAAGAGTTCT
ACTGAACAGGCTGGAAGAGGCTAGCATACAGTCATCCATCACTTCTTGTGTGGAGGAACT

GAACACTCATCTCATTCAGCACCCAGCATGCAAAGCTGTCGTGTGGCAGGAAAAAGCAGC
ACTGCATCTCCTTAAACGACGCCGGATCTTTTGGATGAATGAAAAACTTCAAGAAGCCATT
AGGGAAACTCTTGCTCTGATTGGTTATGTGGATCCAGTCAAAGGTTGTGGATCAGAGTGC
TTTCCATTGACGGAGGTGGAACCAAAGGGCTAGTTCCACTACATGTGCTAAAACAGCTGG
AGGCTCAAACAGGAAAGCGAGTGTATCAACTTTTTGACTATATATGTGGAGTCAGTACAGG
AGCAGTTTTGGCTTTCATGTTGGGTCTAGATCGTATCTCTTTAGATGAATGTGAAGAGATGT
ATCATCGTTTTGGGATGGACGTTTTTCGACAAAACCCCCTGGTTGGTACAGTGAAGATGGG
CTGGACACACTCCTACTACAACACGGAAACATGGGAGATGATCTTAAGTTCTCTACAGGTG
TCTGCTGTAAGTGCCGTAGTGAACTGGGGGAAAAGTCCGAAAGCATTTATCTTCCGCAACT
ATAACCATGCTCCAGGACGACTGAGCCGCTACGCCGGAGGATCAGGGTACCAGTTGTGGC
AGGCTGTCCGAGCCTCATCTGCTGCTCCAGGATATTTCCAGGAGTTCCCTCTCCATGGTGA
CATACACCAGGATGGTGGTCTTATCCTCAATAACCCCTGTGCTCTGGCTGTCCATGAAAGC
AAATTACTGTGGCCCAGTCAGCACTTCCAGTGTGTGCTGTCACTCGGAACTGGTCGGTATG
ACAACGCTAGAAGAGGGCCGGCAACCTCCACTAGTTTAAGAGCCAAAATCAGCAACCTGA
TCTGCAGTGCTACCGATACTGAGGGTGTTCACACTCTATTGGGTGACCTCTTATCACCGAA
CGTGTATTTTCGCTTTAACCCGATGCTGAGTTCCAACGTGACACTGGATGAAAGCAGGCCT
GGAGTGCTTCAACAGCTGCAGAAAGACACACAGCTGTACTTGGACCGGAACCAACCCAA
ACTGGAACGTCTGTGCGAAGTGCTGATGGCAGAGCGCACAGCAATATGGAAAACACGGG
ACTGGATCGGTGGGAAAGCGTGGGAGCTGCAGCAGCGCTGGGCTTGA

>Elongation of very long chain fatty acids protein 6, CI01000000_13586927
AAGAAGTCCTTCCTCTTCTCTGCGCTCTACGCTGCCTGCATACTTGGTGGGCGGCATGTAAT
GAAACAAAGGGAGAAGTTTGAGTTGAGGAAACCTTTAGTACTATGGTCTTTAACACTTGC
AGCCTTCAGTATATTTGGTGCCATCAGAACCGGAGGCTACATGATGAACATCTTGATGACC
AAAGGCCTAAAGCAGTCAGTGTGCGATCAGAGTTTCTACAACGGACCGGTCAGCAAGTTC
TGGGCCTATGCTTTTGTCCTCAGCAAGGCACCTGAACTAGGAGACACACTATTCATTGTGT
TACGCAAGCAAAAGCTGATCTTCCTGCACTGGTATCATCACATCACAGTGTTGCTCTACTCA
TGGTACTCCTACAAGGACATGGTGGCCGGCGGGGGCTGGTTCATGACCATGAACTATCTGG
TGCATGCCGTCATGTATTCTTATTACGCTCTTCGGGCCGCCGGCTTCAAAATCTCTCGCAAG
TTTGCCATGTTCATCACCCTGACGCAGATCACCCAAATGGTGATGGGCTGCGTGGTCAACT
ATCTGGTGTATTCGTGGATGCAGCAAGGCCAAGAGTGCCCGTCCCACGTGCAGAACATTGT
GTGGTCGTCCCTCATGTACCTCAGCTACTTTGTGCTCTTCTGCCAGTTCTTCTTCGAAGCCT
ACATCACCAAGACCAAATCCAATGCAGCCAGGAAAAGCCAATAA

>Glycine amidinotransferase, mitochondrial, CI01000166_00651860
ATGCTGCGAGTCAGATGCCTGAGAGGAGGAAGCCGCGGAGCAGAAGCCGCACATCTCATC
GGCGCTATGGTGGGGCGCGCGGTGTCGGGATGGGTGTCGCGCGCGTTCCGCAGCACCTCG
AGCTCCGCCGCAGCGCAGCTCCCGCTCACGGTGGACGAGCCGGTTACTGATCACGCGCCC
GAAGAGTGCCCCGTGTGCGCGTACAATGAGTGGGACCCGCTCGAGGAAGTGATTGTCGGG
CGAGCCGAGAACGCCTGCGTGCCCCCGTTCACCGTGGAGGTCAAGGCCAACACCTATGAG
AAATACTGGCCCTTCTACCAGCAGTACGGCGGACAGACATTCCCGAAGGACCACGTGAAG
AAAGCAGTTGCTGAAATCGAGGAAATGTGCAATATTCTGCAGCACGAGGGTGTTACAGTG
AGACGACCGGAACCGATCGACTGGTCATTAGAGTACAAAACCCCTGACTTCACCTCCACA
GGCATGTACGCAGCCATGCCGAGAGACATCCTGATCGTGGTGGGAAACGAGATCATCGAG
GCTCCGATGGCCTGGAGGGCCCGGTTCTTCGAGTACCGCGCGTATCGGCCCCTCATTAAGG
AGTACTTCAGGCGGGGAGCCAAGTGGACCACTGCACCCAAACCCACCATGAGTGACGAG
CTGTACGATCAGGTTACAAACTTCATGGGCATCGAGTGGATGAGACGTCACCTCTCCCCCA
CCTACAAGATTCACATCATCTCCTTCAAAGATCCTAACCCCATGCACATCGATGCCACCTTC
AACATTATCGGGCCGGGACTGGTGCTGTCCAACCCCGATCGGCCCTGCCGGCAGATCGAC
ATGTTCAAGAAAGCCGGTTGGACTGTGGTGACCCCTCCGACTCCTCTTATTCCAGACAATC
ATCCGTTGTGGATGTCATCCAAATGGCTGTCCATGAATGTCTTGATGCTGGATGAGAAACG
TGTGATGGTGGACGCTAATGAGATGACCATACAGAAAATGTTTGAAAATCTCGGTATTAAC
ACTATTAAAGTCAACATCCGCCACGCCAACTCCCTGGGCGGAGGCTTTCACTGCTGGACG

ACGGACGTGCGCCGCCGCGGATCCCTCCGGTCTTACTTCCTCTAG

>Troponin C, skeletal muscle, CI01000001_06305351
ATTATTTGGAAAACAAAAACAAATGACTCTCCAGAAGAACCAGGATGTCTCTATTATCAGA
TTAATACATTGTACTGTTCAAATCTGAAACTAACCGTTCGTAATGTAACCGCTGTCTCTCCA
AAGACTGACGCGCAACAGGAGGCCCGCTCATATCTGAGCGAGGAGATGCTTGCCGAGTTC
AAGGCTGCCTTCGACATGTTCGACACCGATGGTGGCGGTGATATCAGCACCAAGGAGTTG
GGTACCGTCATGAGGATGTTGGGTCAGAACCCAACCAGAGAGGAGCTGGAAGAAATCATC
GAGGAGGTCGATGAAGACGGCAGCGGATCCATCGACTTTGAGGAGTTCTTGGTCATGATG
GTGAGACTCCTAAAGGAGGACCAGGCTGGCAAGAGCGAGGAAGAGTTGGCCGAATGCTT
CCGCGTGCTGGACAAGAACGGTGATGGCTACATCGACAGAGAGGAGTTTGCTGAGATCAT
CCGCAGCACTGGTGAATCCATCTCAGAGGAAGAGATTGATGAGCTGCTTAAAGATGGAGA
CAAGAACAACGATGGCATGCTGGACTTTGATGCAGACAGACAGATAAACACCTGTGTGGG
GTTTGCCAAGGTTGGGCTCCCACCGACCCTAACGCAAGACCCAAGGAGCCAAGCTGTGGT
GTGGTTCACA

>Solute carrier organic anion transporter family member 1C1, CI01000066_02680009
ATGGACCCGCCAAACAAGGAGAGACGGTCACCGGAGAACGGCCCAAGGCCCTGTTGCTC
AAGCCTCAAGATGTTCCTCGTGGCCTTGTCCTTTGCCTACTTTGCCAAGGCATTATCCGGG
AGCTATATGAAAAGCACAATAACTCAGCTGGAAAGACGCTTCGACATCCCCAGTTACTTAA
TAGGTGTCATTGATGGCAGCTTTGAAATAGACAAAGAGGTCAATCATGTAGAAATCAGCGT
CATGTTCAGAGAGTTTGTGGAGCGGATGCTTCCATTCATGGGTGAATGTAACTTGTTAGTG
ATCGCATTTGTGAGTTACTTTGGTGCCAAACTTCACCGTCCGAAGATCATCGCCATCGGCT
GCCTGTTGATGTCACTAGGGACTTTTCTAATTGCCTTGCCCCATTTTATAATCGGACGCTAC
AAGTTTGAAACATCGATTCGATCGTCAGTGAACTCGACCAGTAACCTCTCTCCATGTCCAG
TGAGGTCCACAGGTCCAAGCACTTCAGTACCGAGCTCAGGTTGCGAACACGAGTCCAGGT
TGTCCATGTGGATCTATGTTTTCCTAGGGAATATCTTGCGTGGAATTGGCGAGACCCCAGTG
CAACCTCTAGGAATCTCATATATTGATGATCACGCTCTGGAGGAAAATGCAGCCTTCTACAT
TGGATGTGTCCAGACAATATCAGTCATCGGTCCAGTGTTTGGCTACCTGCTGGGGTCCCTC
TGTGCCAAGATATACGTGGACATTGGATTTGTCAACATGGAGAGCATTAGCATCACCCCAG
GAGATGCACGCTGGGTAGGCGCCTGGTGGCTGGGATACCTTATTGCAGGATTCATCACCCT
GCTCTCGGCGGTGCCGTTTTGGTTCTTGCCCAAGTCTTTGCCTCTGCCAGAGAGACGGCTT
GCCAAGTATTCTCCAGAGCGGACCAGCTTCATCAAAGACTCGCCCCTCCTGGAGCACAAG
TACCAGGCAGATGAGCCAACAAACTTCCTAGAAATGGCCAAAGACTTTTTGCCAACACTG
AGATCCCTTTTGGGGAACCCGGTCTACTTTTTGTATCTCTGCGTCACCATCATCCAGTTCAA
CTCTTTGATCGGCATGGTGACATACAAACCAAAATACATCGAGCAACATTACGGACAGTCA
GCGTCCAAGGCGAACTTCTTAATGGGAGTTATCAACATCCCAGCTGTAGCCTTGGGAATGT
TCTCGGGGGGTGTGATCATGAAGAAGTTCAAGCTGAGCATCATGGGAGCAGCCAAGTTTG
CACTGGGAACGTCTCTCCTGGGCTATTTCCTGTCCCTTTTCTTCTTCGCCATGGGATGTGAG
AATGCCAATGTGGCCGGCATCACCAGGTCGTACAACGGGACGGAAAGTCTTTACAAAGTG
GACAGTTCCCTCTTAGCGACGTGCAACGCAGGCTGTCAGTGTCCTGAAACAAACTGGGAC
CCCGTGTGCGGTGAGAACGGGCTCACCTACGTCTCCCCCTGCCTGGCTGGATGCCGGACG
TCCCACGGTTCTGGAATGGACACGGTGTTCAAGCAGTGCAGTTGTGTAGGAGTCGGGAAT
CAAACGGCCAGCGTGGGTCAATGCAACAACAGGGAGAACTGCCATCGAGTCTTCCCCTAC
TTCCTGGCTCTGTCTGTCATCACTTCCTTTATCATCTCACTGGGCGGAACGCCGGGATACAT
GCTGCTCATCAGGAAAAGCTTTAGCACCAAGGACTGTCAGCTTTCTCTCAAAGCTCAGTC
AGCTGACGCTGCTGGAATGCTCGGCCTCCCTTGGGCTAAATTAGCTGGAATTCCAGCTCCT
ATATACTTTGGGGCCATCATAGACACCACCTGTCTGAAATGGGGTCACAAGAAATGCGGTG
GAAGAGGAGCCTGTCGAATATACAACACGTCTGCCTACAGGATAGCATATCTGGGCCTGAC
GCTGGGCTTACGGACAGCTTCCTTCTTCCTGTGCGTGCTTGGGCTGGTGGTGTTACGACGG
CATGTTAAGAGGCAAGAGCGTGGCACGCTCGCAAACGGAGGCACGGGGGCCGCGGCCGG
AGAACTGCAGGCTCTCAGGAAAGAAGAAAACAACAGCTGTAACTCGGACCAATGCCCAC
GAACCACAGACTACGACCCAGAACGGGAGACGCGTCTGTGA

>Kelch domain-containing protein 1, CI01000060_00007071
CACTACTCAGTCAATCTCCATGATGGGACGTTTACCTGGAGGAAGGTCAATCATCGGTCAG
GATCTCCGCCGTCGCCGCGAGATAAACTGTCCTGTTGGGTTCATAAAGGAAGAATGATCTA
CTTTGGTGGATATGGTCACAAACTGCTGAGTGAAATCAATGATCCGAAGAGCTTCACTGTG
GATGAAGCATCAGGGGCCGAGGACATTTTCTGGGGATGGAACAATGAAACTCATGAATTT
GATCCGGACAGAAGCAGCTGGACTGAACCAAAGACCTCTGGCCGTTCTCCTGCGCCGCGA
GCCGCTCACGCTAGTGCCACGATCGGTAGTAAAGGTTATGTGTGTGGCGGAAGAATAAAG
GAAACTAGAACCAGTGATGTGTTTTGTCTGGATTTAAATTCATGGACATGGTCAGAAATCG
TCTCCTCCAGCGCCGCTCCGTCAGGTCGCTCGTGGCACACGCTCACGTCAGTGTCAGATTC
CTCTCTCTTCATGTTCGGTGGACTCAGTGTGGACTGCAGACCCTTGAGTATGACACAGAAA
CAACACTTCAGACAAAAATATAACTCAAACAAACCAAAGTATAAAGCTTGTTTATTCTCTT
CAGGTGAGGGTTGGATATTTAATCTGGAGACAAAAGGATGGACGAGGATGGAGCATCAAT
ACAAGGACAAACCGCGGCTTTGGCACAGCGCTTGTCAAGGAAGAGACTCGGATGTGATT
GTGTTTGGAGGAAGTCATGATTATATTCTTCTGGTGGACAAAGGCCACTGCAACGATGCGC
TTGTTTTCCAGACTCAGCCGTACCCGCTGATACGGTACTGATCACTGGATCCAACAGAAAT

> fructose-bisphosphate aldolase a,CI01000046_04355463
ATGACTCGCGTCCTGATTGGTCAAGCTACCGTCTTATTCAAGATGCCTCACGCATACCCGT
TCCTCACTCCAGAGCAGAAGAAGGAGCTGAGCGATATCGCTCTGAGGATTGTGGCCCCTG
GCAAAGGCATTCTTGCTGCAGATGAGTCTACAGGTAGCGTGGCGAAGCGGTTCCAGAGC
ATCAATGCTGAGAACACGGAGGAGAACAGGAGACTGTACCGCCAGCTGCTGTTTACCGC
AGACGACCGCGTCAAGCCCTGCATCGGTGGTGTCATCCTCTTCCATGAGACCCTCTACCA
GAAGGCAGATGATGGCAAACTTTTTTCCCAGCTCCTTAAGGAGAGGGGCATGGTAGTTGG
CATCAAAGTGGACAAGGGTGTGGTTCCCCTGGCTGGCACTAATGGAGAGACCACCACAC
AAGGTCTGGATGGGCTGTACGAGCGTTGTGCTCAGTATAAGAAGGATGGGGCAGACTTT
GCTAAATGGAGGTGTGTGCTGAAGATCACTTCCACAACTCCCTCTAGACTGGCCATCATT
GAGAACGCTAATGTGCTCGCTCGTTACGCTAGCATCTGCCAGATGCATGGCATTGTGCCT
ATCGTGGAGCCTGAAATTCTGCCTGATGGTGACCATGACCTGAAGAGGTGCCAGTATGTG
ACTGAGAAGGTTCTGGCTGCTGTGTACAAGGCTCTGTCTGACCACCATGTCTACTTGGAG
GGCACTCTGCTCAAACCCAACATGGTGACTGCCGGGCATTCCTGTTCCCAGAAGAACACC
CCCCAGGAGATCGCCATGGCAACTGTCACAGCTCTTCGCCGCACTGTTCCTCCTGCCGTG
CCTGGTATCACCTTCCTGTCTGGAGGCCAGAGTGAGGAGGAGGCCACACTCAACCTGAAC
GCCATGAACAAGTGTCCCCTGCATAGGCCCTGGGCTTTGACCTTCTCCTACGGTCGTGCC
CTGCAGGCCTCTGCCCTCAAAGCCTGGGGTGGCAAGAAGGAGAATGGCAAGGCCTGCCA
GGAGGAGTTCATAAAGAGAGCTCTTAACAACAGCCTGGCCTGTGTTGGGAAGTATGTGTC
CTCAGGAGATAAGGGTGCTGCTGCTGGAGAGTCTCTCTTTGTGGCCAACCATGCCTACTA
A

>apolipoprotein Eb,CI01000027_05063086
ATGAAAGGAGAAGTGCCCTTAAAAATTTCGAAAATAAACTATATTAATAGCGCATTTTAT
CCCCACCTGGTGCGAAAAGATGCGGACTTTGCACACTTGCTGCAGTCAGTGACGCGTGGG
ATGATGCGCGAGCCCCGAACAAAGGCGAAATCACGTGGCAGTGATTGTGAAGGCAGCGG
GTGTACTTGTCTGTCCATCAAAATGAGGTCTCTTGTGGTTATCTTTGTTCTGGCAGTTTTTA
CTGGCTGCCATGCTCGTAGCCTGTTCCAGGCTGATGCCCCTCAACCCAGGTGGGAGGAGA
TGGTGGACCGCTTCTGGCAGTATGTGTCCGAACTCAGCACACATGCTGACGGTGTGATGC
AGGACATCAAGGGCTCCCAGGTCAGCAGAGAGCTTGACACCCTAATTACTGACACCATG
GCTGAGCTGAGCTCATACAGTGACAGTCTTCAAACCCAGCTGACACCATACGCCTCTGAT
GCTGCTGGTCAGCTGAGCAAAGATCTCCAGCTCCTGGCTAGCAAACTGGAAACTGACATG
ACCGACGCTAAGGAACGCTCCACTCAGTACCTGCAAGAGCTGAAGACCATGGTGGAGCA
GAACGCAGATGATGTGAAGAGCCGTGTCAGCACCTACACACGCAAACTGAAGAAACGCC
TGAACAAGGACACCGAGGAGATCCGCACCACCGTGGCAACCTACCTGGGTGAGCTGCAG

TCTCGTGCTTCCCAAAATGCTGATGCCGCGAAGGACCGTTTAGAGCCATACCTCACCCAG
GCCAAGGATGGTGCCAGCCAGAAATTGGGTGCCATTAGCGAGCTGATGACGTCCCAGGC
ACGGGAGGTGAGCGAGCAGTTGGAGGTCCAGGCTGGAGCTCTGAAGGAGAAGCTGGAA
CAGACCGCTGAGGAGCTGCGCACCTCCCTAGAGGGCCGCATGGATGAGCTGACCAGCCT
CTTCGCCCCCTATTCCGAGAAGATCCGTGAGCAGCTGCAGATTGTCATGGATAAGATCAA
GGAGGCCTCAGCAGCTCTTCCCACTCAAGCTTAA

>eukaryotic translation elongation factor 1 alpha 1, like 2, CI01159237_00002439
ATTGGAACTGTGCCTGTCGGACGTGTTGAGACCGGAACTCTGAAGGCTGGTATGGTTGTG
ACCTTTGCCCCTGCCAACGTGACCACTGAGGTCAAGTCCGTTGAAATGCACCACGAGTCT
CTTGCTGAGGCTCTCCCCGGTGACAACGTTGGCTTCAACGTTAAGAACGTGTCCGTCAAG
GACATCCGTCGTGGTAACGTGGCTGGAGACAGCAAGAACGACCCACCCATGCAGGCTGC
CAACTTCACCGCTCAGGTCATCATCCTGAACCACCCTGGTCAGATCTCTCAAGGCTACGC
CCCAGTGCTGGACTGCCACACCGCTCACATTGCCTGCAAGTTTTCTGAACTCAAGGAGAA
AATTGACCGTCGTTCTGGCAAGAAGCTCGAGGACAACCCCAAGGCTCTTAAATCTGGAG
ATGCTGCCATTATTGTTATGATCCCTGGCAAGCCCATGTGTGTGGAGAGCTTCTCTCAGTA
CCCACCACTGGGTCGTTTTGCTGTGCGTGACATGAGGCAGACCGTCGCTGTTGGTGTCAT
CAAGGCTGTTGACAAGAAAGCCGCCACTGCCGGCAAGGTGACCAAGTCTGCTCAGAAGG
CAGCCAAAGTTAAATGA

>macrophage migration inhibitory factor, CI01000000_04081018
ATGCCGATGTTCGTGGTGAACACAAATGTAGCAAAGGACTCGGTTCCTGCTGAGCTGCTG
TCGGAGGCCACGCAGGAGCTCGCCAAAGCGATGGGCAAACCCGCGCAGTATATTGCCAT
ACACATCATCCCAGATCAGATGATGATGTTCGGGGGAAAAGGAGATCCGTGCGCGCTCT
GTTCTCTCACCAGCATCGGAAAGATCGGGGGCGCGCAAAATAAGCAATACTCAAAACTT
CTTATGGGCTTGCTCAACAAACACCTTGGCATTTCACCTGACAGGATCTATATAAATTTTG
TTGATATGGACGCAGCCAATGTGGCTTGGAACAGCACCACCTTCGGATAA

>elongation factor 1-alpha, CI01159234_00002070
TACGTCACCATCATTGATGCCCCTGGACACAGAGACTTCATCAAGAACATGATCACTGGT
ACTTCTCAGGCTGACTGTGCTGTGCTGATTGTCGCTGCCGGTGTTGGTGAGTTTGAGGCTG
GTATCTCCAAGAACGGACAGACCCGTGAGCATGCCCTGCTTGCCTACACTCTGGGAGTCA
AACAGCTTATTGTTGGAGTCAACAAAATGGACTCCACCGAGCCCCCCTACAGCCAGAAG
CGCTATGAGGAAATCACCAAGGAGGTCAGCGCCTACATCAAGAAGATCGGCTACAACCC
CGCCACCGTTGCCTTCGTTCCAATCTCTGGATGGCATGGAGACAACATGCTGGAGCCCAG
CACAAACATGACCTGGTTCAAGGGATGGAAGATTGAGAGGAAGGAAGGGGCTGCCAGTG
GTGTTACCCTTCTCGAAGCCCTGGACTCCATCCTGCCACCCACTCGGCCCACCGACAAGC
CTCTTCGTTTGCCTCTGCAGGATGTGTATAAGATTGGA

>pleckstrin and Sec7 domain containing 2, CI01000000_00523798
ATGAGTCAGGAGGGGCAGGATGACCCCCTCCACACAGGCACCCCACCTCCAGATATACC
CCCAGCACCTGAGGGAGAAGAGGATGCTGATGGCTCTAAGGCTAAAAATTTGGAGCCCT
GTAATGGACCAAAGACAGAGGACAAGCATCAAGGTGAAGCAGAAGACAGAGAAACAGA
GAGCAACAGAAATACACAGTCCAGTACAAAGGAGGATGAGAAGGTGACATTAGAAGAG
GAAGGTAGAAATGGTGAGATTGAGGGCAGTTGCTTGGATCCTGTAACAGAAAACGGTAA
CAGGGATATCTGTGAAGGTGAAGACACACAGTCGCTGCTGCCTCAGAGCCCAGAAAAGG
ACTTAACTAAGCCCCTGTGCTCTCTAAGGTTTGAGAACGGGACCGAAGATGACAGCGAA
GGGCAGGAGGATGAAGAGGAGATCCTGGAAGAGTCTTCAGTGCAGGGGAACCTAGATGC
CTTCAGTTCCACATTTGAGCTCTCCGTAGAGGAAGCGGATGCTGAGGTAGAGGACGAAG
AAGAAAAAGAGGAAAGTGAGACTCGAAATCAGGACAGTCTCAGCTCTACGTTTGAGAGC
ATTGTTGAGAAGGTCGCCAAGACCACGGCTGTGGAAGAGGAGGTGGAGGACGACAGAG
AGGAAGAGTATGACAGGACAAAAGAGAGCCAGGATGGTTTCAGCTCCACATTTGAGCGC
ATCGTCGAGTCGGAACTTCTGAGAGGCGGAACTTGCTACAGCAGTTTGGATTCTCTGGAC

GTGCTGTCGCTCACCGATGAGACGGACAGCTGCGTCAGCTTCGAGGCGCCACTCACCCCT
CTCATACAGCAGAGGGCCCTAATGGGCCCTGAACCTCCAGATCTGGAGCTGGAGACTGT
GCAGGAAGGGGCTGAGGGCGAGGCAGAGCTGGGCACAGAGACGGCAAGGGATGAAGCC
AGCTCTGCAAGTCCACTACGGATCACTATACCAGGAAGTAGGTCGGAAAATGTCTTGAGC
CAGCCGGCACAATGGAGCATCCCTAATGGATTCCATATTGAGTTACGTGGAAATCGAGA
GAGTGGAGCAACAATGCCAAACTCTATCAGCGATGCCAACCTCACAGATGTGCTGTGCG
ACTCAGACTCCGAGTTGGGCAGCGTGGACACGCTGGAGAGGGGTAGCACAGATACTCTT
GCCAATGGCTGCCGCATCGATGCAGATGCTGCCAAACGACTGGCCAAGCGACTGTTCTAT
CTGGAGGGCTTCAAGCGCTGTGACGTGGCACGACACCTGGGCAAGAATAATGAATTCAG
CCAGTTGGTGGCATCAGAGTACTTGAGCTTCTTTGACTTCACTGGGTTGTCATTAGACAG
AGCGCTGAGGAACTTCTTGAAGGCATTTCCTCTGATGGGAGAGACGCAGGAGAGAGAAC
GTGTGCTGGTGCACTTCTCCAAACGCTTTTGCCACTGTAACCCAGAAGCTCTCACCTCAG
AGGGCAAGTTTTCCATATGTATTGTGGATTTGCTGAATATTGGGAAGAAGATGTCTTGCC
AGCAGTTCATTAGCAATCTGGACGGCCTCGACGATGGCAAAGATTTTCCCAAAGAATTGC
TCAAGGTGCTCTATAACTCAATAAAGAATGAGAAGCTGGAGTGGGCGATCGAGGAAGAG
GAGTTGAGGAAGAGTCTATCGGAGCTGGTGGAGGAGCAGTGTGAGGCGGGGGGGAAGC
GGGTTGTCAGGGTAACGGATGGCAGTAACCCTTTCATCGCCATTCCTCTTTTACTCAATGC
TGTGACGTACAAACACGGGGTTCTGACACGCAAAAGCCATGCAGACATGGATGGCAAGC
GCACCCCAAGAGGACGCAGAGGCTGGAAGAAGTTTTATGCCGTGCTGAAGGGGATGATT
CTCTACCTGCAGAAAGATGAGTATAAGCCTGATAAAGACCTGTCAGAGGTGGACCTGAA
GAATGCAGTACGGGTTCACCATGCTCTTGCTACTAGGGCCTGTGACTACAGCAAGAGACA
GAACGTCCTCAAACTCAAGACTTCTGACTGGAGGGTCTTCCTACTGCAGGCCCCGAGTGA
AGAGGAGATGATGTCATGGATTTTCAGGATTAATCTAGTTGCGGCTCTTTTCTCGGCTCCC
GCTTTTCCTGCCGCCATCGGCTCCATGAAGAAATTCTGCAGACCGCTGCTGCCATCATCC
ACCACTAGACTCAACCAGGAGGAGCAACTGAAGTCCCATGAGAACAAACTGAAGCAGAT
CTCTCTGGAGTTGGAGGAACACAAGAGAAGCCCACCGGATCTGACACCCAAGAGCAAAG
AATCAGAAGAGTATCGAATCAAAGAGCACTATTTAACCTATGAGAAATGTCGCTATGAG
ACATACGTCAGCCTCCTGCAGGCCAAGTTCCGCGTGGGAAGTGACGACTTGGATAAGATT
GAAGCCAGTTTGTTTAGCGGGGAGGGGAAAGAAGGTTACCTGCGCAAAACTCAGTCTAG
CCCTTCAATCAGCCAAGGTCAGGGGGTCAATGGACGGGCC

>apolipoprotein A-I-1, CI01000092_03862519
ATGAGGTTCGTAGCCCTCGCCCTCACTGTTTTGCTGGCAGGTTGCCAGGCCCGTTTCCTGC
AGGACGAAGCACCGTCACATCTGGATCATGTGAAGTCTGTGCTCCAGGTTTATGCAGATC
AGTTAAAGCAGTCTGCACACAAGTCCCTCACTCACCTCGATGACACAGAGTTCAAAGACT
ACAAGGAATTCCTGGGCCAGTCCGTGGACAACCTCCATGGCTACTTTGAGCACGCTTTTC
AAGCCATCTCCCCAATTGGTACCCATGTGCTGGAAGCCTCTGCACCACAACGTGAGAAGC
TGACCAAGGACATCGAGGACCTCCGCAAGCAGATCGCGCCCATGCGTGAGGAGCTCAGG
CATGTTCTGCAGAAGCACTTCGAGGAGTACAGAGACGAGCTGAAGCCTTTCCTCGACGA
GTACTTGGTCAAGCAGCGTGAGCACATGGAGGAAGTCAAAACCAAGCTGGAGCCGGTGA
TCAAGAGCTTGAAGGAGAAGATTGGAACCAACTGGGAGGAGACCAAGTCCAAGCTGACG
CCCATCCTGGAAGCTGTGCGCGAAAAGTTGACTGCGTATGCGCAGGAAGTGAAGACCCA
GCTGGATCCCTACATCCAGGAATATAAGGATCAGGTGGAAAAGGGAGCCTTGGAGTTCC
GCGAGAGCGTGCGATCTGGAGAACTGAGGAAAAAGATGACCGAATTGGGCGAGCAGGT
GAAGCCTCACTTTGAGGCAATATTTGCAGCTGTCCAAAAGTCTCTTAGCAAGGAGTAA

> poly(A) binding protein interacting protein 2B, CI01000049_07639493
CCTGCAGATATTAACAGTCCAGAGGTAGCAAAGACACCAGGAGGAGGCAACGGGCAAG
GGAAGGATGAGAATGTTGTAAATGGACACAAGGAGGGGGACGCCAACCCCTTTGCAGAG
TACATGTGGATGGAGAACGAGGAGGAGTACAATAGACAGGTGGAGGAGGAACTCTTGG
AGCAGGAGTTTCTTGAGCGCTGTTTTCAGGAGATGTTGGAGGAGGAAGATCAGGATTGGT
TCATTCCTGCCAGAGACCTTCCCCCTGGAGTAGGGCAGATCCAACAGCAGCTTAGCGGGT
TGTCGGTCAGTGACGGAAACGCCGAAGACATTGCACGCAAGAGCAGCTTGAACCCAGAG

GCCAAGGAGTTCGTTCCAGGAGTGAAATACTAG

>glyceraldehyde-3-phosphate dehydrogenase, CI01000016_08888177
ATGATGTTTCTTCAATCGTTTTTCTGGTTTTCTTGGATTTACAACTTTTCTCCAGAGGACAA
GACCAAATTAGGCATCATGGTTAAAGTTGGTATCAACGGATTCGGTCGCATTGGGCGTCT
GGTGACCCGTGCTGCTTTCCAGTCCAAGAAAGTTTACATGTTCAAGTATGACTCCACCCA
TGGCAAGTACAAGGGTGAGGTTAAGGCAGAAGGCGGCAAGTTGGTCATTGACGGTCACG
CCATCTCCGTCTTCAGCGAGAGGGACCCAGCCAACATTAAATGGGGTGATGCAGGTGCC
ACATATGTTGTGGAGTCTACCGGTGTCTTCACTACCATTGAGAAGGCTTCTGCTCACCTTA
AGGGCGGTGCCAAGAGAGTCATCATCTCTGCCCCCAGTGCAGATGCCCCTATGTTTGTCA
TGGGTGTCAACCATGAGAAATATGATAATTCCCTTAAAGTTGTCAGCAATGCCTCCTGCA
CCACCAACTGCCTGGCTCCCTTGGCCAAGGTCATCAATGACAACTTTGTCATCATTGAAG
GTCTTATGAGCACTGTTCATGCCATCACAGCCACACAGAAGACCGTTGACGGGCCCTCTG
GGAAACTGTGGAGGGATGGCCGTGGTGCCAGTCAGAACATTATCCCAGCCTCCACTGGG
GCTGCCAAGGCTGTGGGCAAAGTCATTCCTGAGCTCAATGGCAAGCTTACTGGTATGGCC
TTCCGTGTCCCCACCCCCAATGTGTCTGTGGTGGATCTGACCGTCCGCCTTGAGAAACCTG
CCAAGTATGATGATATCAAGAAAGTGGTCAAGGCTGCAGCTGATGGACCCATGAAAGGC
ATTCTGGGATACACAGAGGACCAGGTTGTGTCCACTGACTTCAATGGGGATTGTCGCTCC
TCTATCTTTGATGCTGGTGCTGGCATCGCCCTCAACGATCACTTTGTCAAGCTGGTCACAT
GGTATGACAACGAGTTCGGGTACAGCAACCGTGTTTGTGACCTGATGGCACACATGGCCT
CCAAGGAGTAG

>peroxiredoxin 3, CI01180000_09883947
ATGGCAGCCACCATCGGGAGACTTCTCGGGACTTCTGCAAGAAGAGCTGCAGTTAATGG
ACTGAAGGTCTTGGTCCCTCACAATGGACCATCTGTGATCAGGGCCCCACAATCCCTTGC
CTGCATTGCTGCACAAAAAGCTTGCTTCTCAGTCAGCACTGCTAGATGGGCTGCAGCAGT
CACTCAACAGGCCCCACATTTCAAAGGCACTGCTGTTCTCAATGGAGAGTTCAAGGAGAT
CAGCCTAGAAGATTTCAAGGGCAAATACCTGGTCCTTTTCTTTTACCCACTAGATTTCACT
TTTGTTTGCCCCACAGAGATCATCGCCTTCAGTGACAAAGCCAATGAGTTTCGTGATGTA
AACTGTCAAGTAGTTGGTGTGTCTGTGGACTCTCACTTTACCCACCTGGCCTGGACCAAC
ACCCCAAGAAAGAGTGGAGGATTAGGGAAAATCCACATTCCCCTGTTGGCTGATCTCAC
AAAGCAAGTGTCCAGAGACTATGGGGTCCTGCTGGAGGGTCCTGGAATTGCTCTAAGGG
GTCTTTTCATTATTGATCCTAATGGAGTGATCCGACACATGAGTGTAAATGACCTGCCGG
TTGGACGCTCTGTTGAGGAAACCCTCCGTCTGGTCAAGGCCTTCCAGTTTGTGGAAACCC
ATGGTGAAGTCTGTCCTGCCAGCTGGACCCCAAAATCACCCACGATTAAGCCGACTCCAG
ATGGCTCAAAGGAATACTTTGAGAAGGTCAACTGA

>fructose-bisphosphate aldolase b, CI01000110_03118931
ATGACACATCAGTTTCCAGCTCTCTCTACGGAGCAGAAGAAGGAGCTTTCCACAATTGCC
CAGAGTATTGTGGCAACTGGAAAAGGCATCTTGGCTGCAGATGAGTCCACAGGCACCAT
GGCAAATCGTTTTCAGAAGATAAATGTGGAGAACACCGAAGAGAACCGTCGTAGCTTTC
GTGACCTCCTCTTCTCTGTAGACAATTCTATCTCTGAAAGTATTGGCGGTGTCATCTTTTT
CCACGAAACACTGTACCAGAAATCAGACAAAGGAGTTCTGTTTCCAAAAGTCGTCAAGG
ACAAGGGCATCGTAGTTGGTATCAAGGTGGACAAAGGCACAGCAGGCCTGGGCGGTACA
AATGGAGAGACAACCACACAAGGATTGGATGGTCTTTCTGAACGCTGTGCCCAGTACAA
GAAGGATGGTTGTGACTTTGCTAAGTGGCGTTGTGTACTCAAGATCTCTGACGGCTGCCC
CTCTGCTCTTGCAATTGCTGAAAACGCTAACGTTCTTGCCAGATATGCCAGCATTTGCCAA
CAGAATGGCTTGGTTCCCATTGTAGAGCCTGAGATCCTCCCAGACGGAGATCATGATCTG
CAACGGTGCCAGTACGCCACTGAGAAGGTCCTGGCGGCTGTGTACAAGGCTCTCTCTGAC
CACCATGTTTATCTGGAGGGAACTCTGCTCAAACCAAACATGGTCACTGCTGGACACTCC
TGCACCAAGAAGTACACCCCTCAGGAGGTTGCCATGGCAACAGTTACTGCTCTCAGACGC
ACTGTGCCAGCTGCTGTACCAGGCATCTGCTTCCTCTCTGGTGGTCAAAGTGAGGAGGAG
GCCTCTCTGAATCTGAATGCCATGAACCAGCTTCCCCTGCACAGACCCTGGAAGGTGAGC

TTCTCTTATGGCCGTGCTCTCCAGGCCTCGGCTCTTGCCGCATGGAAGGGACAAGCAGCG
AACAAGAAGGCTGCACAGGATGCCTTTGTCACACGTGCCAAGATCAATAGTCTTGCATCA
AAAGGCGAATACAAACCCTCAGGCAAGGCTGACCAAGCATCTAAACAGTCCCTCTTTAC
CGCCAGCTATGCCTACTAA

>complement factor D, CI01180000_04295052
ATGGGCGATTGGGAAAAGAATGAAAACAGTAAGAACACCCCTACACACCCTCTGCCTCT
CTCTGGGCATTATATAAAGCTCAGCCCGCACTCAGTGGTCTTCAGTCTCCGAAGTAGACA
ACAATCGTTCACTATGAATAGACTGATATTTGCCTCTTTGCTGCTCTATGCAGCATCTCAA
ACTGGCGAATGCATTACAGGAGGAAGTGAGGCTGATGCTCACTCCCGTCCGTACATGGCT
TCACTTCAGTGGAATGGAAAACATGAGTGCGGTGGCTTTCTGATCTCCAGTCAGTGGGTC
ATGAGTGCAGCACACTGCTTTCAGGACGGGAGGTCGTCCGGTGTTAAGGTTGTGTTGGGT
GCTCACTCCCTGTCTGCTGCTGAGGACACTAAACAAACATTTGGTGTTGATGTAGTCTAC
AGCCATCCTGATTTCACCATAAGCAACTATGACAATGATATTGCTCTGCTCAAGCTGGAA
AAGCCAGTCACTGAGAGTTATGCAGTGAAACCAGTGAAATTCCAACGTGATGAAGAGGC
TGATCCCAAGGAGTCTGCTGTCGTGGAAACGGCTGGATGGGGCTCTTTGAACAACCTGGG
AGGACGACCGGACAAATTGCAAGAGCTCAGTATCACGGTCATGATGCGATTTCTATGTGG
TCGCGGTGACTACTATGGAACGAAGTTCACCAGCAACATGCTCTGTGCTGCAGAAAGAC
GAAAGGACACCTGTGATGGTGACTCCGGAGGTCCTCTCTTATACAAGGGCGTTGCTGTGG
GGATAACCTCTAATGGAGGGAAGAAATGTGGCTCCACCAAAAAGCCTGGACTCTACACG
ATCATCTCCCACTACACTAGTTGGATTGACAGCATAACCACCCAGTAA
