# Supplementary material for: Global gene expression patterns of grass carp following compensatory growth
Source: BMC Genomics. 2015 Mar 14;16(1):184. doi: 10.1186/s12864-015-1427-2 (PMC4374334; doi:10.1186/s12864-015-1427-2)
Supplement: Additional file 4: — Summary of RPKM level in different samples. [file 12864_2015_1427_MOESM4_ESM.pdf]

Additional file 4 summary of RPKM level in different samples

Sample name	duplicates	RPKM interval				
		0~1	1~3	3~15	15~60	>60
**C-1-M**	a	16473(50.21%)	5392(16.43%)	7711(23.50%)	2320(7.07%)	915(2.79%)
	b	16756(51.07%)	5509(16.79%)	7429(22.64%)	2230(6.80%)	887(2.70%)
	c	17568(53.54%)	5622(17.13%)	6855(20.89%)	1943(5.92%)	823(2.51%)
**C-1-L**	a	23508(71.65%)	3575(10.90%)	3582(10.92%)	1252(3.82%)	894(2.72%)
	b	23384(71.27%)	3556(10.84%)	3634(11.08%)	1338(4.08%)	899(2.74%)
	c	23040(70.22%)	3845(11.72%)	3754(11.44%)	1288(3.93%)	884(2.69%)
**E-1-M**	a	19807(60.37%)	5506(16.78%)	5440(16.58%)	1360(4.14%)	698(2.13%)
	b	20064(61.15%)	5396(16.45%)	5335(16.26%)	1339(4.08%)	677(2.06%)
	c	18520(56.44%)	5569(16.97%)	6296(19.19%)	1656(5.05%)	770(2.35%)
**E-1-L**	a	22745(69.32%)	4113(12.54%)	4029(12.28%)	1169(3.56%)	755(2.30%)
	b	23698(72.23%)	3883(11.83%)	3506(10.69%)	1015(3.09%)	709(2.16%)
	c	25368(77.32%)	3100(9.45%)	2707(8.25%)	936(2.85%)	700(2.13%)
**C-2-M**	a	18342(55.90%)	5610(17.10%)	6500(19.81%)	1650(5.03%)	709(2.16%)
	b	17935(54.66%)	5583(17.02%)	6736(20.53%)	1794(5.47%)	763(2.33%)
	c	17964(54.75%)	5806(17.70%)	6728(20.51%)	1616(4.93%)	697(2.12%)
**C-2-L**	a	22249(67.81%)	3972(12.11%)	4235(12.91%)	1421(4.33%)	934(2.85%)
	b	23361(71.20%)	3649(11.12%)	3636(11.08%)	1270(3.87%)	895(2.73%)
	c	23251(70.86%)	3795(11.57%)	3777(11.51%)	1170(3.57%)	818(2.49%)
**E-2-M**	a	17731(54.04%)	5556(16.93%)	6682(20.37%)	2000(6.10%)	842(2.57%)
	b	18642(56.82%)	5488(16.73%)	6169(18.80%)	1734(5.28%)	778(2.37%)
	c	17697(53.94%)	5419(16.52%)	6798(20.72%)	2053(6.26%)	844(2.57%)
**E-2-L**	a	23453(71.48%)	3494(10.65%)	3617(11.02%)	1335(4.07%)	912(2.78%)
	b	23361(71.20%)	3447(10.51%)	3725(11.35%)	1352(4.12%)	926(2.82%)
	c	23054(70.26%)	3630(11.06%)	3821(11.65%)	1398(4.26%)	908(2.77%)
